# Supplementary material for: Early recognition and management of maternal sepsis in Pakistan: a feasibility study of the implementation of FAST-M intervention
Source: BMJ Open. 2023 Jul 30;13(7):e069135. doi: 10.1136/bmjopen-2022-069135 (PMC10387631; doi:10.1136/bmjopen-2022-069135)
Supplement: Supplementary data [file bmjopen-2022-069135supp001.pdf]

MODIFIED EARLY OBSTETRIC WARNING CHART (MEOWS CHART)

FAST-M

Contact clinical decision maker if patient triggers **ONE RED** or **TWO YELLOW** flags at any one time.

Patient		Patient ID		DOB/Age	

Date	
Time	
Initials	

WRITE **VALUES** IN BOXES PROVIDED

Parameter	Value	Flag
**Respiratory rate** (breaths per minute)	25 or more	RED
	21 - 24	YELLOW
	11 - 20	NORMAL
	10 or less	RED
**Oxygen saturations** (%)	95 or more	NORMAL
	94 or less OR needing oxygen	RED
**Temperature** (°C)	38 or more	YELLOW
	36.0 to 37.9	NORMAL
	35.9 or less	YELLOW
**Heart rate** (beats per minute)	120 or more	RED
	100 - 119	YELLOW
	50 - 99	NORMAL
	40 - 49	YELLOW
	39 or less	RED
**Systolic blood pressure** (mmHg)	160 or more	RED
	140 -159	YELLOW
	100 -139	NORMAL
	90 - 99	YELLOW
	89 or less	RED
**Diastolic blood pressure** (mmHg)	110 or more	RED
	90 -109	YELLOW
	40 - 89	NORMAL
	39 or less	RED
**Urine output** Hours since patient passed urine (tick box)	12 hours or less	NORMAL
	12 - 18 hours	YELLOW
	18 hours or more OR less than 0.5 ml/kg/hour	RED
**Mental State** (tick box)	Alert	NORMAL
	Not Alert	RED
**Looks unwell** (tick box)	No	NORMAL
	Yes	YELLOW
**TOTAL YELLOW FLAGS**		
**TOTAL RED FLAGS**		
ACTION TAKEN (IF REQUIRED) Yes (Y) / No (N)		

ACT NOW if patient triggers ONE RED or TWO YELLOW flags at any time. Escalate to clinical decision maker and start FAST-M decision tool.

UNITED AGAINST MATERNAL SEPSIS

THINK SEPSIS, ACT FAST-M

DECISION TOOL

FAST-M

Patient name		Staff name	
Date of birth or age		Role/Cadre	
Patient ID		Signature	
Date	_____/_____/_____	Time	______:______

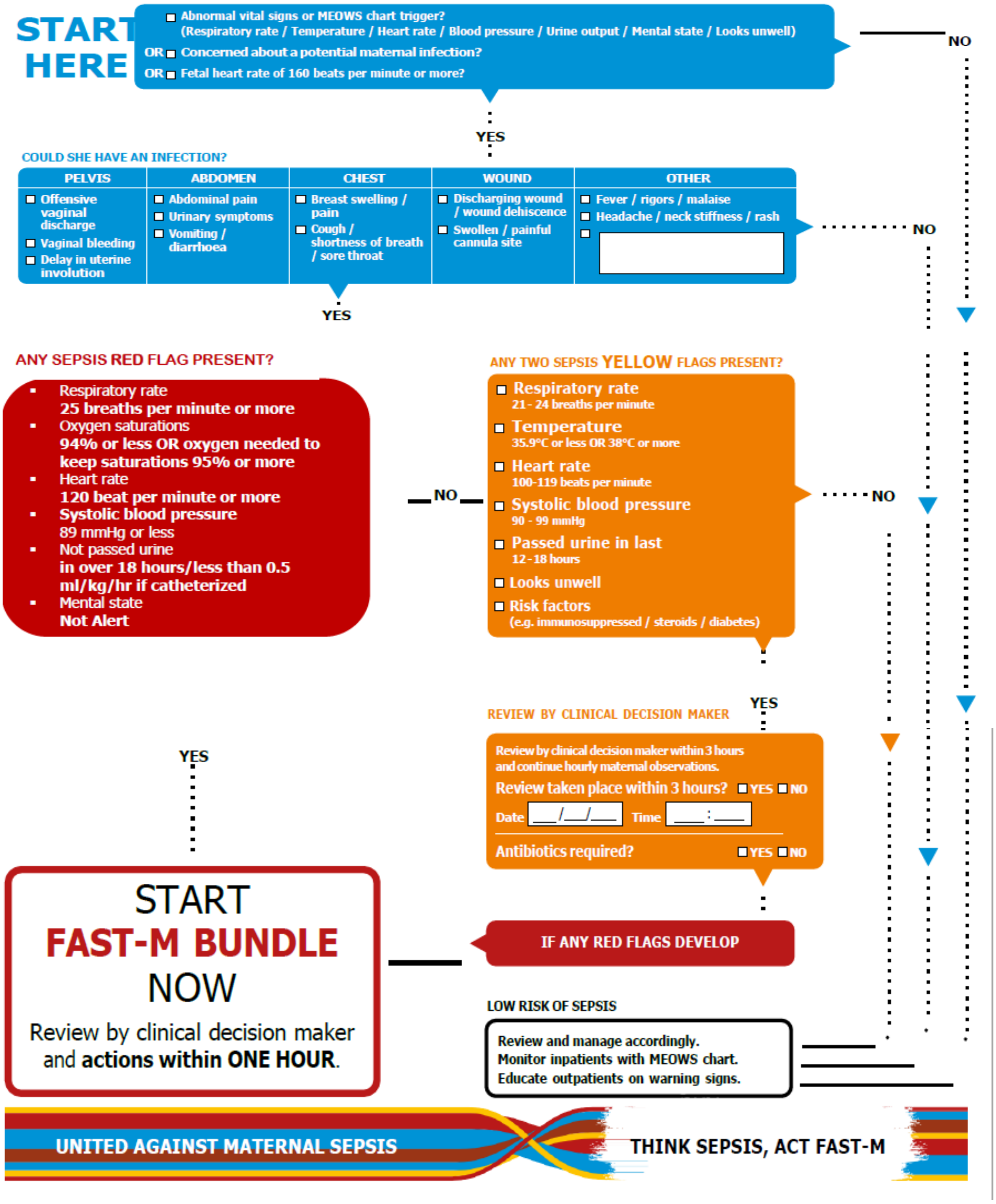

TREATMENT BUNDLE

FAST-M

REMEMBER TO COMPLETE THESE ACTIONS WITHIN ONE HOUR

Patient name		Staff name	
D.O.B or age		Role/Cadre	
Patient ID		Signature	

Date & time of red flag observation	__/__/__ __:__	Date & time bundle started	__/__/__ __:__	Date & time of review by clinical decision maker	__/__/__ __:__

F — FLUIDS (caution in pre-eclampsia, severe anaemia and pulmonary oedema)

Date	___/___/___	Time fluids initiated	____:____	Initials	
Details / reason not completed					

Give 500 ml crystalloid immediately. Repeat 500 ml boluses to a maximum of 30 ml/kg if hypotension persists.

A — ANTIBIOTICS

Date	___/___/___	Time started	____:____	Initials	
Details / reason not completed					

See antibiotic guidelines below

S — SOURCE – identify and treat the source of infection

Date	___/___/___	Time considered	____:____	Initials	
Details / reason not completed					

See source identification and treatment boxes below

T — TRANSPORT (to higher level hospital or location within hospital, if required)

Date & time transport considered	___/___/___ ___:___	Initials		Transport Required ☐ YES ☐ NO
Date & time transport requested	___/___/___ ___:___	Initials		☐ N/A
Date & time patient left facility	___/___/___ ___:___	Initials		
Destination				
Reason for any delay				

M — MONITORING (start MEOWS chart if not already started. Repeat observations every 30 minutes until otherwise decided by clinical decision maker)

Date & time monitoring commenced	___/___/___ ___:___	Details / reason not completed
Maternal / fetal monitoring should include	• Respiratory rate<br>• Oxygen Saturations<br>• Temperature<br>• Heart rate<br>• Blood pressure<br>• Urine output<br>• Mental state<br>• Fetal heart rate	
Neonatal monitoring and review commenced	☐ YES ☐ NO ☐ N/A	

ANTIBIOTIC GUIDELINES

Insert local guidance here

Immediate treatment for Maternal Sepsis:

- Ceftriaxone 2 g IV once daily (if no IV access this can be given as 2 IM injections of 1 g in different sites).
- If possible intra-abdominal source add Metronidazole 500 mg IV three times daily or 400 mg PO three times daily.

If above antibiotic regime is not available then give:

- Tazobactam 4.5 g IV daily two time a day
- Meropenem 1 g IV daily two times a day

IDENTIFY THE SOURCE

Consider

• Clinical history	• Blood cultures	• Sputum sample
• Clinical examination	• HIV and Malaria testing	• Imaging (abdominal, chest)
• Blood tests (if available) (FBC, U&Es, LFTs, CRP, clotting)	• Urine sample	• Lumbar puncture
	• Swabs (wound, vagina, throat)	• Other ______________

REMOVE / TREAT THE SOURCE

Consider

• Malaria treatment	• Removal of infected cannula / line
• Consider delivery of baby	• Hysterectomy
• Removal of retained products of conception	• Targeted antibiotics once source known
• Debridement of wound / drainage of collection	

UNITED AGAINST MATERNAL SEPSIS — THINK SEPSIS, ACT FAST-M
