# Supplementary material for: Early recognition and management of maternal sepsis in Pakistan: a feasibility study of the implementation of FAST-M intervention
Source: BMJ Open. 2023 Jul 30;13(7):e069135. doi: 10.1136/bmjopen-2022-069135 (PMC10387631; doi:10.1136/bmjopen-2022-069135)
Supplement: Supplementary data [file bmjopen-2022-069135supp002.pdf]

FORM 1: FACILITY AUDIT

FACILITY ID [ ][ ] – [ ][ ][ ]

Facility ID e.g. DH, KH *Visit ID e.g. 001, 002 etc.*

Today's Date: d d – m m – y y y y Time: h h : m m

Date previous form completed (or if first Visit, date study opened at this site): d d – m m – y y y y

Are you collecting the data during? **Baseline** ☐ **Intervention** ☐

3. How many of the following **MATERNAL OUTCOMES** have you had since the last visit or if first time since last 6 months?

Maternal Outcome	Number
Maternal Sepsis	
Maternal Deaths	
Post-Partum haemorrhage (>1L)	
Ante-Partum haemorrhage (>50ml)	
Severe pre-eclampsia/eclampsia (>160/110 and >2+protein in urine)	
Blood transfusions	
Uterine rupture	
Patient with reduced conscious level	
Injury (e.g. uterine, bladder, bowel)	
Anaphylaxis	
Pulmonary oedema	
Return to theatre	
Admission to ICU/HDU	
Broken down wounds (e.g. c/s, perineal)	

4. How many of the following **NEONATAL OUTCOMES** have you had since the last visit?

Neonatal Outcome	Number
Live Births	
Neonatal deaths before discharge from hospital	
Babies requiring antibiotics	
Sill births	
Admission to nursery care	
Apgar score of less than 7 at 5 mins after birth	

FORM 1: FACILITY AUDIT

FACILITY ID ☐☐ – ☐☐☐

Facility ID e.g. DH, KH *Visit ID e.g. 001, 002 etc.*

5. How many of the following **RESOURCES** are available today?

**Resource**	**Amount**			
	BAGS			
**Type of IV Fluids:**	0	1-5	6-10	>10
A) 0.9% Saline	☐	☐	☐	☐
B) Ringers Lactate	☐	☐	☐	☐
C) 5% dextrose	☐	☐	☐	☐
D) 50% dextrose	☐	☐	☐	☐

**Type of oral antibiotics**	**Type of oral antibiotics**			
	0	1-5	6-10	>10
A) Amoxicillin	☐	☐	☐	☐
B) Augmentin	☐	☐	☐	☐
C) Cephalosporin (e.g. Cefalexin)	☐	☐	☐	☐
D) Cefixime	☐	☐	☐	☐
E) Ciprofloxacin	☐	☐	☐	☐
F) Doxycycline	☐	☐	☐	☐
G) Clindamycin	☐	☐	☐	☐
H) Chloramphenical	☐	☐	☐	☐
I) Erythromycin	☐	☐	☐	☐
J) Metronidazole	☐	☐	☐	☐
K) Other (please state):	☐	☐	☐	☐

**Type of IM / IV antibiotics**	**VIALS**			
	0	1-5	6-10	>10
A) Amoxicillin	☐	☐	☐	☐
B) Ampicillin	☐	☐	☐	☐
C) Benzyl-penicillin	☐	☐	☐	☐
D) Penicillin G	☐	☐	☐	☐
E) Cefazolin	☐	☐	☐	☐
F) Cephalosporin (e.g. ceftriazone etc.)	☐	☐	☐	☐
G) Chloramphenical	☐	☐	☐	☐
H) Ciprofloxacin	☐	☐	☐	☐
I) Clindamycin	☐	☐	☐	☐
J) Erythromycin	☐	☐	☐	☐
K) Gentamycin	☐	☐	☐	☐
L) Metronidazole	☐	☐	☐	☐
M) Vancomycin	☐	☐	☐	☐
N) Other (please state)	☐	☐	☐	☐

**Manual vacuum aspiration (MVA) kits ready for use:**	KITS			
	0	1-5	6-10	>10
	☐	☐	☐	☐

* Good availability defined as a supply that is unlikely to run out before the next anticipated delivery

Ahmed SI, *et al. BMJ Open* 2023; 13:e069135. doi: 10.1136/bmjopen-2022-069135

FORM 1: FACILITY AUDIT

FACILITY ID ☐☐ – ☐☐☐

Facility ID e.g. DH, KH *Visit ID e.g. 001, 002 etc.*

**Resource**	**Availability**		
	**Good** *	**Limited**	**None**
Equipment for IV line (cannula, dressings etc)	☐	☐	☐
Malaria tests	☐	☐	☐
Syphilis test	☐	☐	☐
HIV test	☐	☐	☐
Functioning theatre and staff able to remove source of infection	☐	☐	☐
Working thermometers	☐	☐	☐
Working BP machines	☐	☐	☐
Working O2 saturation machines(Pulse Oximeters)	☐	☐	☐
Fetoscopes / Pinnards/ fetal stethoscopes	☐	☐	☐
Clocks/watches	☐	☐	☐
Spare batteries	☐	☐	☐
Wound swabs	☐	☐	☐
Corona virus swabs	☐	☐	☐
Urine dips	☐	☐	☐
Sputum cultures	☐	☐	☐
Blood cultures	☐	☐	☐
Pregnancy tests	☐	☐	☐
Haematology blood tests	☐	☐	☐
Biochemistry blood tests	☐	☐	☐
Corona virus tests	☐	☐	☐
Blood taking equipment	☐	☐	☐
Functioning radiology department	☐	☐	☐
Functioning ultrasound machine and person to scan	☐	☐	☐
Functioning theatre and staff able to remove source of infection	☐	☐	☐
Weighing scales	☐	☐	☐
Urinary catheters	☐	☐	☐
Gloves	☐	☐	☐
Aprons	☐	☐	☐

6. How many of the following **STAFF** is available TODAY?

**Resource**	**Number**
Doctors	
Medical Officers	
Medical Students	
Registered Nurse	
Nursing students	
Hospital and Ward Attendants	

Ahmed SI, *et al. BMJ Open* 2023; 13:e069135. doi: 10.1136/bmjopen-2022-069135

FORM 1: FACILITY AUDIT

FACILITY ID [ ][ ] – [ ][ ][ ]

Facility ID e.g. DH, KH *Visit ID e.g. 001, 002 etc.*

7. Are the following **FACILITIES** in working order TODAY?

Resource	Yes	No
Does the ward have electricity?		
Does the ward have running water?		
Does the ward have soap?		
Does the ward have alcohol gel?		
Are the autoclaves working?		
Are the washing facilities for staff available?		
Are the washing facilities for patients available?		
Are flushable toilet facilities for staff available?		
Are flushable toilet facilities for patients available?		

* Good availability defined as a supply that is unlikely to run out before the next anticipated delivery

Completed by: ______________________

Role: ______________________

Signature: ______________________

____

Date: DD / MMM / YYYY

You must have signed the Site Signature & Delegation Log

Ahmed SI, *et al. BMJ Open* 2023; 13:e069135. doi: 10.1136/bmjopen-2022-069135
