# Supplementary material for: Early recognition and management of maternal sepsis in Pakistan: a feasibility study of the implementation of FAST-M intervention
Source: BMJ Open. 2023 Jul 30;13(7):e069135. doi: 10.1136/bmjopen-2022-069135 (PMC10387631; doi:10.1136/bmjopen-2022-069135)
Supplement: Supplementary data [file bmjopen-2022-069135supp003.pdf]

FORM 2: PATIENT CARE AUDIT FORM

Patient ID Number: ☐☐-☐☐☐

Facility ID e.g. A01, A02 etc *Unique ID e.g. 001, 002 etc*

Baseline ☐ **or** Intervention ☐

Date of audit: d d - m m - y y y y

Time of audit: h h : m m

1. Clinical status at admission:

Antenatal ☐

Miscarriage ☐

Post-natal ☐

Termination of pregnancy ☐

2. Which of the following observations were recorded when the patient was first assessed?

	Yes	No	Not recorded
**Respiratory rate**			
**Saturations**			
**Temperature**			
**Heart rate**			
**Systolic BP**			

	Yes	No	Not recorded
**Diastolic BP**			
**Urine output**			
**Mental State**			
**Fetal heart rate**			

3. Were the observations recorded on a chart?

Yes ☐ No ☐ N/A ☐

→ If intervention phase, were they recorded on a MEOWS chart?

Yes ☐ No ☐

FORM 2: PATIENT CARE AUDIT FORM **PATIENT ID NUMBER:** ☐☐☐ - ☐☐☐☐

4. Has the patient been admitted for the last 24 hours

Yes ☐ No ☐

If yes, go to question 5, if No got to Question 6

5. During the last 24 hours, please state the number of times each observation has been taken and charted:

	Number of times
Respiratory rate	☐
Oxygen saturation	☐
Temperature	☐
Heart rate	☐
Systolic BP	☐

	Number of times
Diastolic BP	
Urine output	
Mental state	
Fetal heart rate	

REVIEW EVERY PATIENT'S NOTES WITH AN ABNORMAL OBSERVATION AND CONSIDER COMPLETING FORM 3 IF THERE IS CONCERN ABOUT MATERNAL INFECTION

Completed by: ______________________

Role: ______________________

Signature: ______________________ Date: DD / MMM / YYYY

You must have signed the Site Signature & Delegation Log

Ahmed SI, *et al. BMJ Open* 2023; 13:e069135. doi: 10.1136/bmjopen-2022-069135
