# Supplementary material for: Early recognition and management of maternal sepsis in Pakistan: a feasibility study of the implementation of FAST-M intervention
Source: BMJ Open. 2023 Jul 30;13(7):e069135. doi: 10.1136/bmjopen-2022-069135 (PMC10387631; doi:10.1136/bmjopen-2022-069135)
Supplement: Supplementary data [file bmjopen-2022-069135supp004.pdf]

FORM 3: INDIVIDUAL PATIENT FORM

Screen the notes for every patient that had a suspected infection.
Only complete this form for patients that fulfil the criteria in q1-4 below.

Also complete this form for any patient you observe during the patient care audit (Form2) that fulfils the criteria below.

CONSIDER QUESTIONS 1-4 TO DETERMINE IF THIS FORM NEEDS TO BE COMPLETED

1. Was there a concern about a potential maternal infection?

   Yes ☐ No ☐ → **DO NOT COMPLETE FORM**

   Date and time suspected: d d - m m - y y y y   h h : m m

2. Have observations taken?

   Yes ☐ No ☐ → **COMPLETE FORM**

3. Was there a fetal tachycardia (heart rate) greater than or equal to 160 bpm?

   Yes ☐ No ☐ Not Recorded ☐

4. Did the patient have abnormal vital signs or MEOWS chart trigger - 1 or more red flags or 2 or more yellow flags?

   Yes ☐ No ☐

IF NO TO BOTH QUESTIONS, DO NOT COMPLETE FORM

FORM 3: INDIVIDUAL PATIENT FORM

Patient ID number: ☐☐☐☐ ☐☐☐☐

Date of assessment medical record: d D / m m / y y y y

Time of assessment medical record: ☐☐ ☐☐

5. **Study phase:**

Baseline ☐ Intervention ☐

6. At the time of assessment for maternal infection by the health care staff, were they an inpatient or outpatient?

Inpatient ☐ Outpatient ☐

→ Date of admission: d d - m m - y y y y

→ Whilst an inpatient, was a chart used for the patient? FAST-M MEOWS chart: ☐ Other chart ☐

If no, why not? ____________________

____

7. Were the patient referred from health facility

Yes ☐ No ☐

8. What treatment(s) did the patient receive before being referred? (Tick all the boxes that have been collected)

Fluids ☐ Antibiotics ☐ Source control ☐ Monitoring ☐ Not recorded ☐

9. What was the patient's pregnancy status when infection was first suspected/diagnosed?

<12 weeks ☐ 28+ weeks ☐ Miscarriage/ Abortion within the last 6 weeks ☐

12-28 ☐ Post-natal (up to 6 weeks) ☐

10. What type of infection was suspected? (Tick one or more options)

Offensive vaginal discharge	☐	Breast swelling/pain	
Vaginal bleeding	☐	Cough/shortness of breath/sore throat	☐
Delay in uterine involution	☐	Discharging wound/wound dehiscence	☐
Abnormal pain	☐	Swollen /painful cannula site	☐
Urinary symptoms	☐	Fever /rigors/malaise	☐
Vomiting/diarrhoea	☐	Headache/Neck stiffness/rash	☐
		Others, Please state ________________	

Ahmed SI, *et al. BMJ Open* 2023; 13:e069135. doi: 10.1136/bmjopen-2022-069135

FORM 3: INDIVIDUAL PATIENT FORM **PATIENT ID NUMBER** ☐☐☐-☐☐☐

11. Did the patient have any of the following observations at time infection was first suspected/diagnosed (see q1)?

	Yellow trigger		Red trigger		No abnormal Observation	Not recorded
Respiratory rate	21-24		25 or more	10 or less	11 - 20	
	☐		☐	☐	☐	☐
Oxygen Saturations			94 or less Or needing oxygen		95 or more	
			☐		☐	☐
Temperature	38 or more	35.9 or less			36.0 to 37.9	
	☐	☐			☐	☐
Heart rate	100-119	40-49	120 or more	39 or less	50 – 99	
	☐	☐	☐	☐	☐	☐
Systolic Blood Pressure	140-159	90-99	160 or more	89 or less	100 -139	
	☐	☐	☐	☐	☐	☐
Diastolic Blood Pressure	90-109		110 or more	39 or less	40 – 89	
	☐		☐	☐	☐	☐
Not passed urine	In last 12-18 hours		In over 18 hours or less than 0.5ml/kg/hr.		12 hours or less	
	☐		☐		☐	☐
Appearance	Looks unwell				Look well	
	☐				☐	☐
Mental state			Not alert		Alert	
			☐		☐	☐

Date of observation [d d]-[m m]-[y y y y]

☐ or tick if not recorded

Time of observation [h h] [m m]

☐ or tick if not recorded

3

FORM 3: INDIVIDUAL PATIENT FORM **PATIENT ID NUMBER** ☐☐☐-☐☐☐

12. At the time the decision tool was initiated (if intervention phase) or if not used that the infection was most serious there were:

    Yellow flags ___ (enter number)

    Red flags ___ (enter number)

    If 2 or more yellow flags, or 1 or more red flags was the FAST-M bundle initiated (if intervention phase)?

    Yes ☐ No ☐

13. If one or more red flags present? Was the FAST-M bundle initiated (within 1 hour) (if intervention phase)?

    Yes ☐ No ☐
    Not recorded ☐ Go to question 12

14. If 2 or more yellow flags were present, did a review take place within 3 hours?

    Yes ☐ No ☐ Not recorded ☐

15. Was the patient an inpatient for 24 hours or more prior to infection being suspected/ diagnosed?

    Yes ☐ No ☐

16. If yes, During the last 24 hours or more, please state the number of times each observation has been recorded prior to infection being suspected / diagnosed:

	Number of times
Respiratory rate	
Temperature	
Heart rate	
Systolic BP	

	Number of times
Diastolic BP	
Urine output	
Mental state	
Fetal heart rate	

FORM 3: INDIVIDUAL PATIENT FORM **PATIENT ID NUMBER** ☐☐☐-☐☐☐

17. At the first time that a yellow or red trigger occurred, was action taken within 3 hours (if two or more yellow flags) or within 1 hour (if red)?

Yes ☐ No ☐ Not Recorded ☐

18. What action was taken (answer yes or no for each option)?

	Yes	No
Medical Officer informed	☐	☐
Nurse in charge informed	☐	☐
Resident Doctor informed	☐	☐
Treatment started	☐	☐
Consultant Doctor Informed	☐	☐
Nurse	☐	☐
Decision tool started, if intervention phase?	☐	☐

Others (please state) ________________

19. Who took action (answer yes or no for each option)?

	Yes	No
	☐	☐
Medical Officer	☐	☐
Resident Doctor	☐	☐
Consultant Doctor		
Medical assistant	☐	☐

	Yes	No
Nurse	☐	☐
Nurse In-charge	☐	☐

20. If the patient was in an outpatient setting was the patient subsequently admitted?

Yes ☐ No ☐ N/A ☐

21. Was the patient commenced on an observation chart?

Yes ☐ No ☐

FORM 3: INDIVIDUAL PATIENT FORM **PATIENT ID NUMBER** ☐☐☐-☐☐☐

22. In the 24 hours or more after suspecting maternal infection, did the patient have their observations repeated WHILST they were awaiting the clinical review?

Yes ☐ No ☐ No, because review happened at the same time ☐

Not Done ☐

	½ Hourly	Hourly	Less Frequent than hourly
Respiratory rate			
Temperature			
Heart rate			
Systolic BP			

	½ Hourly	Hourly	Less Frequent than hourly
Diastolic BP			
Urine output			
Mental state			
Fetal heart rate			

23. Did the patient receive a clinical review within 1 or 3 hours' review of recognition of maternal infection/sepsis?

Yes ☐ No ☐

Date and time of review d d - m m - y y y y h h : m m

Ahmed SI, *et al. BMJ Open* 2023; 13:e069135. doi: 10.1136/bmjopen-2022-069135

FORM 3: INDIVIDUAL PATIENT FORM **PATIENT ID NUMBER** ☐☐☐-☐☐☐

a. Did the patient receive a clinical review within 3 hours? Yes ☐ No ☐
b. Did the patient receive a clinical review within 1 hour? Yes ☐ No ☐

24. Following the clinical review was the specified frequency of observations given?
Yes ☐ No ☐

25. Did the patient undergo observations at this specified frequency?
Yes ☐ No ☐

26. Was the patient treated for maternal infection/sepsis?
Yes ☐ No ☐

27. If intervention phase was the FAST-M decision tool was used for the patient?
Yes ☐ No ☐

MANAGEMENT

28. Were IV fluids given?

Yes ☐ No ☐ Why not? ______________________________

Date of started: d d - m m - y y y y Time of started h h m m

☐ or tick if not recorded ☐ or tick if not recorded

29. Were antibiotics administered within one hour?

Yes ☐ No ☐ Why not? ______________________________

Date of administered: d d - m m - y y y y Time of administered: h h m m

☐ or tick if not recorded ☐ or tick if not recorded

Ahmed SI, *et al. BMJ Open* 2023; 13:e069135. doi: 10.1136/bmjopen-2022-069135

FORM 3: INDIVIDUAL PATIENT FORM **PATIENT ID NUMBER** ☐☐☐-☐☐☐

SOURCE CONTROL/ IDENTIFICATION

30. Was an attempt made to identify the source of infection?

Yes ☐ No ☐ Why not? ____________________

Date: d d - m m - y y y y Time: h h m m

☐ or tick if not recorded ☐ or tick if not recorded

What attempts were made to identify the source?

	Yes	No
Clinical history	☐	☐
Clinical examination	☐	☐
Blood test (if available)	☐	☐
Blood cultures	☐	☐
HIV test	☐	☐
Malaria test	☐	☐
Urine sample	☐	☐
Swabs (wound, vagina, throat)	☐	☐
Imaging (abdominal, chest)	☐	☐
Lumbar puncture	☐	☐
Sputum sample	☐	☐
Others (please state) ____________		

31. Was the source identified?

Yes ☐ No ☐

Date: d d - m m - y y y y Time: h h m m

☐ or tick if not recorded ☐ or tick if not recorded

Ahmed SI, *et al. BMJ Open* 2023; 13:e069135. doi: 10.1136/bmjopen-2022-069135

FORM 3: INDIVIDUAL PATIENT FORM **PATIENT ID NUMBER** ☐☐☐-☐☐☐

What was the source of infection?

	Yes	No
Breast	☐	☐
Chorioamnionitis	☐	☐
Endometritis	☐	☐
Infected Cannula/ line	☐	☐
Malaria	☐	☐
Meningitis/ cerebral infection	☐	☐
Other wound/ skin infection	☐	☐
Other (please state)____________________		
Peritonitis	☐	☐
Respiratory infection	☐	☐
Urinary Tract Infection	☐	☐

32. Was the source treated?

Yes ☐ No ☐ Why not? __________________________

Date: d d - m m - y y y y ☐ or tick if not recorded

Time: h h m m ☐ or tick if not recorded

How was it treated?

	Yes	No
Delivery of the baby	☐	☐
Removal of retained products of conception	☐	☐
Hysterectomy	☐	☐
Removal of infected cannula/line	☐	☐
Targeted antibiotics	☐	☐
Malaria treatment	☐	☐
Antivirals	☐	☐
Incision and drainage	☐	☐
Percutaneous drainage	☐	☐
Debridement of wound	☐	☐
Laparotomy and washout	☐	☐
Other (Please state) ________________		

Ahmed SI, *et al. BMJ Open* 2023; 13:e069135. doi: 10.1136/bmjopen-2022-069135

FORM 3: INDIVIDUAL PATIENT FORM **PATIENT ID NUMBER** ☐☐☐-☐☐☐

TRANSPORT

33. Was there a need to transport the patient to a higher level of care considered?

Yes ☐ No ☐ Why not? ______________________ Not recorded ☐

Date: d d - m m - y y y y Time: h h m m

☐ or tick if not recorded ☐ or tick if not recorded

34. Was the patient transferred?

Yes ☐ No ☐ Why not? ______________________ Not recorded ☐

Date requested: d d - m m - y y y y Time requested: h h m m

☐ or tick if not recorded ☐ or tick if not recorded

Date patient left the facility: d d - m m - y y y y Time: h h m m

☐ or tick if not recorded ☐ or tick if not recorded

35. Where was the patient transferred to? ____

36. Was a transfer letter sent with the patient?

Yes ☐ No ☐ Why not? ______________________

Ahmed SI, *et al. BMJ Open* 2023; 13:e069135. doi: 10.1136/bmjopen-2022-069135

FORM 3: INDIVIDUAL PATIENT FORM **PATIENT ID NUMBER** ☐☐☐-☐☐☐

MONITORING

37. Did the patient have ongoing monitoring commenced after the recognition of maternal suspected/sepsis?

Yes ☐ No ☐ → N/A (24hrs hadn't elapsed) ☐

	1/2 hourly or Less	Hourly or Less	Daily or More	Less than Daily	not done
Respiratory rate	☐	☐	☐	☐	☐
Temperature	☐	☐	☐	☐	☐
Heart rate	☐	☐	☐	☐	☐
Systolic BP	☐	☐	☐	☐	☐

	½ hourly or Less	Hourly or Less	Daily or More	Less than Daily	not done
Diastolic BP	☐	☐	☐	☐	☐
Urine output	☐	☐	☐	☐	☐
Neuro Response	☐	☐	☐	☐	☐
Fetal heartrate	☐	☐	☐	☐	☐

Ahmed SI, *et al. BMJ Open* 2023; 13:e069135. doi: 10.1136/bmjopen-2022-069135

FORM 3: INDIVIDUAL PATIENT FORM **PATIENT ID NUMBER** ☐☐☐-☐☐☐

PATIENT OUTCOMES

38. Did the patient have any of the following complications recorded during their treatment?

	Yes	No
Pulmonary oedema	☐	☐
Blood transfusion	☐	☐
Return to theatre	☐	☐
Allergic reaction	☐	☐

	Yes	No
Change in mental state	☐	☐
Reduced urine output (no output for >18 hours or <0.5ml/kg/hr)	☐	☐
Other	☐	☐
Please state	☐	☐

39. Was the patient admitted to high dependency/ intensive care during their treatment?

Yes ☐ No ☐

No. of days: ☐ Days

40. Did the patient have any of the following near miss events recorded during their stay?

Cardiovascular dysfunction		
**Event**	**Yes**	**No**
Shock	☐	☐
Cardiac arrest	☐	☐
Severe hypo perfusion (lactate >5mmol/L)	☐	☐
Severe acidosis (pH<7.1)	☐	☐
Use of continuous vasoactive drugs	☐	☐
Cardio-pulmonary resuscitation	☐	☐
**Respiratory dysfunction**		
Acute cyanosis	☐	☐
Gasping	☐	☐
Severe tachypnea (respiratory rate >40)	☐	☐
Severe bradypnea (respiratory rate <6)	☐	☐
Severe hypoxaemia (O2 sats <90% for >60 mins or PAO2/FiO2 <200)	☐	☐
Intubation and ventilation not related to anaesthesia	☐	☐

Ahmed SI, *et al. BMJ Open* 2023; 13:e069135. doi: 10.1136/bmjopen-2022-069135

FORM 3: INDIVIDUAL PATIENT FORM **PATIENT ID NUMBER** ☐☐☐-☐☐☐

PATIENT OUTCOMES

Renal dysfunction		
Oliguria non-responsive to fluids or diuretics	☐	☐
Creatinine >300 µmol/ml or >3.5 mg/dL	☐	☐
Dialysis for acute renal failure	☐	☐
**Coagulation dysfunction**		
Failure to form clots	☐	☐
Severe acute thrombocytopenia (50,000 platelets/ml)	☐	☐
Massive transfusion of blood or red cells (≥5 units)	☐	☐
**Hepatic dysfunction**		
Jaundice in the presence of pre-eclampsia	☐	☐
Severe acute hyperbilirubinemia (bilirubin>100 µmol/L or >6.0 mg/dL)	☐	☐
**Neurological dysfunction**		
Prolonged unconsciousness or coma (lasting >12 hours)	☐	☐
Stroke	☐	☐
Uncontrollable fit / status epilepticus	☐	☐
Global paralysis	☐	☐
**Uterine dysfunction**		
Hysterectomy due to uterine infection or haemorrhage	☐	☐

FORM 3: INDIVIDUAL PATIENT FORM **PATIENT ID NUMBER** ☐☐☐-☐☐☐

PATIENT OUTCOMES

41. Date of final discharge from hospital/health centre: d d - m m - y y y y

42. Total number of days spent in hospital: ☐ days

43. What was the outcome of the patient at discharge?

Alive and well, no follow-up required ☐

Alive and well, follow-up arranged ☐

Death ☐

Date of death: d d - m m - y y y y

Time of Death: h h : m m

Cause of death: ____________________

PREGNANCY OUTCOMES (if event occurred during same admission)

Tick if not applicable ☐

44. What was the pregnancy outcome?

Miscarriage ☐

Ectopic pregnancy ☐

Induced abortion ☐

Date occurred or diagnosed: d d - m m - y y y y

Live birth ☐

Still birth (> 28 weeks) ☐

Pregnancy terminated before admission ☐

Ahmed SI, *et al. BMJ Open* 2023; 13:e069135. doi: 10.1136/bmjopen-2022-069135

FORM 3: INDIVIDUAL PATIENT FORM **PATIENT ID NUMBER** ☐☐☐-☐☐☐

45. Was monitoring of the new-born commenced?

Yes ☐ No ☐ N/A ☐

Date: d d - m m - y y y y

☐ or tick if not recorded

Time: h h m m

☐ or tick if not recorded

46. Was fetal monitoring done if the patient was antenatal?

Yes ☐ No ☐ N/A ☐

Date: d d - m m - y y y y

☐ or tick if not recorded

Time: h h m m

☐ or tick if not recorded

Ahmed SI, *et al. BMJ Open* 2023; 13:e069135. doi: 10.1136/bmjopen-2022-069135

FORM 3: INDIVIDUAL PATIENT FORM

PATIENT ID NUMBER ☐☐☐-☐☐☐

NEONATAL OUTCOMES AT BIRTH

Tick if not applicable ☐

47. Gestation: ☐ Months OR ☐ weeks ☐ days

48. Weight: ☐ g Not recorded ☐

49. Did the baby have any of the following complications?

	Yes	No	Not recorded
Resuscitation	☐	☐	☐
Agpars below 7 at 5 mins after birth	☐	☐	☐
Admitted to neonatal unit/special care	☐	☐	☐
Other (Please state)	☐	☐	________

50. Was the baby thought to have sepsis?

Yes ☐ No ☐

(If No) Did the baby receive antibiotic prophylaxis anyway?

Yes ☐ No ☐

(If Yes) Were they treated for sepsis?

Yes ☐ No ☐ Not recorded

51. What was the outcome of the baby at discharge?

Alive and well, no follow-up required ☐

Alive and well, follow-up required ☐

Death ☐

Date of Death d d - m m - y y y y

Time of Death: h h m m

Cause of Death ________________

FORM 3: INDIVIDUAL PATIENT FORM **PATIENT ID NUMBER** ☐☐☐-☐☐☐

Completed by: ______________________________

Role: ______________________________

Signature: ______________________________ Date: *DD* / *MM* / *YYYY*

You must have signed the Site Signature & Delegation Log

Ahmed SI, *et al. BMJ Open* 2023; 13:e069135. doi: 10.1136/bmjopen-2022-069135
