# Supplementary material for: Early recognition and management of maternal sepsis in Pakistan: a feasibility study of the implementation of FAST-M intervention
Source: BMJ Open. 2023 Jul 30;13(7):e069135. doi: 10.1136/bmjopen-2022-069135 (PMC10387631; doi:10.1136/bmjopen-2022-069135)
Supplement: Supplementary data [file bmjopen-2022-069135supp005.pdf]

Supplemental File 5

APPENDIX FIGURE 14 – Participant Study ID Number: ___/_____– PATIENT CONSENT FORM (ENGLISH) for patients agreeing to access of their notes

Title of study: Extension of the FAST- M maternal sepsis bundle in Pakistan, a feasibility study

What is the purpose of the overall study?

We are developing a way of improving the care of women with pregnancy or birth related infections around the world.

We are introducing three things: 1. a chart to help the nurses record your vital signs and detect infection before it becomes worse 2. A tool to help the medical staff treat infection 3. A training day to teach the medical staff about pregnancy or birth related infections

We hope that this will make caring for patients with pregnancy or birth related infections easier.

Why have I been invited to participate?

We are asking all patients pregnant or recently pregnant patients who are receiving treatment in hospital or who have an infection to participate. Being invited to take part does not necessarily mean you have an infection.

What will I have to do if I take part?

Nothing. If you agree to take part, you will receive the normal care. All we ask is that our research team from Aga Khan University Hospital can look at your medical notes to see what happened to you whilst you were in hospital. The information from your medical notes will help us decide if our study is working.

Do I have to take part?

No you do not have to take part it is your decision. If you don't want to take part that is fine, it will not affect how you are cared for when you are in hospital.

Will my taking part in the study be kept confidential?

Yes. All the information from your notes will be stored securely on a password protected computer system, which will be backed-up in a separate location to keep this information safe.

What are the possible benefits of taking part?

By giving us permission to look at your notes we hope that we will be able to find out if the study is working. If the study works, then this will hopefully help to improve the care of mothers with infections.

What will happen if I don't want to carry on with the study?

You may withdraw from the study without giving a reason for up to one month after. If you wish to withdraw we will delete all the information we had collected about you.

What if there is a problem?

If you have a concern about any aspect of this study, you can speak to the researchers, who will do their best to answer your questions. Their contact details are on the next page.

Who is organizing the research?

This study is being carried out by the Aga Khan University Hospital(AKUH), Pakistan in affiliation with University of Birmingham, UK and University of Liverpool, UK.

AKUH team will be facilitating the day to day running of the study, including looking at your medical notes and collecting the information.

Who has reviewed the study?

This study has been reviewed by the National Bioethics Committee Pakistan and AKUH Research Ethics Committee.

Contact details:

Lead Facilitator: Dr Sheikh Irfan Ahmed, Senior Instructor, AKUH National stadium road, Karachi

Email: sheikh.irfan@aku.edu Telephone number: +92-021-34864650

Registered study ID number:

PLEASE INITIAL THE BOXES IF YOU AGREE WITH EACH SECTION:

1. I understand that my participation is voluntary and that I am free to withdraw up to one month after my participation without giving any reason. ☐
2. I agree for my notes to be accessed by the AKUH research team. I understand that all data collected about me will be anonymised and will not contain any identifiable information. ☐
3. I understand that the researchers might publish the results of this study. I give permission for anonymized information from my notes to be used for this purpose. ☐
4. I know how to contact the research team if I need to. ☐
5. I am happy for information about me might also be retained to include it anonymously in future studies
6. I agree to participate in this study. ☐

SIGNATURES:

___________________________ ___________ _______________________ Patient name and surname

Date Signature / Thumbprint

___________________________ ___________ _______________________ Name and Surname of person

Date Signature taking consent

Participant Study ID Number: _____/_____
