# Supplementary figures and images for: Early recognition and management of maternal sepsis in Pakistan: a feasibility study of the implementation of FAST-M intervention
Source: BMJ Open. 2023 Jul 30;13(7):e069135. doi: 10.1136/bmjopen-2022-069135 (PMC10387631; doi:10.1136/bmjopen-2022-069135)

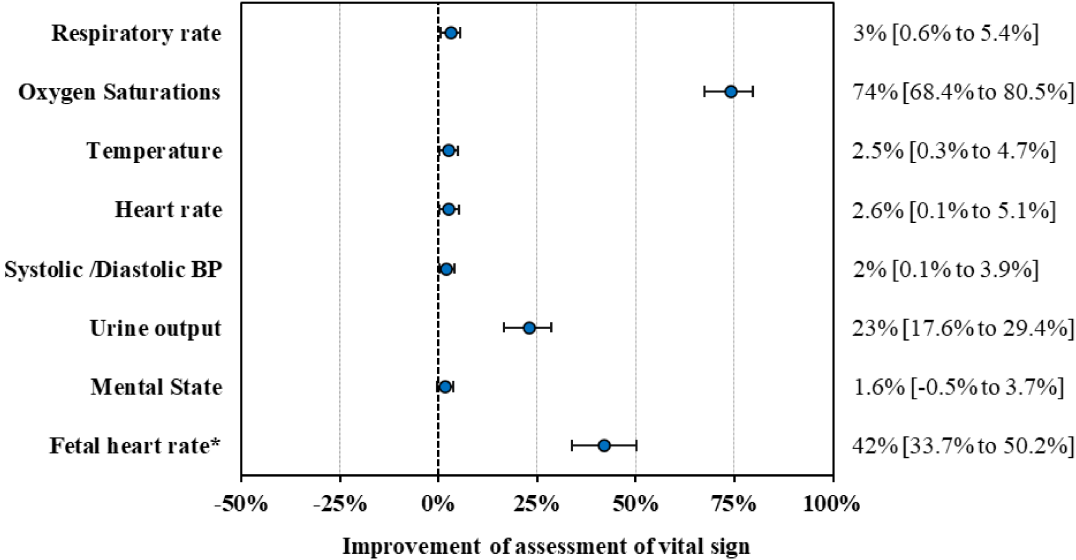

Supplement Figure 6: Patient’s assessment of vital signs on admission (n=439)

Supplement: Supplementary data [file bmjopen-2022-069135supp006.pdf]

Supplemental file 9

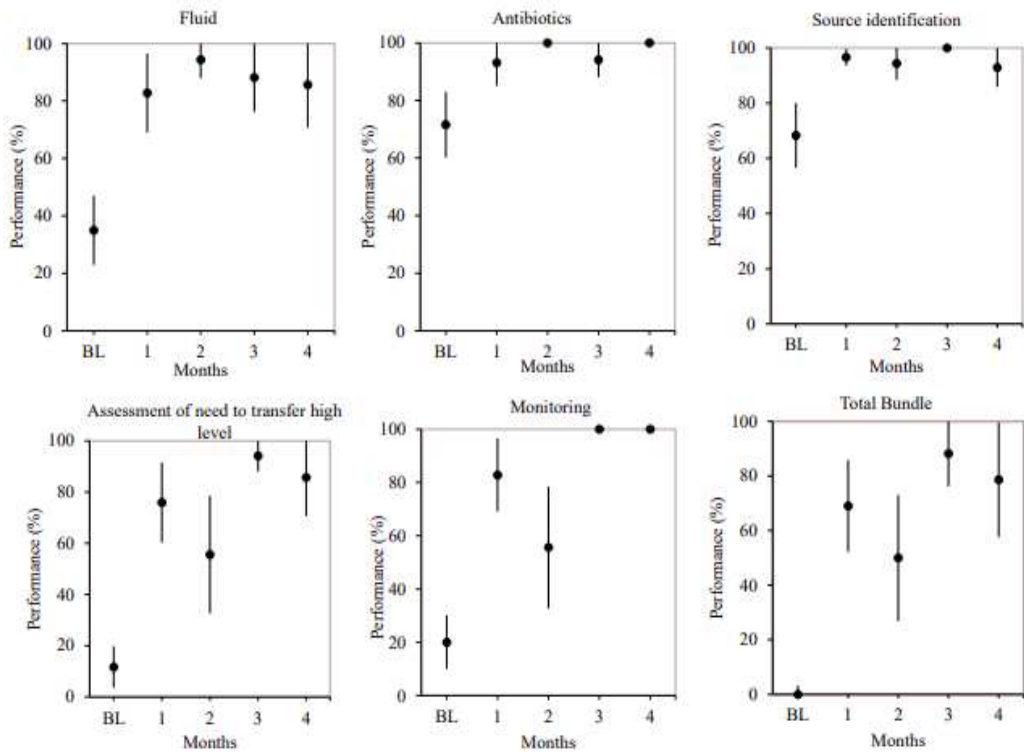

Supplement: Supplementary data [file bmjopen-2022-069135supp009.pdf]
