# Supplementary material for: Early recognition and management of maternal sepsis in Pakistan: a feasibility study of the implementation of FAST-M intervention
Source: BMJ Open. 2023 Jul 30;13(7):e069135. doi: 10.1136/bmjopen-2022-069135 (PMC10387631; doi:10.1136/bmjopen-2022-069135)
Supplement: Supplementary data [file bmjopen-2022-069135supp007.pdf]

Supplemental File 7

Table: Characteristics of suspected maternal sepsis patients (n=138)

Variables	Baseline	Intervention
	n=60	n=78
**Gestational at presentation**		
<12 weeks	2(3.3%)	0(0%)
12-28 weeks	2(3.3%)	1(1.3%)
>28 weeks	31(51.7%)	38(48.7%)
Post-natal (up to 6 weeks)	25(41.7%)	39(50%)
**Abnormal Trigger**		
Respiratory Rate (>25 or <10 per minute)	41(68.3%)	41(52.6%)
Temp (>38 or <35.9 °C)	25(41.7%)	35(44.9%)
Heart Rate (>120 or <39 beats per minute)	32(53.3%)	38(48.7%)
Systolic Blood Pressure (>160 or <89 mmhg)	43(71.7%)	43(55.1%)
Diastolic Blood Pressure (>110 or <39 mmhg)	35(58.3%)	38(48.7%)
Urine Output (In over 18 hours or less than 0.5ml/kg/hr)	4(6.7%)	4(5.1%)
Mental State (Not alert)	9(15%)	28(35.9%)
Appearance (Looks unwell)	7(11.7%)	49(62.8%)
**Source of maternal infection/sepsis***		
Breast	1(1.7%)	1(1.3%)
Chorioamnionitis	13(21.7%)	7(9%)
Endometritis	4(6.7%)	2(2.6%)
Infected Cannula/ line	1(1.7%)	0(0%)
Malaria	0(0%)	5(6.4%)
Meningitis/ cerebral infection	1(1.7%)	0(0%)
Other wound/ skin infection	3(5%)	5(6.4%)
Peritonitis	2(3.3%)	7(9%)
Respiratory infection	29(48.3%)	14(17.9%)
Urinary Tract Infection	9(15%)	6(7.7%)
Others	13(21.7%)	31(39.7%)
Unknown	7(11.7%)	14(17.9%)

*Thirty patients had multiple infection
