# Supplementary material for: Early recognition and management of maternal sepsis in Pakistan: a feasibility study of the implementation of FAST-M intervention
Source: BMJ Open. 2023 Jul 30;13(7):e069135. doi: 10.1136/bmjopen-2022-069135 (PMC10387631; doi:10.1136/bmjopen-2022-069135)
Supplement: Supplementary data [file bmjopen-2022-069135supp008.pdf]

Supplemental file 8

Table: Laboratory investigations of suspected maternal sepsis patients

Laboratory Investigations	Baseline phase (n=60)	Intervention phase (n=78)
Blood test (if available)	55 (92%)	78 (100%)
Blood cultures	27 (45%)	67 (86%)
HIV test	52 (87%)	72 (92%)
Malaria test	1 (2%)	21 (27%)
Urine sample	10 (17%)	63 (81%)
Swabs (wound, vagina, throat)	9 (15%)	2 (3%)
Imaging (abdominal, chest)	8 (13%)	38 (49%)
Lumbar puncture	0 (0%)	2 (3%)
Sputum sample	2 (3%)	2 (3%)
