# Supplementary material for: Early recognition and management of maternal sepsis in Pakistan: a feasibility study of the implementation of FAST-M intervention
Source: BMJ Open. 2023 Jul 30;13(7):e069135. doi: 10.1136/bmjopen-2022-069135 (PMC10387631; doi:10.1136/bmjopen-2022-069135)
Supplement: Supplementary data [file bmjopen-2022-069135supp010.pdf]

Supplemental file 10

Table: Assessment of Maternal statistics (from facility audit CRF1)

Total Admissions (N=24,000)	January to June 2021 (n=11205)	July to December 2021 (n=12795)	P-value
Maternal Sepsis	210 (1.8%)	160 (1.2%)	.0005
Maternal Mortality	159 (1.4%)	120 (0.9%)	.001
Post-Partum Hemorrhage	110 (0.9%)	175 (1.36%)	.006
Antepartum Hemorrhage	194 (1.73%)	110 (0.85%)	.0005
Severe Pre-Eclampsia	133 (1.18%)	88 (0.68%)	.0005
Uterine Rupture	36 (0.32%)	28 (0.21%)	.125
