# Supplementary material for: Early recognition and management of maternal sepsis in Pakistan: a feasibility study of the implementation of FAST-M intervention
Source: BMJ Open. 2023 Jul 30;13(7):e069135. doi: 10.1136/bmjopen-2022-069135 (PMC10387631; doi:10.1136/bmjopen-2022-069135)
Supplement: Author's manuscript [file bmjopen-2022-069135.draft_revisions.pdf]

BMJ Open is committed to open peer review. As part of this commitment we make the peer review history of every article we publish publicly available.

When an article is published we post the peer reviewers' comments and the authors' responses online. We also post the versions of the paper that were used during peer review. These are the versions that the peer review comments apply to.

The versions of the paper that follow are the versions that were submitted during the peer review process. They are not the versions of record or the final published versions. They should not be cited or distributed as the published version of this manuscript.

BMJ Open is an open access journal and the full, final, typeset and author-corrected version of record of the manuscript is available on our site with no access controls, subscription charges or pay-per-view fees (http://bmjopen.bmj.com).

If you have any questions on BMJ Open's open peer review process please email info.bmjopen@bmj.com

BMJ Open

Early recognition and management of maternal sepsis in Pakistan: A feasibility study of the implementation of FAST-M Intervention

Journal:	*BMJ Open*
Manuscript ID	bmjopen-2022-069135
Article Type:	Original research
Date Submitted by the Author:	13-Oct-2022
Complete List of Authors:	Ahmed, Sheikh; The Aga Khan University, Obs and Gynae<br>Rind , Ghulam Kubra; The Aga Khan University Hospital<br>Sikandar , Raheel ; Liaquat University of Medical and Health Sciences, OBGYN<br>Raza , Amir ; The Aga Khan University Hospital Main Campus Karachi<br>Khowaja, Bakhtawar; The Aga Khan University Hospital Main Campus Karachi<br>Parveen, Fahmida; Liaquat University of Medical and Health Sciences<br>Khan , Sehrish ; Liaquat University of Medical and Health Sciences<br>Memon, Nazia; Liaquat University of Medical and Health Sciences<br>Jahangir, Arshia ; The Aga Khan University<br>Mirza, Daayl ; Ziauddin University<br>Cheshire , James ; University of Birmingham<br>Dunlop, Catherine; University of Birmingham<br>Shakoor, Sadia; The Aga Khan University Hospital Main Campus Karachi<br>Barolia, Rubina; The Aga Khan University Hospital Main Campus Karachi<br>Sheikh, Lumaan; The Aga Khan University Hospital Main Campus Karachi<br>Saleem, Sarah; The Aga Khan University<br>Coomarasamy, Arri; University of Birmingham<br>Lissauer, David ; Malawi-Liverpool-Wellcome Trust Clinical Research Programme, ; University of Liverpool,
Keywords:	OBSTETRICS, INFECTIOUS DISEASES, Maternal medicine < OBSTETRICS

SCHOLARONE™
Manuscripts

I, the Submitting Author has the right to grant and does grant on behalf of all authors of the Work (as defined in the below author licence), an exclusive licence and/or a non-exclusive licence for contributions from authors who are: i) UK Crown employees; ii) where BMJ has agreed a CC-BY licence shall apply, and/or iii) in accordance with the terms applicable for US Federal Government officers or employees acting as part of their official duties; on a worldwide, perpetual, irrevocable, royalty-free basis to BMJ Publishing Group Ltd ("BMJ") its licensees and where the relevant Journal is co-owned by BMJ to the co-owners of the Journal, to publish the Work in this journal and any other BMJ products and to exploit all rights, as set out in our licence.

The Submitting Author accepts and understands that any supply made under these terms is made by BMJ to the Submitting Author unless you are acting as an employee on behalf of your employer or a postgraduate student of an affiliated institution which is paying any applicable article publishing charge ("APC") for Open Access articles. Where the Submitting Author wishes to make the Work available on an Open Access basis (and intends to pay the relevant APC), the terms of reuse of such Open Access shall be governed by a Creative Commons licence – details of these licences and which Creative Commons licence will apply to this Work are set out in our licence referred to above.

Other than as permitted in any relevant BMJ Author's Self Archiving Policies, I confirm this Work has not been accepted for publication elsewhere, is not being considered for publication elsewhere and does not duplicate material already published. I confirm all authors consent to publication of this Work and authorise the granting of this licence.

For peer review only

Paper Title:

Early recognition and management of maternal sepsis in Pakistan: A feasibility study of the implementation of FAST-M Intervention

Corresponding Author*

Sheikh Irfan Ahmed

Aga Khan University Hospital, National Stadium Road, Karachi city, Pakistan. P.O. Box 3500 Postal code: 74800

Email: sheikh.irfan@aku.edu

Author Names:

Sheikh Irfan Ahmed[1]

Ghulam Kubra Rind[1]

Raheel Sikandar[2]

Amir Raza[1]

Bakhtawar M.Hanif Khowaja[1]

Fahmida Parveen [2]

Sehrish Khan[2]

Nazia Memon [2]

Arshia Jahangir[3]

Daayl Naim Mirza[4]

James Cheshire[5]

Catherine Dunlop [5]

Sadia Shakoor[1]

Rubina Barolia[1]

Lumaan Sheikh[1]

Sarah Saleem[1]

Arri Coomarasamy[5]

David Lissauer[6, 7]

Affiliations & full institutional mailing addresses of all authors

[1] Aga Khan University Hospital, National Stadium Road, Karachi city, Pakistan. P.O. Box 3500 Postal code: 74800

[2] LUMHS Hospital Liaquat University of Health and Medical Sciences, Hyderabad city, Pakistan. Postal Code: 76090

[3] Aga Khan University, National Stadium Road, Karachi city, Pakistan. P.O. Box 3500 Postal code: 74800

[4] Ziauddin University4/B Shahrah-e-Ghalib Rd, Block 6 Clifton, Karachi, Karachi City, Sindh 75000

For peer review only

[5] Institute of Metabolism and Systems Research, University of Birmingham, Edgbaston, Birmingham, UK, B15 2TT.

[6] Institute of Life Course and Medical Sciences, University of Liverpool, Liverpool, William Henry Duncan Building, Liverpool, UK, L7 8TX.

[7] Malawi-Liverpool-Wellcome Clinical Research Programme, Chichiri, Blantyre 3, Malawi

Email addresses

sheikh.irfan@aku.edu

ghulam.kubra@aku.edu

raheel.sikandar@lumhs.edu.pk

amir.raza@aku.edu

bakhtawar.hanif@aku.edu

dr_memon16@yahoo.com

drkhanhyd@gmail.com

nazia.memon23@yahoo.com

arshia.jahangir@scholar.aku.edu

daayl.13094@zu.edu.pk

sadia.shakoor@aku.edu

james.cheshire@nhs.net

catherinedunlop@nhs.net

rubina.barolia@aku.edu

lumaan.sheikh@aku.edu

sarah.saleem@aku.edu

A.Coomarasamy@bham.ac.uk

David.Lissauer@liverpool.ac.uk

For peer review only

Word Count: 5148

ABSTRACT

Objective: Maternal sepsis is the 3rd leading cause of maternal mortality globally. WHO and collaborators developed a care bundle called FAST-M (**F**luids, **A**ntibiotics, **S**ource identification and treatment, **T**ransfer, and **M**onitoring) for early identification and management of maternal sepsis, particularly in low-resource settings. This study aimed to determine the feasibility of the FAST-M intervention in a low-resource setting in Pakistan. The FAST-M intervention consists of maternal sepsis screening tools, treatment bundle and the implementation program.

Design and setting: The FAST-M intervention was optimised for Pakistan, and a feasibility study with before and after design was conducted in women with suspected maternal sepsis admitted at the LUMHS hospital Hyderabad. The study outcomes were compared between the baseline and the intervention phase. In the baseline phase, the existing sepsis care practices of health care providers were recorded, followed by a training program for health care providers on the application of FAST-M tools. These tools were implemented in the intervention phase to assess any change in clinical practices compared to the baseline phase.

Results: Following the FAST-M intervention , statistically significant changes were observed when compared to the baseline practices. Improvements were seen in women with suspected sepsis in monitoring of oxygen saturation measurements [25.5% vs 100%; difference: 74%; 95% CI:68.4%-80.5% p<0.01], fetal heart rate assessment [58% vs100%; difference:42.0%; 95%CI: 33.7% to 50.3%; p=<0.01], and measurement of urine output [76.5% vs 100%; difference 23.5%; 95%CI: 17.6% to 29.4%; p<0.01]). Women with suspected maternal sepsis received all components of the FAST-M treatment bundle within one hour of sepsis recognition following the FAST-M intervention [0% vs70.5%; difference 70.5%; 95%CI: 60.4% to 80.6%; p<0.01].

Conclusion: Implementation of the FAST-M intervention was feasible and significantly improved the quality of maternal sepsis care, including enhanced early identification and management of maternal sepsis.

Trial registration number: ISRCTN17105658

For peer review only

Strengths and limitations:

- The strength of our research is that it's the first feasibility study conducted for the assessment of a maternal sepsis care bundle in the low-resource setting of Pakistan.
- The study design does not account for the temporal effects, hence is exposed to possible selection and reporting bias.
- This study was conducted at one site only i.e., LUMHS which is a tertiary hospital that was comparatively better equipped than basic health units (BHUs) to implement any care bundle approach.
- Since most of the patients who were referred from other facilities were administered fluid and antibiotics before admission to the hospital, which could have resulted in an overestimation of recording of fluid and antibiotics, that might have limited the ability of before and after comparison.

For peer review only

INTRODCUTION

Maternal sepsis is ‘a life-threatening condition defined as organ dysfunction resulting from infection during pregnancy, childbirth, post-abortion, or the post-partum period’.[1] Worldwide, it’s the 3rd most common cause of maternal mortality and represents around 11% of maternal deaths.[2] Pregnant and those that have recently delivered are more susceptible to maternal infections that can rapidly progress towards sepsis.[3,4]

The maternal mortality caused by maternal infections has been reported to be higher in Low- and Middle-income countries (LMICs) than High-Income countries (HICs) i.e., 10·7% in LMICS vs 4·7% in HICs.[2] In 2020, the WHO Global Maternal Sepsis Study (GLOSS) from hospital-based facilities of high and LMICs estimated that 70.4 hospitalized women per 1000 live births had a maternal infection, and 11 women per 1000 live births had infection-related adverse outcomes.[5]

Modelling studies, based on currently available data, observed that globally around 17000 maternal deaths per year may be attributed to maternal sepsis and other maternal infections (MSMI).[6] Approximately 5·7 million women suffered complications of maternal sepsis globally in 2017.[7] Annually, 99% of the 302,000 maternal deaths occurred in LMICs,[8] with the highest rates reported in Africa and Asia.[8] The reported maternal mortality ratio (MMR) in Pakistan is 186 per 100,000 live births in 2020,[9] and sepsis ranked as the 3rd leading cause of maternal deaths (16.3%)[10] following haemorrhage, and eclampsia.[2]

Improving maternal outcome is critical in Pakistan, which failed to achieve Millennium Development Goals (MDGs) 4 and 5.[11] Pakistan is now signatory to goal 3 of the Sustainable Development Goal (SDG) which aims to reduce maternal mortality to 70/100,000 by 2030.[12] Whilst the country shows significant improvements in some health indicators,[13] the rate of reduction in infant and maternal mortality is low to achieve these indicators in given time.[14]

For peer review only

Maternal sepsis has been recognized as a global health priority, and attempts were made to reduce its burden, particularly in LMICs.[15] In 2015, the Global maternal and neonatal sepsis initiative was introduced by the WHO and other partners to design strategies that could help in improving the recognition and management of maternal and neonatal sepsis in low-resource settings.[15,16] This initiative aimed to develop and test the effective strategies to prevent, detect, and manage the maternal and neonatal deaths caused by sepsis.[16] This included the key recommendation to develop a sepsis care bundle specific to maternal population.

Care bundles are a collection of evidence-based practices (usually three to five items), which when consistently combined in use, improve patient outcomes.[17] The use of these care bundles in the management of sepsis has been found to reduce mortality and improve patient outcomes in high-income countries.[18,19] Most notably, the surviving sepsis campaign (SSC) bundles,[20,21] as well as the UK Sepsis Trusts' Sepsis 6 bundle[22] have been widely used.[19] Despite this, there was no sepsis care bundle in particular for the maternal population which can be implemented efficiently in low-resource settings.[23-25]

To address this, a care bundle for treatment of maternal sepsis in low resource settings was developed after reaching the consensus utilising a modified Delphi approach by an international health expert panel.[26] The selected components were Fluids, Antibiotics, Source identification and control, Transfer to an appropriate level of care, and ongoing Monitoring of mother and neonate. The bundle was named “FAST-M” as a memorable acronym for supporting implementation and communication.[26] The FAST-M bundle was first implemented in 15 government healthcare facilities in Malawi, and it was not only found to be feasible to implement but also resulted in improved clinical care.[27]

Despite existence of national sepsis guidelines for Pakistan (SGP), their uptake remains low especially among obstetric patients.[28] Additionally, there is uncertainty that the existing evidence-based approach to prevent, recognise, and treat maternal sepsis is effective and implementable in a low-resource setting. It was, therefore, planned to determine the feasibility of the FAST-M intervention in the local setting before recommending its

For peer review only - http://bmjopen.bmj.com/site/about/guidelines.xhtml

implementation at scale. The study protocol and procedures have been described in detail and published elsewhere.[29]

To optimize the use of the FAST-M intervention in Pakistan, a robust process of adaptation was required before it could be field-tested in a local tertiary hospital. A qualitative study including key informant interviews and a focus group discussion was conducted with health care providers (HCPs) and administrators who were involved in sepsis management to understand the existing sepsis management practices/behaviours, and to identify potential gaps and resource availability in the local setting.[30] Based on the qualitative findings, the FAST-M bundle care tools which were used in Malawi[27] were modified to plan a practical approach for the implementation of the intervention in our local context. The results of the adaptation process have been described in a separate paper.[30] This study aimed to determine whether FAST-M intervention is feasible to introduce for the early detection and management of maternal sepsis in a low-resource setting in Pakistan.

METHODS

A feasibility study with a before and after design was conducted at the Liaquat University of Medical and Health Sciences, (LUMHS) Hyderabad, Pakistan. Women who were pregnant, or within 6 weeks of miscarriage, ectopic pregnancy, termination of pregnancy, or delivery were considered eligible for this study. Those admitted women who had abnormal maternal observations, or healthcare practitioner's concern regarding potential maternal infection, or pregnant women with a fetal tachycardia greater than or equal to 160 beats per minute were included in the study. There were no exclusion criteria for the study.

In the baseline phase, data were collected from facility record using CRF1 (facility audit form) to assess available resources for sepsis management and overall maternal outcomes in last six months. Data of patients who were meeting eligibility criteria were recorded on CRF 2 and CRF 3 to assess existing practices for management of maternal sepsis. Following this, the intervention phase was carried out. At first HCPs were trained on the use of FAST-M bundle care tools (MEOWS chart, decision tool and treatment bundle) to early

For peer review only - http://bmjopen.bmj.com/site/about/guidelines.xhtml

identify and manage sepsis patients. The HCPs training was an ongoing process throughout the implementation of intervention. The FAST-M bundle care tools were then implemented in intervention phase in the facility and data of all patients meeting the eligibility criteria were recorded using CRF 2 and CRF 3. The flow of study participants has been demonstrated in **(Figure 1).**

Comparisons were made between baseline and intervention phase to assess any change in monitoring and management of sepsis patients. Maternal outcomes were also compared between baseline and intervention phase.

Intervention:

The planned intervention consisted of 3 components:

1. Maternal sepsis screening tools
2. FAST-M treatment bundle
3. FAST-M implementation Programme

Component 1- Maternal sepsis screening tools

Maternal sepsis screening tools included the Modified Early Obstetric Warning Scores chart (MEOWS) and the FAST-M decision tool **(Supplemental File 1**).MEOWS is the tool adapted by Malawi study based on an early warning score approach and simplified from the approach adopted in UK hospitals.[31] The tool assisted in charting an individual's physiological parameters over time with guiding thresholds that signal clinical action if they become abnormal.[32]

The MEOWS chart used in FAST-M implementation in the districts of Malawi [27] was adapted in the context of Pakistan for this study. During the adaptation phase, the health care practitioners recommended the inclusion of oxygen saturation into the MEOWs chart to determine patient's clinical condition considering the outbreak of COVID-19 infection and the availability of pulse oximeters at the study site. Therefore, the oxygen saturation component was incorporated in the modified MEOWS chart based on the scope of existing practices and the significance of this indicator in the identification of patient's clinical condition.

For peer review only

Abnormal observations (indicated by a single red or two yellow flags) triggered a review by a clinician. These patients were then screened for potential sepsis using the FAST-M decision tool. The patients who developed a red trigger on the MEOWS chart received an immediate clinical review, and a treatment bundle was initiated within an hour. Those patients who triggered two yellow flags on the MEOWS chart and were thought to have possible infection were reviewed within three hours. All suspected cases (those without red or two yellow triggers, were still considered for having a risk of sepsis and remained under observation for possible development of red or yellow triggers), these patients were monitored on the MEOWS chart hourly throughout the day and were managed as per local guidelines.

Component 2-FAST-M Treatment Bundle

The FAST-M treatment bundle consists of Fluids, Antibiotics, Source identification and Control, Assessment of the need to Transport / Transfer to a high level of care, and Ongoing Monitoring (of the mother and neonate). The first and second-line antibiotic therapy guidelines were modified as per the local context during the adaptation phase **(Supplemental File 1)**. Patients with suspected sepsis were commenced immediately on the FAST-M treatment bundle. Each patient's treatment was recorded on the treatment bundle, along with any actions taken and any reasons for not completing certain elements.

Component 3-FAST-M implementation Programme

The implementation program **(Figure 2)** consisted of the FAST-M training, selection of clinical champions, and feedback achieved from data collected during the baseline phase, and through the facility audit form.

The two-day training sessions were delivered by health care experts after the completion of the baseline phase, and before the implementation of the FAST-M bundle intervention. The training session started with classroom-based instruction on the FAST-M intervention followed by interactive scenarios used as a strategy for hands-on training of healthcare providers on the FAST-M tools. Follow-up training sessions were also conducted every 2 weeks by the local team leads during the implementation of the FAST-M bundle intervention.

For peer review only - http://bmjopen.bmj.com/site/about/guidelines.xhtml

All the health care practitioners working in obstetrics and gynecology units of LUMHS hospital were trained to record patients' vital signs on the MEOWS chart (heart rate, respiratory rate, oxygen saturation, blood pressure, conscious level, urine output, temperature, and fetal heart rate (if applicable). These observations were then charted on a MEOWS chart in the inpatient setting and then inserted into the patient's files.

The local clinical champions and team leaders were identified during training programs and trained to take the lead in implementation of the FAST-M intervention at the study site and remained engaged throughout the implementation process. These champions were selected from different hospital departments primarily involved in the management of maternal sepsis patient including obstetrics and gynecology units, emergency department, internal medicine, and intensive care unit. The selected champions included doctors and nurses.

The overarching goal of each champion was to encourage engagement and compliance with the FAST-M bundle. To achieve this goal, champions at each unit were engaged in several key activities that included disseminating knowledge, advocating, navigating boundaries, facilitating consensus, arranging meetings with stakeholders, tracking quality indicators, and developing organizational communication strategies and relationships. The patient records collected were also counter-checked by the clinical champions of the assigned units. These champions also helped the study team in the identification of patients with suspected sepsis and their enrolment in the study.

In addition, training certificates were distributed among the participants. Moreover, FAST-M tools (MEOWS chart, decision, and treatment tool) were displayed in the form of posters throughout all of the units involved in the management of maternal sepsis and implementation of FAST-M intervention.

Study Period:

The study comprised a baseline period of two months in which usual practices to detect and treat maternal sepsis existing locally were assessed. The FAST-M training was conducted on the completion of the baseline

For peer review only

period, following training, an intervention period of four months was commenced to assess any change in practice.

Outcomes:

Primary process outcomes included

1) The proportion of patients admitted with indications of sepsis, who received appropriate monitoring (full set of vital sign measurements on admission recorded on MEOWS chart).

2) The proportion of women with suspected maternal sepsis receiving the FAST-M treatment bundle (including each bundle component) within 1 hour of identification of sepsis.

Secondary outcomes included

1) Rate of vital signs with complete recording of MEOWS chart components [heart rate, respiratory rate, oxygen saturation, blood pressure, mental state, urine output, temperature, and fetal heart rate (in pregnant women)].

2) Rate of application of FAST-M Bundle components [Fluids, Antibiotics, source identification (identified clinically) and control, assessment of the need to transport/ transfer to a high level of care, and ongoing monitoring (of the mother and neonate)].

3) Rate of maternal morbidity and mortality after FAST-M intervention.

Data Collection:

Quantitative data for this feasibility study was collected by a senior research assistant (SRA) from facility record CRF 1 **(Supplement File 2)**, and patient notes including the MEOWS charts, FAST-M decision, and treatment tools onto the relevant paper-based CRFs 2 & 3 **(Supplemental File 3-4)** following patient consent **(Supplemental File 5)**. Data were composed of various outcomes; structural, clinical, organization, and any adverse events. The data were maintained in an investigator file to be secured in a locked cabinet. Anonymised and deidentified patients' information recorded on the data collection sheet were recorded in a database located on a secure server.

For peer review only

During the study, biweekly monitoring of the study site was undertaken by the research coordinator, to observe practice, collect feedback and provide updates on the study site's performance, and evaluate any additional training requirements.

Statistical Analysis:

Statistical Package for Social Sciences (SPSS) version 19.0 (IBM Corporation, Armonk, NY, USA) and STATA 14 computer software (Stata Corporation, Texas USA) was used for data processing and statistical analysis. Data were reported in frequency and percentages for categorical observations. A Chi-square test for independence was used to compare components of FAST-M bundle between phases (baseline and intervention). The Chi-square test for trend (P trend) was used to observe the performance during the intervention phase. Statistical significance was determined at $P \leq 0.05$.

Patient and public involvement:

Patients were not involved in the design of this study. The FAST-M intervention was implemented on the enrolled patients however, they were not invited to comment on the study design, interpret the results or contribute to writing or editing of this document for accuracy of findings. Hospital staff participated in the implementation but were not involved in the analysis of findings.

RESULTS:

Vital signs monitoring assessment: During the FAST-M implementation, 439 women were included in the study (200 in baseline and 239 in the intervention phase), with vital signs recorded on admission ***(Table 1)***. 242/439 were cases of suspected maternal infection, and 138/242 were women with suspected of maternal sepsis. Figure 1 provides the summary of the screening, and enrolment of the study participants. Following the FAST-M implementation, significant improvements were seen in the assessment of oxygen saturations in the intervention phase compared to the baseline phase [Difference: 74%; 95%CI:68.4%-80.5% p<0.01], fetal heart rate assessment [Difference:42.02%; 95%CI: 33.7% to 50.28%; p=<0.01], measurement of urine output [Difference 23.5%; 95%CI: 17.6% to 29.4%; p<0.01]. Temperature, respiratory rate, heart rate, and blood

For peer review only

pressure were measured in 97% of the cases in the baseline phase but showed an increase of almost 3% in the intervention phase [Difference 2.5% 95%CI: 0.34% to 4.66%; p≤0.05]. No significant difference was found in the assessment of mental status in the intervention phase when compared to the baseline phase [Difference 1.6%; 95%CI: -0.52% to 3.7%; p=0.18] **(Supplemental File 6)**.

Table 1: Patient's assessment of vital signs on admission (n=439)

Vital Sign	Baseline n=200	Intervention n=239	P-Value
Respiratory rate	194(97%)	239(100%)	0.009
Oxygen Saturations	51(25.5%)	239(100%)	0.0005
Temperature	195(97.5%)	239(100%)	0.019
Heart rate	194(97%)	238(99.6%)	0.051
Systolic BP	196(98%)	239(100%)	0.042
Diastolic BP	196(98%)	239(100%)	0.042
Urine output	153(76.5%)	239(100%)	0.0005
Mental State	196(98%)	238(99.6%)	0.182
Fetal heart rate (in pregnant woman) *	80 (58%)	123 (100%)	0.0005

*For FHR the denominator is 138 in baseline and 123 in the intervention phase.

Baseline characteristics of women with suspected maternal sepsis: 242 women were identified by the FAST-M decision tool as fulfilling the criteria for suspected maternal infection, of which 65% (130/200) women were during the baseline and 46.9% (112/239) during the intervention phase. Out of 242 women with maternal infections, 138 (57%) were identified with suspected maternal sepsis. Sixty of 138 (46.2%) were during the baseline phase and 78 of 112 (69.6%) were observed during the intervention phase.

The characteristics of the patients with suspected maternal sepsis are presented in **(Supplemental File 7)**. Fifty percent of women were of gestational age >28 weeks and 46% were in the post-natal period. Blood pressure (62.3%), respiratory rate (59.4%), and heart rate (50.7%) were the most common abnormal trigger followed by temperature (43%), appearance (41%), mental status (27%), and urine output (6%). The most common source of infection found was respiratory (31%), chorioamnionitis (15%), and urinary tract (11%).

For peer review only - http://bmjopen.bmj.com/site/about/guidelines.xhtml

Supplemental File 8 represents the various laboratory investigations carried out for the identification of source of infection in suspected maternal sepsis patients.

Compliance with the FAST-M treatment bundle within 1 hour: Women with suspected maternal sepsis received all the components of the FAST-M treatment bundle, with the goal of treatment within 1 hour. Compliance improved following the intervention, as compared to the baseline [70.5%; 95%CI: 60.4% to 80.6%; p<0.01] ***(Table 2)***. Improvements in management of sepsis cases were seen in all FAST-M bundle components, women were more likely to get IV fluids [52.2%; 95%CI: 38% to 66.3% ; p<0.01], antibiotics [24.5%; 95%CI: 12.3% to 36.6% ;p<0.001], source identification [27.8%; 95%CI: 15.3% to 40.3%; p<0.01], assessment of need to transfer required to high level care [65.3%; 95%CI: 52.8% to 77.6% ;p<0.01], and ongoing monitoring [63.3% ; 95%CI: 50.3% to 76.4%; p<0.01] within an hour of suspected sepsis identification compared to the baseline phase **(Figure 3)**. The improvements in the management of women with suspected maternal sepsis were maintained throughout the intervention, with no significant decline in performance over time (P trend value >0.05) **(Figure 4)**.

Table 2: Completion of FAST-M bundle within 1 hour of recognition of maternal sepsis

	**Baseline (n=60)**	**Intervention FAST-M (n=78)**	**Intervention over time**				**P-Value**	**P-trend**
			Month 1 (n=29)	Month 2 (n=18)	Month 3 (n=17)	Month 4 (n=14)		
**Total Bundle**	**0(0%)**	**55(70.5%)**	**20(69%)**	**9(50%)**	**15(88.2%)**	**11(78.6%)**	**0.0005**	**0.2069**
Fluids	21(35%)	68(87.2%)	24(82.8%)	17(94.4%)	15(88.2%)	12(85.7%)	0.0005	0.7516
Antibiotics	43(71.7%)	75(96.2%)	27(93.1%)	18(100%)	16(94.1%)	14(100%)	0.0001	0.3979
Source identification	41(68.3%)	75(96.2%)	28(96.6%)	17(94.4%)	17(100%)	13(92.9%)	0.0005	0.8405
Assessment of need to transfer high level	7(11.7%)	60(76.9%)	22(75.9%)	10(55.6%)	16(94.1%)	12(85.7%)	0.0005	0.1739
Monitoring	12(20%)	65(83.3%)	24(82.8%)	10(55.6%)	17(100%)	14(100%)	0.0005	0.0384

For peer review only

Maternal Outcomes: The facility data shows that the maternal mortality rate from the period of January to December 2021 was 279/24000 (1.1%.) **(Supplemental File 9)**. Out of these 279 deaths, 80 (28.6%) were due to maternal sepsis. A 9.4% decline in the sepsis related maternal mortality was observed: 52/159 (32.7%) in the month of January-June and 28/120 (23.3%) during the July-December 2021 (p-value=0.087).

DISCUSSION

Main Findings:

The implementation of the FAST-M intervention was feasible, and effective in early identification, and management of maternal sepsis at the study setting. There was high compliance with all monitoring components after the intervention, with significant increase in compliance with completion of the MEOWS charts. Following the intervention, women with suspected sepsis were more likely to receive all of the critical early interventions needed to treat maternal sepsis. There was high and sustained compliance during the post-interventional observation period with completion of the FAST-M treatment bundle within an hour once maternal sepsis was suspected.

Interpretation:

The use of the MEOWS chart has been related to improved health outcomes and has been shown to predict severe maternal morbidity and mortality. Though there is limited evidence on its feasibility and impact on outcomes in low resource settings.[33]The present study confirms the applicability of the MEOWS chart in a low resource setting in Pakistan. However, this chart can only be useful if a comprehensive set of observations is collected consistently. Any missing or irregular observations decreases the ability to identify the patients at risk.[34] In the present study, we found that with the combined use of the MEOWS chart along with health care provider training, resulted in improvement in the monitoring of vital signs after the implementation of the FAST-M intervention.

In low-resource settings, the lack of screening processes and resources can be an obstacle to the early detection and treatment of maternal sepsis.[35,36] Use of sepsis screening tools have been suggested to

For peer review only

decrease the time required to start treatment.[37,38] It was observed, that whilst the Royal College of Obstetricians and Gynaecologist guidelines (RCOG) were recommended for patient management and treatment at the LUMHS hospital, there was no standardised chart for the recording of patients' vital signs. Instead, the vital signs were directly recorded into the patient's file. The visual representation of the vital signs on the MEOWS chart aids in the recognition of abnormal vitals.

An earlier study in Malawi on FAST-M intervention showed that the use of the MEOWS chart along with staff training led to the improvement in vital signs monitoring.[27] The MEOWS chart used in the Malawian settings,[27] was adapted before its implementation in Pakistan based on health care providers' suggestions during the adaptation phase of the study. Consequently, the health care providers were trained on an adapted MEOWS chart for regular recording a full set of vital signs to screen patients who are at risk of developing sepsis ,including the recording of oxygen saturations. Providers showed a large improvement in the reliability of routine oxygen saturation recording following the intervention, so this component was shown to be a feasible addition in the Pakistan context.

Early detection of sepsis patients is critical for prompt treatment and improved maternal outcomes.[3,38,39] Research indicates that early warning scores accompanied by a clear escalation policy guide the healthcare providers on the need for additional action to optimise patient care.[40] The decision tool used guided the health care providers on when to proceed with treating the patient as having suspected sepsis and initiating the FAST-M bundle.[41,42] As noted earlier, the FAST-M screening tools in Malawi facilitated the identification of maternal sepsis patients and guided health care staff to escalate management plans. Patients were more likely to receive a clinical review by the senior decision-maker based on the diagnosis of suspected maternal sepsis.[27] The present study shows that it was also true in the Pakistan context where the FAST-M decision tool along with the MEOWS chart guided health care providers in appropriate screening and identification of women with suspected sepsis and assisted in making timely decisions for such cases.

For peer review only

The study was not powered to demonstrate reductions in maternal mortality or severe morbidity, but due to the critical importance of this outcome it was pre-specified as a secondary outcome. Following intervention, we observed a 9.4% decline in maternal mortality from facility audit survey (CRF1), which suggests that the early identification of the sepsis patients and their timely management may as hoped lead to improved clinical outcomes.

This finding is consistent with evidence from high income countries where the use of sepsis care bundles for the immediate treatment of sepsis has been demonstrated in large observational and interventional studies to reduce mortality and improve patient's condition.[18,19] It's been reported that each part of the care bundle should be completed to reach the desired result, and failure to complete any of the components may have an impact on the overall patient outcome.[40] In Malawi, the FAST-M bundle implementation was shown to improve the screening, diagnosis and management of maternal sepsis,[27] but it did not in that study demonstrate a reduction in maternal mortality, so our mortality reduction finding in Pakistan is particularly important.

Fluid administration is considered an important part of early sepsis management.[26,43] We observed a significant improvement in timely fluid administration once the FAST-M bundle was implemented. The restoration of organ perfusion is an important goal of maternal sepsis treatment and fluid resuscitation should seek to restore organ perfusion while maintaining hemodynamic stability.[40] However, the benefits and risks of intensive early goal-guided fluid resuscitation have been the subject of intensive research.[44-49] The bundle care tools we used guided health care providers with a recommended fluid administration approach and to cautiously reassess the patient's hemodynamic condition between fluid boluses, through clinical examination and monitoring of the patient's vital signs. This sought to avoid overloading the patient which can actually worsen sepsis outcomes.[50,51]

Fluid management in patients with maternal sepsis and pre-eclampsia is challenging due to the increased risk of pulmonary oedema and must therefore be particularly cautiously managed by healthcare professionals.[40]

For peer review only - http://bmjopen.bmj.com/site/about/guidelines.xhtml

During the FAST-M implementation, clear teaching and guidance were provided to the health care providers for proper fluid administration in pre-eclamptic or eclamptic patients.

Antibiotics are considered the cornerstone of sepsis treatment, and studies have shown that timely administration of antibiotics improves patient outcomes and reduces mortality.[39,52] The risk of death from sepsis increases with each hour of antibiotic delay (OR 1.04 per hour; 95 percent CI 1.03–1.06; p 0.001).[39] As a result, empiric broad-spectrum antibiotics should be given as early as possible, and it is normally suggested that clinicians should aim to provide this where possible within 1 hour of sepsis detection.[53,54]

It's expected that the 24.5 percent increase in the present study in timely initiation of antibiotic administration may have led to the improved clinical condition of women with suspected maternal sepsis. In Malawi, 42.3% improvement was seen in intravenous antibiotics administration within an hour of sepsis detection, from a relatively low baseline performance of 25%.[27] In LUMHS hospital, we modified the recommendations for antibiotic therapy before implementation of the FAST-M treatment bundle intervention. Ceftriaxone was selected as the first line of antibiotic therapy, due to its low cost, and availability in the facility. Piperacillin/tazobactam and meropenem were included as second line therapy in the treatment bundle due to high levels of antibiotic resistance in this population, making these the health care providers preference and they were available in this context **(Supplemental File 1)**.

Source identification was improved by 27.8% after FAST-M implementation in the study setting. Similar findings were reported in Malawi, where source identification improved by 18.2% within an hour of sepsis detection after FAST-M intervention.[27] Research suggests that the patient's history along with the complete clinical examination is often sufficient for the identification of the source of infection.[40] If the source is not immediately clear, further investigations are required.[40]

In the baseline phase, it was observed that clinicians had reservations to advise additional tests to identify the source of infection due to the logistics of undertaking these investigations in the facility and potential

For peer review only

additional costs to be incurred by the patients. Considering this, the health care providers were given guidance during the training sessions regarding the significance of further investigations if the source is not initially clear with the clinical history and examination. Consequently, an increase was seen in measurements of blood culture, imaging (abdominal, and chest), urine and other blood tests in the intervention phase **(Supplemental File 8)**.

Amongst patients receiving treatment for maternal sepsis, the need for transfer to a higher level of care should be considered, and the decision on a patient's transfer made by a senior clinician as part of their review.[40] In Malawi, 43.9% improvement was seen in the consideration of transfer to the high level of care after the FAST-M intervention implementation.[27] It was observed that LUMHS being the only tertiary referral public sector hospital in the area, its high dependency units (HDUs) and intensive care units (ICUs) were often fully occupied due to the high patient numbers and their needs. This led to the delay in transfer of women with maternal sepsis to higher levels of care. During the training program, the importance of assessment of a patient for transfer was highlighted, and emphasis was made on expediting this process to ensure it was completed within an hour of sepsis detection if their vital signs were not improving after initial sepsis treatment. Following this, the assessment of need to transfer was improved by 65.3% within an hour of sepsis detection in the intervention period.

Overall, following the FAST-M intervention, women were more monitored and screened for maternal sepsis, and the complete FAST-M treatment bundle was administered within an hour in 70.5% of women with suspected sepsis. This resulted in improvement in the clinical outcomes of women with suspected maternal sepsis. The study findings demonstrate that the FAST-M intervention was feasible and improved the quality of care and clinical outcomes when implemented in the context of a large government hospital in Pakistan. FAST-M has the potential to be used as an integrated approach for early recognition and management of maternal sepsis in low-resource health settings of Pakistan and other LMIC's. However, as we found when translating the FAST-M tools from Malawi to Pakistan some modifications in the intervention were needed to ensure they were optimised for the local context and available resources.

For peer review only

Strengths and limitations:

The strength of our research is that it's the first feasibility study conducted for the assessment of a maternal sepsis care bundle in the low-resource setting of Pakistan. This approach provided the opportunity to analyse practical, and operational factors for introducing the FAST-M intervention in the local setting. The findings of this study can lead to optimization of the bundle prior to the large-scale intervention trial. Also, this study adds further weight to Malawi work demonstrating the feasibility of the FAST-M bundle in different low resource settings.

However, the study has several limitations. Our research design does not account for the temporal effects, hence is exposed to possible selection and reporting bias.[55] Although a before and after design couldn't identify the cause-and-effect relationship, this was the practical approach for determining the feasibility of the FAST-M intervention locally.

This study was conducted at one site only i.e., LUMHS which is a tertiary hospital that was comparatively better equipped than basic health units (BHUs) to implement any care bundle approach. BHUs operate at the district level as the first level of a healthcare facility in the primary healthcare structure and serve around 5,000-10,000 people.[56] Hence, the intervention will next be implemented in other low-resource settings including BHU's across Pakistan. A large multi-country randomised trial is also underway to more robustly ascertain the effectiveness of the FAST-M bundle to improve maternal sepsis care and outcomes in LMICs.

Although the intervention was found to be feasible, we recognise that the intervention period was short. A further assessment period is required to examine how well the FAST-M intervention is sustained and if it becomes normalised as a routine part of clinical care in the longer term. It is also important to understand in detail the process of implementation and fully understand the acceptability, and barriers and facilitators to implementation and how this was influenced by the context. This additional information has been collected via an embedded qualitative study and will be reported separately.

For peer review only

Additionally, LUMHS is a tertiary public sector hospital that serves a large number of underprivileged populations referred from primary and secondary care settings such as private clinics, rural health centres (RHCs), and BHUs. The high influx of patients and shortage of health care staff could have led to irregular monitoring which might have resulted in limited screening of the patients. Some of the women with suspected sepsis probably categorised as maternal infection cases, that might have restricted the ability to demonstrate the differences between intervention and baseline phase.

Since most of the patients who were referred from other facilities were administered fluid and antibiotics before admission to the hospital, which could have resulted in an overestimation of recording of fluid and antibiotics, that might have limited the ability of before and after comparison. Additionally, the health care providers might have been more compliant with the FAST-M intervention, and the outcomes of this study could have been affected due to the close monitoring of the study site which is possibly defined by the Hawthorn effect.

CONCLUSION

FAST-M implementation was feasible in the low-resource setting of Pakistan. The intervention improved vital sign monitoring and assisted in the early detection and management of maternal sepsis cases, resulting in improved clinical outcomes for women with suspected sepsis. The long-term vision is that the intervention will be trialled in other settings across Pakistan, based on the outcomes of this feasibility study, which will help to reduce the high rate of maternal deaths caused by sepsis in the country.

Acknowledgments: We would like to acknowledge the healthcare providers from the LUMHS hospital Dr Prince Akash, Dr Aqsa, Dr Batool, Dr Sana, Dr Qurat ul ain, Dr. Sahiba, and staff nurse Tomsina who served as FAST-M champions and actively took on their role in implementing the FAST-M bundle intervention. We would also like to acknowledge Ms. Ruqaiya Bano for data collection of this study and Ms Maliha Fazal for editing the manuscript.

For peer review only

Contributors: Contributors: DL and SI designed the study and its processes. AR, SI GK, AJ and DM carried out the data analysis and interpretation. SI, GK, AR, and BK took the lead in writing the manuscript. SI takes responsibility for the overall content as the guarantor. AC, DL, CD, JC, LS, RS, RB, SS, FP reviewed the manuscript and provided feedback on the article.

Funding: This research was commissioned by the National Institute for Health Research (NIHR). Award Ref: NIHR300808 Host: University of Liverpool. The views expressed in this paper are those of the authors and not necessarily those of the NIHR or the Department of Health and Social Care. The project was also supported by PhD award of WHO Eastern Mediterranean Hub (AKU) of HRP Alliance, for Research Capacity Development in Sexual and Reproductive Health, University of Birmingham Alumni funds, and Aga Khan University Research Funds for Faculty.

Competing interests: Declared none.

Patient and public involvement: Patients were not involved in planning the design of this study. The FAST-M intervention was implemented on the enrolled patients however, they were not invited to comment on the study design, interpret the results or contribute to writing or editing of this document for accuracy of study findings. Hospital staff participated in the implementation but were not involved in the analysis of findings for accuracy and readability.

Patient consent for publication: Not applicable.

Ethics approval: Ethical approval for this study was obtained from the LUMHS hospital [REC/-886, 4-87], Aga Khan University Ethical Review Committee [2019-2061-7102], and National Bioethics Committee [515/20/].

Provenance and peer review: Not commissioned; externally peer reviewed.

Data availability statement: The datasets were collected and analyzed and can be made available from the corresponding author on reasonable request.

For peer review only

ORCID iDs:

SI: 0000-0002-8391-8559

GK: 0000-0003-3224-730X

RS: 0000-0002-8015-5744

AR: 0000-0003-3970-8236

BK: 0000-0002-8141-9203

FP: 0000-0002-7820-6565

SK: 0000-0002-9655-378X

NM: 0000-0002-5174-8671

AJ: 0000-0002-6674-8543

DM:0000-0002-3396-3087

SS: 0000-0003-1301-5239

JC: 0000-0002-0967-7320

CD: 0000-0002-4792-9496

RB: 0000-0002-7377-9524

LS: 0000-0002-7694-3561

SS: 0000-0002-6797-8631

AC: 0000-0002-3261-9807

DL: 0000-0002-7878-232

For peer review only

References:

1. Bonet M, Pileggi V, Rijken MJ, Coomarasamy A, et al. Towards a consensus definition of maternal sepsis: results of a systematic review and expert consultation. *Reprod Health* 2017;14:67.
2. Say L, Chou D, Gemmill A, et al. Global causes of maternal death: a WHO systematic analysis. *Lancet Global Health*. 2014 Jun 1;2(6):e323-33.
3. Kourtis AP, Read JS, Jamieson DJ. Pregnancy and infection. *N Engl J Med*. 2014;370(23):2211–8.
4. Vasudevan R. Urinary tract infection: an overview of the infection and the associated risk factors. *J Microbiol Exp*. 2014;1(2):42–54.
5. Bonet M, Brizuela V, Abalos E, et al. Frequency and management of maternal infection in health facilities in 52 countries (GLOSS): a 1-week inception cohort study. *Lancet Global Health.* 2020 May 1;8(5):e661-71
6. Vos T, Lim SS, Abbafati C, et al. Global burden of 369 diseases and injuries in 204 countries and territories, 1990–2019: a systematic analysis for the Global Burden 2019. *Lancet*. 2020;396(10258):1204–22
7. Rudd KE, Johnson SC, Agesa KM, et al. Global, regional, and national sepsis incidence and mortality, 1990–2017: analysis for the Global Burden of Disease Study. *Lancet.*2020; 395: 200–11.
8. World Health Organization. Trends in maternal mortality: 1990 to 2015: estimates from WHO, UNICEF, UNFPA, World Bank Group and the United Nations Population Division: executive summary. (apps.who.int/iris/handle/10665/193994) Accessed on 1st Sept 2022
9. Hanif M, Khalid S, Rasul A, et al. Maternal mortality in rural areas of Pakistan: challenges and prospects. *Rural Heal*. 2021 Jun 7;27:1040-7.
10. Fikree FF, Ali T, Durocher JM, et al. Health service utilization for perceived postpartum morbidity among poor women living in Karachi. *Social science & medicine*. 2004 Aug 1;59(4):681-94.
11. National Institute of Population Studies (NIPS), ICF International (2013) Pakistan Demographic and Health Survey 2012-13. NIPS and ICF International, Islamabad and Calverton. - References - Scientific Research Publishing (www.scirp.org/(S(351jmbntvnsjt1aadkposzje))/reference/referencespapers.aspx?referenceid=2849234) Accessed on 1st Sept 2022
12. Boldosser-Boesch A, Brun M, Carvajal L, et al. Setting maternal mortality targets for the SDGs. *Lancet*. 2017;389(10070):696-697. doi: 10.1016/S0140-6736(17)30337-9
13. Omer S, Zakar R, Zakar MZ, et al. The influence of social and cultural practices on maternal mortality: a qualitative study from South Punjab, Pakistan. *Reproductive health*. 2021 Dec;18(1):1-2.
14. Rashid F, Makhdoom S. Maternal health in Pakistan: where do we stand? *J Islamabad Med Dental College*. 2019;8(3):99–100.
15. Reinhart K, Daniels R, Kissoon N, et al. Recognizing sepsis as a global health priority – A WHO resolution. *N Engl J Med* 2017;377:414–7.
16. World Health Organization. Statement on Maternal Sepsis. 2017. World Health Organization. [apps.who.int/iris/handle/10665/254608] Accessed 1 September 2022.
17. Institute for Healthcare Improvement. Evidence-Based Care Bundles IHI. [www.ihi.org/Topics/Bundles/Pages/default.aspx]. Accessed 1 September 2022.
18. Damiani E, Donati A, Serafini G, et al. Effect of performance improvement programs on compliance with sepsis bundles and mortality: a systematic review and meta-analysis of observational studies. *PloS one*. 2015 May 6;10(5):e0125827.
19. Levy MM, Dellinger RP, Townsend SR, et al. The Surviving Sepsis Campaign: results of an international guideline-based performance improvement program targeting severe sepsis. *Crit Care Med* 2010;38(2):367–74.
20. Levy MM, Evans LE, Rhodes A. The Surviving Sepsis Campaign Bundle: 2018 update.*Int Care Med* 2018;1–4.
21. Evans L, Rhodes A, Alhazzani W, et al. Surviving sepsis campaign: international guidelines for management of sepsis and septic shock 2021. *Int care med*. 2021 Nov;47(11):1181-247.
22. Nutbeam T, Daniels R, Keep J, UK Sepsis Trust. Toolkit: Emergency Department management of Sepsis in adults and young people over 12 years- 2016. The Royal College of Emergency Medicine; National Institute for Health and Care Excellence (NICE); 2016.
23. Abdu M, Wilson A, Mhango C, et al. Resource availability for the management of maternal sepsis in Malawi, other low-income countries, and lower-middle-income countries. *Int J Gynecol Obstet* 2018;140:175–83.
24. Baelani I, Jochberger S, Laimer T, et al. Availability of critical care resources to treat patients with severe sepsis or septic shock in Africa: a self-reported, continent wide survey of anaesthesia providers. *Crit Care* 2011;15:R10.
25. Bataar O, Lundeg G, Tsenddorj G, et al. Nationwide survey on resource availability for implementing current sepsis guidelines in Mongolia. *Bull World Health Organ* 2010;88:839–46.
26. Lissauer D, Cheshire J, Dunlop C, et al. Development of the FAST-M maternal sepsis bundle for use in low-resource settings: a modified Delphi process. *BJOG-INT J OBS*. 2020 Feb;127(3):416-23
27. Cheshire J, Jones L, Munthali L, et al. The FAST-M complex intervention for the detection and management of maternal sepsis in low-resource settings: a multi-site evaluation. *BJOG-INT J OBS*. 2021 Feb 4.
28. Reinhart K, Kissoon N. Sepsis Guidelines for Pakistan. *Anaesth Pain & intensive Care*. 2015 Apr 1;19(2):105-7.
29. Ahmed SI, Sikandar R, Barolia R, et al.Evaluation of the feasibility of the FAST-M maternal sepsis intervention in Pakistan: a protocol. *Pilot and feasibility studies*. 2022 Dec;8(1):1-2.
30. Ahmed SI, Khowaja BMH, Barolia R, et al. Adapting the FAST-M maternal sepsis intervention for implementation in Pakistan: a qualitative exploratory study. *BMJ Open* 2022;12:e059273.
31. Cheshire J. Fast-m study protocol. 2017 *Trial website* [https://www.birmingham.ac.uk/research/activity/fast-m/index.aspx] Accessed 19 September 2022.
32. National Early Warning Score (NEWS) - RCP London [https://www.rcplondon.ac.uk/file/32/download] Accessed 1 September 2022.

For peer review only - http://bmjopen.bmj.com/site/about/guidelines.xhtml

33. Umar A, Ameh CA, Muriithi F, et al. Early warning systems in obstetrics: a systematic literature review. *PLoS One* 2019;14: e0217864.
34. Downey CL, Tahir W, Randell R, et al. Strengths and limitations of early warning scores: A systematic review and narrative synthesis.*I nt J Nurs Stud* 2017;76:106–19.
35. Pakenham-Walsh N, Bukachi F. Information needs of health care workers in developing countries: a literature review with a focus on Africa. *Hum Resour Health* 2009;7:30.
36. Papali A, McCurdy MT, Calvello EJB. A 'three delays' model for severe sepsis in resource-limited countries. *J Crit Care* 2015;30:861.e9– 861.e14.
37. Gatewood MO, Wemple M, Greco S, et al. A quality improvement project to improve early sepsis care in the emergency department. *BMJ Qual Saf* 2015;24:787–95.
38. Hayden GE, Tuuri RE, Scott R, et al. Triage sepsis alert and sepsis protocol lower times to fluids and antibiotics in the ED. *Am J Emerg Med* 2016;34:1–9.
39. Seymour CW, Gesten F, Prescott HC, et al. Time to treatment and mortality during mandated emergency care for sepsis. *N Engl J Med* 2017;376:2235–44.
40. Cheshire J, Dunlop C, et al, Glob. libr. women's med., ISSN: 1756-2228; DOI 10.3843/GLOWM.413113
41. The Uk Sepsis Trust. Clinical Professional Resources. 2018.
42. Suleiman I, Vousden N, Shennan AH. Recognition and Management of Maternal Sepsis in Low and Middle-Income Countries: What do we know and where are the Gaps? Open access. *Glob Womens Heal* 2018;1(1):1–5.
43. Angus, DC, van der Poll, T. Severe sepsis and septic shock. *N Engl J Med*. 2013;369(9):840–851.
44. Rivers E, Nguyen B, Havstad S, et al. Early Goal-Directed Therapy in the Treatment of Severe Sepsis and Septic Shock.*N Engl J Med* 2001;345(19):1368–77.
45. Mouncey PR, Osborn TM, Power GS, et al. Trial of Early, Goal-Directed Resuscitation for Septic Shock. *N Engl J Med* 2015;372(14):1301–11.
46. RISE Investigators, ANZICS Clinical Trials Group, Peake SL, Delaney A, Bailey M, Bellomo R,e t al. Goal-Directed Resuscitation for Patients with Early Septic Shock. *N Engl J Med* 2014;371(16):1496–506.
47. Angus DC, Yealy DM, Kellum JA. Protocol-based care for early septic shock. *N Engl J Med*. 2014 Jul 1;371(4):386-.
48. Jaehne AK, Rivers EP. Early Liberal Fluid Therapy for Sepsis Patients Is Not Harmful: Hydrophobia Is Unwarranted but Drink Responsibly. *Crit Care Med* 2016;44(12):2263–9.
49. Andrews B, Semler MW, Muchemwa L, et al. Effect of an Early Resuscitation Protocol on In-hospital Mortality Among Adults with Sepsis and Hypotension. *JAMA* 2017;318(13):1233–40
50. de Oliveira FSV, Freitas FGR, Ferreira EM, *et al.* Positive fluid balance as a prognostic factor for mortality and acute kidney injury in severe sepsis and septic shock. *J Crit Care* 2015;30(1):97–101.
51. Acheampong A, Vincent J-L. A positive fluid balance is an independent prognostic factor in patients with sepsis.*Crit Care* 2015;19:251.
52. Liu VX, Fielding-Singh V, Greene JD, et al. The Timing of Early Antibiotics and Hospital Mortality in Sepsis.*Am J Respir Crit Care Med* 2017;196(7):856–63.
53. Rhodes A, Evans LE, Alhazzani W, et al. Surviving Sepsis Campaign: International Guidelines for Management of Sepsis and Septic Shock: 2016. *Int Care Med* 2017;43(3):304–77.
54. National Guideline Centre (UK). Sepsis: Recognition, Assessment and Early Management. National Institute for Health and Care Excellence (UK), London; 2016. PMID: 27441326.
55. Songer T. Before and after studies in injury research. Aufgerufen am. 2008; 13:2015.
56. Bhura M, Ariff S, Qazi SA, et al. Evaluating implementation of "management of Possible Serious Bacterial Infection (PSBI) when referral is not feasible" in primary health care facilities in Sindh province, Pakistan. *Plos one*. 2020 Oct 14;15(10):e0240688.

Figure Legends

Figure 1: Flow of participants through the study

Figure 2: Components of FAST-M Intervention

Figure 3: Completion of FAST-M bundle within 1 hour of recognition of maternal sepsis

Figure 4: Completion of FAST-M bundle within 1 hour of recognition of maternal sepsis according to time point

For peer review only

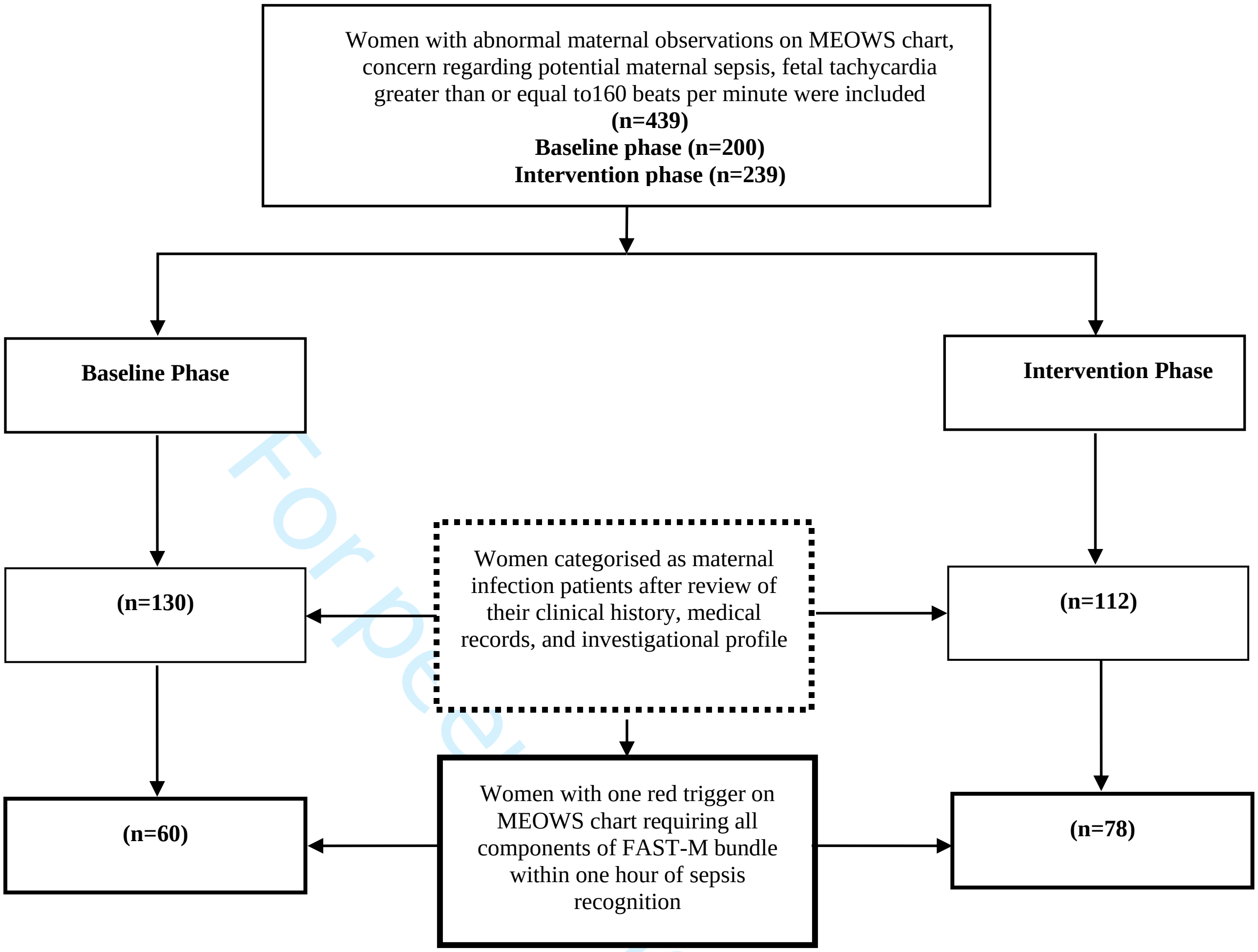

Figure 1: Flow of participants through the study

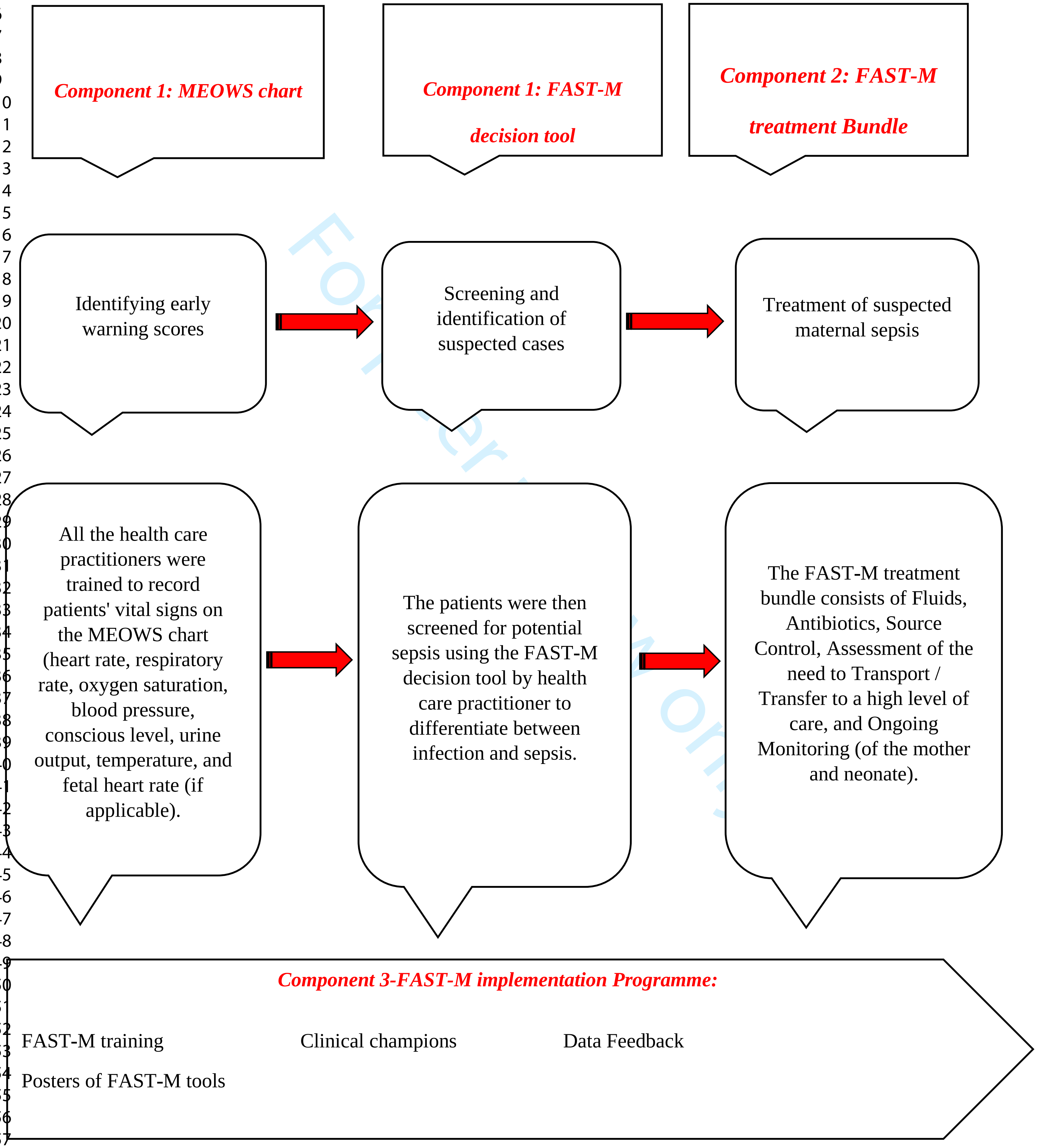

Figure 2: Components of FAST-M Intervention

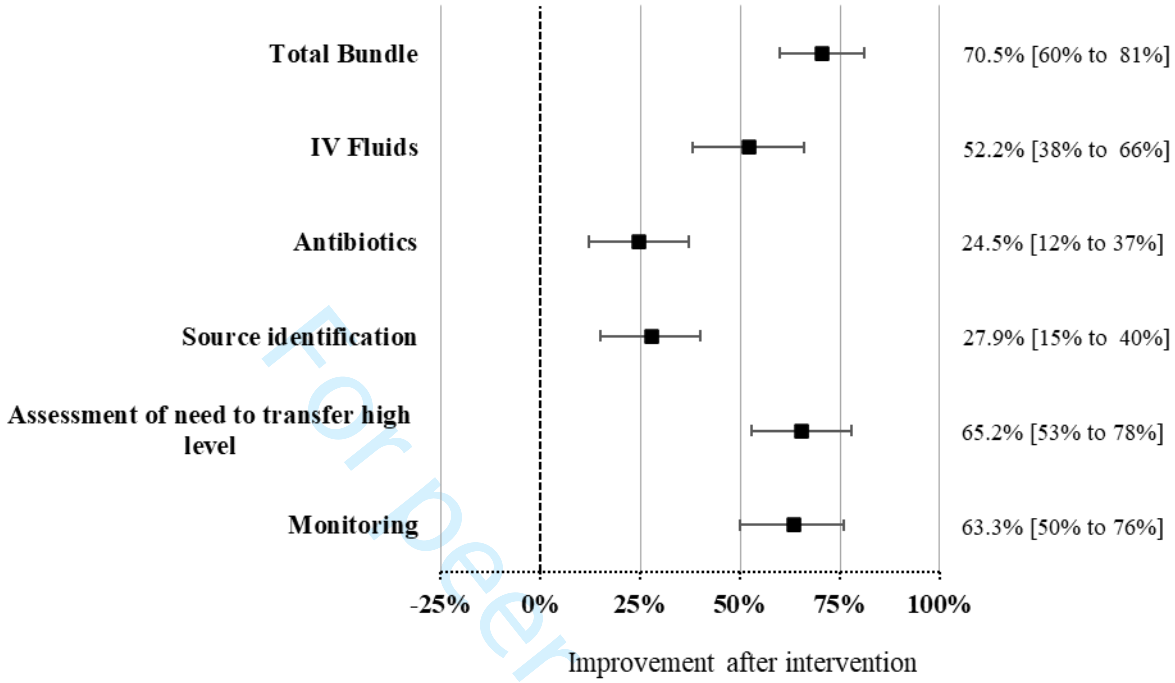

Figure 3: Completion of FAST-M bundle within 1 hour of recognition of maternal sepsis

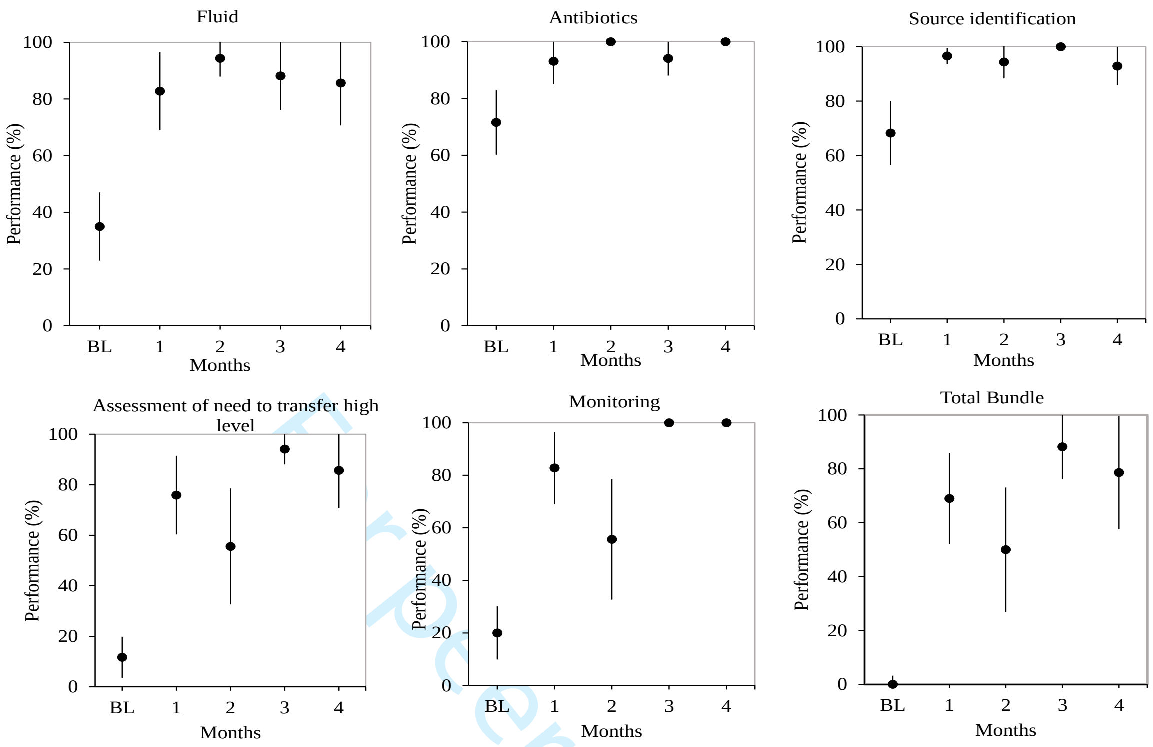

Figure 4: Completion of FAST-M bundle within 1 hour of recognition of maternal sepsis according to time point

MODIFIED EARLY OBSTETRIC WARNING CHART (MEOWS CHART)

FAST-M

Contact clinical decision maker if patient triggers ONE RED or TWO YELLOW flags at any one time.

Patient		Patient ID		DOB/Age	

WRITE VALUES IN BOXES PROVIDED

		Date															
		Time															
		Initials															
Respiratory rate (breaths per minute)	25 or more	RED															
	21 - 24	YELLOW															
	11 - 20	NORMAL															
	10 or less	RED															
Oxygen saturations (%)	95 or more	NORMAL															
	94 or less OR needing oxygen	RED															
Temperature (°C)	38 or more	YELLOW															
	36.0 to 37.9	NORMAL															
	35.9 or less	YELLOW															
Heart rate (beats per minute)	120 or more	RED															
	100 - 119	YELLOW															
	50 - 99	NORMAL															
	40 - 49	YELLOW															
	39 or less	RED															
Systolic blood pressure (mmHg)	160 or more	RED															
	140 -159	YELLOW															
	100 -139	NORMAL															
	90 - 99	YELLOW															
	89 or less	RED															
Diastolic blood pressure (mmHg)	110 or more	RED															
	90 -109	YELLOW															
	40 - 89	NORMAL															
	39 or less	RED															
Urine output Hours since patient passed urine (tick box)	12 hours or less	NORMAL															
	12 - 18 hours	YELLOW															
	18 hours or more OR less than 0.5 mL/kg/hour	RED															
Mental State (tick box)	Alert	NORMAL															
	Not Alert	RED															
Looks unwell (tick box)	No	NORMAL															
	Yes	YELLOW															
TOTAL YELLOW FLAGS																	
TOTAL RED FLAGS																	
ACTION TAKEN (IF REQUIRED) Yes (Y) / No (N)																	

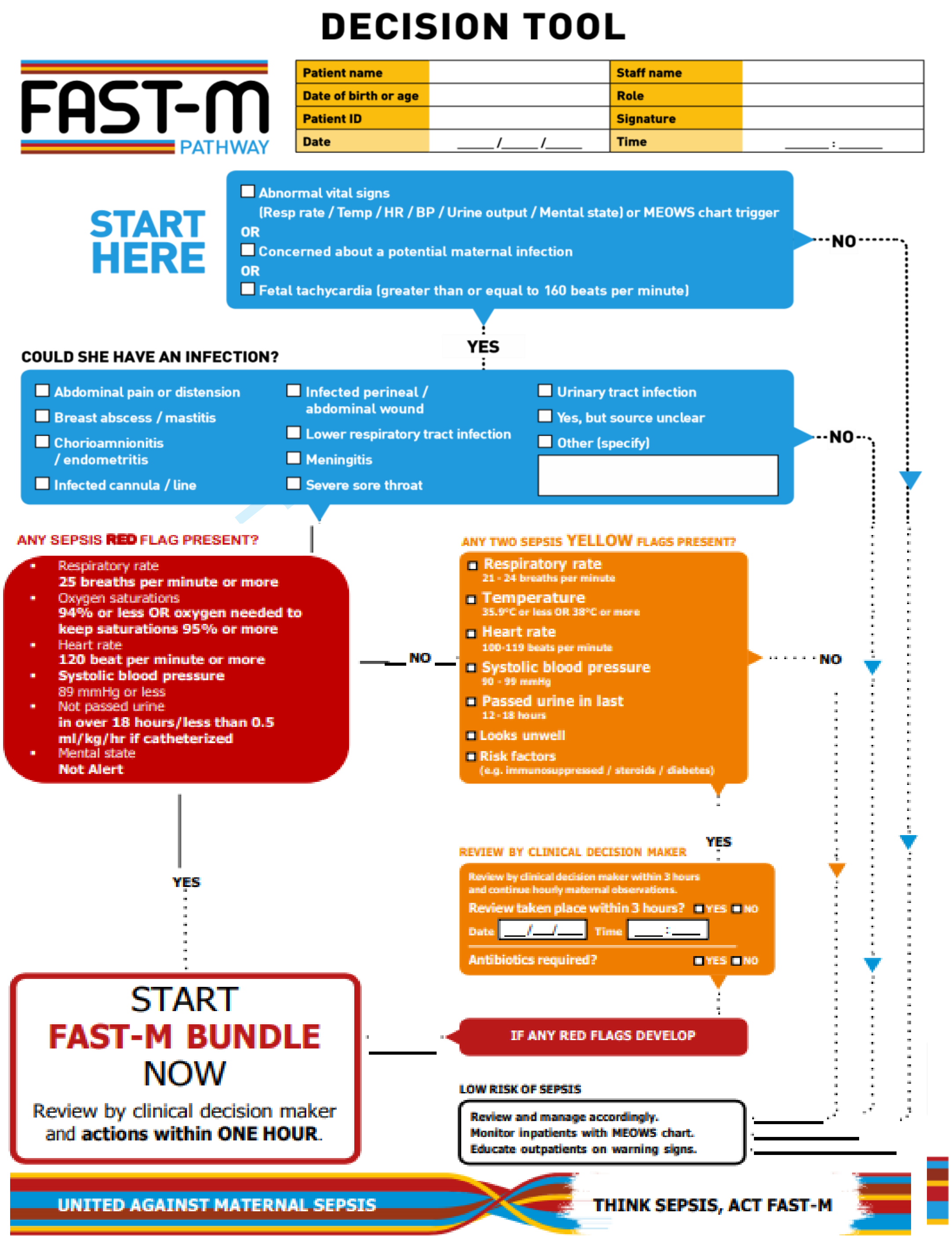
DECISION TOOL
FAST-M
PATHWAY
Patient name
Date of birth or age
Patient ID
Date ___/___/___
Staff name
Role
Signature
Time ___:___
START HERE
Abnormal vital signs
(Resp rate / Temp / HR / BP / Urine output / Mental state) or MEOWS chart trigger
OR
Concerned about a potential maternal infection
OR
Fetal tachycardia (greater than or equal to 160 beats per minute)
NO
YES
COULD SHE HAVE AN INFECTION?
Abdominal pain or distension
Breast abscess / mastitis
Chorioamnionitis / endometritis
Infected cannula / line
Infected perineal / abdominal wound
Lower respiratory tract infection
Meningitis
Severe sore throat
Urinary tract infection
Yes, but source unclear
Other (specify)
NO
ANY SEPSIS RED FLAG PRESENT?
Respiratory rate
25 breaths per minute or more
Oxygen saturations
94% or less OR oxygen needed to keep saturations 95% or more
Heart rate
120 beat per minute or more
Systolic blood pressure
89 mmHg or less
Not passed urine
in over 18 hours/less than 0.5 ml/kg/hr if catheterized
Mental state
Not Alert
NO
ANY TWO SEPSIS YELLOW FLAGS PRESENT?
Respiratory rate
21 - 24 breaths per minute
Temperature
35.9°C or less OR 38°C or more
Heart rate
100-119 beats per minute
Systolic blood pressure
90 - 99 mmHg
Passed urine in last
12 - 18 hours
Looks unwell
Risk factors
(e.g. immunosuppressed / steroids / diabetes)
NO
YES
REVIEW BY CLINICAL DECISION MAKER
Review by clinical decision maker within 3 hours and continue hourly maternal observations.
Review taken place within 3 hours? YES NO
Date ___/___/___ Time ___:___
Antibiotics required? YES NO
YES
START
FAST-M BUNDLE
NOW
Review by clinical decision maker and actions within ONE HOUR.
IF ANY RED FLAGS DEVELOP
LOW RISK OF SEPSIS
Review and manage accordingly.
Monitor inpatients with MEOWS chart.
Educate outpatients on warning signs.
UNITED AGAINST MATERNAL SEPSIS
THINK SEPSIS, ACT FAST-M

TREATMENT BUNDLE

FAST-M

Patient name		Staff name	
D.O.B or age		Role/Cadre	
Patient ID		Signature	

Date & time of red flag observation	__/__/__ __:__	Date & time bundle started	__/__/__ __:__	Date & time of review by clinical decision maker	__/__/__ __:__

REMEMBER TO COMPLETE THESE ACTIONS WITHIN ONE HOUR

1HR

F — FLUIDS (caution in pre-eclampsia, severe anaemia and pulmonary oedema)

Date	__/__/__	Time fluids initiated	__:__	Initials		Give 500 ml crystalloid immediately. Repeat 500 ml boluses to a maximum of 30 ml/kg if hypotension persists.
Details / reason not completed						

A — ANTIBIOTICS

Date	__/__/__	Time started	__:__	Initials		See antibiotic guidelines below
Details / reason not completed						

S — SOURCE – identify and treat the source of infection

Date	__/__/__	Time considered	__:__	Initials		See source identification and treatment boxes below
Details / reason not completed						

T — TRANSPORT (to higher level hospital or location within hospital, if required)

Date & time transport considered	__/__/__ __:__	Initials		Transport Required	☐ YES ☐ NO
Date & time transport requested	__/__/__ __:__	Initials		☐ N/A	
Date & time patient left facility	__/__/__ __:__	Initials			
Destination					
Reason for any delay					

M — MONITORING (start MEOWS chart if not already started. Repeat observations every 30 minutes until otherwise decided by clinical decision maker)

Date & time monitoring commenced	__/__/__ __:__	Details / reason not completed
Maternal / fetal monitoring should include	• Respiratory rate<br>• Oxygen Saturations<br>• Temperature<br>• Heart rate<br>• Blood pressure<br>• Urine output<br>• Mental state<br>• Fetal heart rate	
Neonatal monitoring and review commenced	☐ YES ☐ NO ☐ N/A	

ANTIBIOTIC GUIDELINES

Insert local guidance here

Immediate treatment for Maternal Sepsis:

- Ceftriaxone 2 g IV once daily (if no IV access this can be given as 2 IM injections of 1 g in different sites).
- If possible intra-abdominal source add Metronidazole 500 mg IV three times daily or 400 mg PO three times daily.

If above antibiotic regime is not available then give:

- Tazobactam 4.5 g IV daily two time a day
- Meropenem 1 g IV daily two times a day

IDENTIFY THE SOURCE

Consider

- Clinical history
- Clinical examination
- Blood tests (if available) (FBC, U&Es, LFTs, CRP, clotting)
- Blood cultures
- HIV and Malaria testing
- Urine sample
- Swabs (wound, vagina, throat)
- Sputum sample
- Imaging (abdominal, chest)
- Lumbar puncture
- Other ________________

REMOVE / TREAT THE SOURCE

Consider

- Malaria treatment
- Consider delivery of baby
- Removal of retained products of conception
- Debridement of wound / drainage of collection
- Removal of infected cannula / line
- Hysterectomy
- Targeted antibiotics once source known

UNITED AGAINST MATERNAL SEPSIS — THINK SEPSIS, ACT FAST-M

FORM 1: FACILITY AUDIT

FACILITY ID [|] – [| |]

Facility ID e.g. DH, KH *Visit ID e.g. 001, 002 etc.*

Today's Date: d d – m m – y y y y Time: h h : m m

Date previous form completed (or if first Visit, date study opened at this site): d d – m m – y y y y

Are you collecting the data during? **Baseline** ☐ **Intervention** ☐

3. How many of the following **MATERNAL OUTCOMES** have you had since the last visit or if first time since last 6 months?

Maternal Outcome	Number
Maternal Sepsis	
Maternal Deaths	
Post-Partum haemorrhage (>1L)	
Ante-Partum haemorrhage (>50ml)	
Severe pre-eclampsia/eclampsia (>160/110 and >2+protein in urine)	
Blood transfusions	
Uterine rupture	
Patient with reduced conscious level	
Injury (e.g. uterine, bladder, bowel)	
Anaphylaxis	
Pulmonary oedema	
Return to theatre	
Admission to ICU/HDU	
Broken down wounds (e.g. c/s, perineal)	

4. How many of the following **NEONATAL OUTCOMES** have you had since the last visit?

Neonatal Outcome	Number
Live Births	
Neonatal deaths before discharge from hospital	
Babies requiring antibiotics	
Sill births	
Admission to nursery care	
Apgar score of less than 7 at 5 mins after birth	

FORM 1: FACILITY AUDIT

FACILITY ID ☐☐ – ☐☐☐

Facility ID e.g. DH, KH *Visit ID e.g. 001, 002 etc.*

5. How many of the following **RESOURCES** are available today?

Resource	Amount			
	BAGS			
**Type of IV Fluids:**	0	1-5	6-10	>10
A) 0.9% Saline	☐	☐	☐	☐
B) Ringers Lactate	☐	☐	☐	☐
C) 5% dextrose	☐	☐	☐	☐
D) 50% dextrose	☐	☐	☐	☐

	**Type of oral antibiotics**			
**Type of oral antibiotics**	0	1-5	6-10	>10
A) Amoxicillin	☐	☐	☐	☐
B) Augmentin	☐	☐	☐	☐
C) Cephalosporin (e.g. Cefalexin)	☐	☐	☐	☐
D) Cefixime	☐	☐	☐	☐
E) Ciprofloxacin	☐	☐	☐	☐
F) Doxycycline	☐	☐	☐	☐
G) Clindamycin	☐	☐	☐	☐
H) Chloramphenical	☐	☐	☐	☐
I) Erythromycin	☐	☐	☐	☐
J) Metronidazole	☐	☐	☐	☐
K) Other (please state):	☐	☐	☐	☐

	**VIALS**			
**Type of IM / IV antibiotics**	0	1-5	6-10	>10
A) Amoxicillin	☐	☐	☐	☐
B) Ampicillin	☐	☐	☐	☐
C) Benzyl-penicillin	☐	☐	☐	☐
D) Penicillin G	☐	☐	☐	☐
E) Cefazolin	☐	☐	☐	☐
F) Cephalosporin (e.g. ceftriazone etc.)	☐	☐	☐	☐
G) Chloramphenical	☐	☐	☐	☐
H) Ciprofloxacin	☐	☐	☐	☐
I) Clindamycin	☐	☐	☐	☐
J) Erythromycin	☐	☐	☐	☐
K) Gentamycin	☐	☐	☐	☐
L) Metronidazole	☐	☐	☐	☐
M) Vancomycin	☐	☐	☐	☐
N) Other (please state)	☐	☐	☐	☐

	KITS			
**Manual vacuum aspiration (MVA) kits ready for use:**	0	1-5	6-10	>10
	☐	☐	☐	☐

* Good availability defined as a supply that is unlikely to run out before the next anticipated delivery

For peer review only

FORM 1: FACILITY AUDIT

FACILITY ID ☐☐ – ☐☐☐

Facility ID e.g. DH, KH *Visit ID e.g. 001, 002 etc.*

Resource	Availability		
	Good *	Limited	None
Equipment for IV line (cannula, dressings etc)	☐	☐	☐
Malaria tests	☐	☐	☐
Syphilis test	☐	☐	☐
HIV test	☐	☐	☐
Functioning theatre and staff able to remove source of infection	☐	☐	☐
Working thermometers	☐	☐	☐
Working BP machines	☐	☐	☐
Working O2 saturation machines(Pulse Oximeters)	☐	☐	☐
Fetoscopes / Pinnards/ fetal stethoscopes	☐	☐	☐
Clocks/watches	☐	☐	☐
Spare batteries	☐	☐	☐
Wound swabs	☐	☐	☐
Corona virus swabs	☐	☐	☐
Urine dips	☐	☐	☐
Sputum cultures	☐	☐	☐
Blood cultures	☐	☐	☐
Pregnancy tests	☐	☐	☐
Haematology blood tests	☐	☐	☐
Biochemistry blood tests	☐	☐	☐
Corona virus tests	☐	☐	☐
Blood taking equipment	☐	☐	☐
Functioning radiology department	☐	☐	☐
Functioning ultrasound machine and person to scan	☐	☐	☐
Functioning theatre and staff able to remove source of infection	☐	☐	☐
Weighing scales	☐	☐	☐
Urinary catheters	☐	☐	☐
Gloves	☐	☐	☐
Aprons	☐	☐	☐

For peer review only

6. How many of the following **STAFF** is available TODAY?

Resource	Number
Doctors	
Medical Officers	
Medical Students	
Registered Nurse	
Nursing students	
Hospital and Ward Attendants	

FORM 1: FACILITY AUDIT

FACILITY ID [ ][ ] – [ ][ ][ ]

Facility ID e.g. DH, KH *Visit ID e.g. 001, 002 etc.*

7. Are the following **FACILITIES** in working order TODAY?

Resource	Yes	No
Does the ward have electricity?		
Does the ward have running water?		
Does the ward have soap?		
Does the ward have alcohol gel?		
Are the autoclaves working?		
Are the washing facilities for staff available?		
Are the washing facilities for patients available?		
Are flushable toilet facilities for staff available?		
Are flushable toilet facilities for patients available?		

* Good availability defined as a supply that is unlikely to run out before the next anticipated delivery

Completed by: ______________________

Role: ______________________

Signature: ______________________

Date: *DD* / *MMM* / *YYYY*

You must have signed the Site Signature & Delegation Log

For peer review only

FORM 2: PATIENT CARE AUDIT FORM

Patient ID Number: ☐☐-☐☐☐

Facility ID e.g. A01, A02 etc *Unique ID e.g. 001, 002 etc*

Baseline ☐ **or** Intervention ☐

Date of audit: d d - m m - y y y y Time of audit: h h : m m

1. Clinical status at admission:

Antenatal ☐ Miscarriage ☐

Post-natal ☐ Termination of pregnancy ☐

2. Which of the following observations were recorded when the patient was first assessed?

	Yes	No	Not recorded
**Respiratory rate**			
**Saturations**			
**Temperature**			
**Heart rate**			
**Systolic BP**			

	Yes	No	Not recorded
**Diastolic BP**			
**Urine output**			
**Mental State**			
**Fetal heart rate**			

3. Were the observations recorded on a chart?

Yes ☐ No ☐ N/A ☐

→ If intervention phase, were they recorded on a MEOWS chart?
Yes ☐ No ☐

Page 1 of 2

For peer review only

FORM 2: PATIENT CARE AUDIT FORM

PATIENT ID NUMBER: ☐☐☐ - ☐☐☐☐

4. Has the patient been admitted for the last 24 hours

   Yes ☐ No ☐

   If yes, go to question 5, if No got to Question 6

5. During the last 24 hours, please state the number of times each observation has been taken and charted:

	Number of times
Respiratory rate	☐
Oxygen saturation	☐
Temperature	☐
Heart rate	☐
Systolic BP	☐

	Number of times
Diastolic BP	
Urine output	
Mental state	
Fetal heart rate	

For peer review only

REVIEW EVERY PATIENT'S NOTES WITH AN ABNORMAL OBSERVATION AND CONSIDER COMPLETING FORM 3 IF THERE IS CONCERN ABOUT MATERNAL INFECTION

Completed by: ______________________

Role: ______________________

Signature: ______________________ Date: DD / MMM / YYYY

You must have signed the Site Signature & Delegation Log

For peer review only - http://bmjopen.bmj.com/site/about/guidelines.xhtml

FORM 3: INDIVIDUAL PATIENT FORM

Screen the notes for every patient that had a suspected infection.
Only complete this form for patients that fulfil the criteria in q1-4 below.

Also complete this form for any patient you observe during the patient care audit (Form2) that fulfils the criteria below.

CONSIDER QUESTIONS 1-4 TO DETERMINE IF THIS FORM NEEDS TO BE COMPLETED

1. Was there a concern about a potential maternal infection?

Yes ☐ No ☐ → **DO NOT COMPLETE FORM**

Date and time suspected: d d - m m - y y y y h h : m m

2. Have observations taken?

Yes ☐ No ☐ → **COMPLETE FORM**

3. Was there a fetal tachycardia (heart rate) greater than or equal to 160 bpm?

Yes ☐ No ☐ Not Recorded ☐

4. Did the patient have abnormal vital signs or MEOWS chart trigger - 1 or more red flags or 2 or more yellow flags?

Yes ☐ No ☐

IF NO TO BOTH QUESTIONS, DO NOT COMPLETE FORM

For peer review only

FORM 3: INDIVIDUAL PATIENT FORM

Patient ID number: ☐☐☐☐ ☐☐☐☐

Date of assessment medical record: d D m m y y y y

Time of assessment medical record: ☐☐ ☐☐

5. **Study phase:**

Baseline ☐ Intervention ☐

6. At the time of assessment for maternal infection by the health care staff, were they an inpatient or outpatient?

Inpatient ☐ Outpatient ☐

→ Date of admission: d d - m m - y y y y

→ Whilst an inpatient, was a chart used for the patient? FAST-M MEOWS chart: ☐ Other chart ☐

If no, why not? ____________________

____

7. Were the patient referred from health facility

Yes ☐ No ☐

8. What treatment(s) did the patient receive before being referred? (Tick all the boxes that have been collected)

Fluids ☐ Antibiotics ☐ Source control ☐ Monitoring ☐ Not recorded ☐

9. What was the patient's pregnancy status when infection was first suspected/diagnosed?

<12 weeks ☐ 28+ weeks ☐ Miscarriage/ Abortion within the last 6 weeks ☐

12-28 ☐ Post-natal (up to 6 weeks) ☐

10. What type of infection was suspected? (Tick one or more options)

Offensive vaginal discharge ☐

Vaginal bleeding ☐

Delay in uterine involution ☐

Abnormal pain ☐

Urinary symptoms ☐

Vomiting/diarrhoea ☐

Breast swelling/pain

Cough/shortness of breath/sore throat ☐

Discharging wound/wound dehiscence ☐

Swollen /painful cannula site ☐

Fever /rigors/malaise ☐

Headache/Neck stiffness/rash ☐

Others, Please state ________________

For peer review only

FORM 3: INDIVIDUAL PATIENT FORM

PATIENT ID NUMBER ☐☐☐-☐☐☐

11. Did the patient have any of the following observations at time infection was first suspected/diagnosed (see q1)?

	Yellow trigger		Red trigger		No abnormal Observation	Not recorded
Respiratory rate	21-24		25 or more	10 or less	11 - 20	
	☐		☐	☐	☐	☐
Oxygen Saturations			94 or less Or needing oxygen		95 or more	
			☐		☐	☐
Temperature	38 or more	35.9 or less			36.0 to 37.9	
	☐	☐			☐	☐
Heart rate	100-119	40-49	120 or more	39 or less	50 – 99	
	☐	☐	☐	☐	☐	☐
Systolic Blood Pressure	140-159	90-99	160 or more	89 or less	100 -139	
	☐	☐	☐	☐	☐	☐
Diastolic Blood Pressure	90-109		110 or more	39 or less	40 – 89	
	☐		☐	☐	☐	☐
Not passed urine	In last 12-18 hours		In over 18 hours or less than 0.5ml/kg/hr.		12 hours or less	
	☐		☐		☐	☐
Appearance	Looks unwell				Look well	
	☐				☐	☐
Mental state			Not alert		Alert	
			☐		☐	☐

Date of observation d d - m m - y y y y

Time of observation h h m m

☐ or tick if not recorded

☐ or tick if not recorded

For peer review only

For peer review only - http://bmjopen.bmj.com/site/about/guidelines.xhtml

FORM 3: INDIVIDUAL PATIENT FORM **PATIENT ID NUMBER** ☐☐☐-☐☐☐

12. At the time the decision tool was initiated (if intervention phase) or if not used that the infection was most serious there were:

Yellow flags ___ (enter number)

Red flags ___ (enter number)

If 2 or more yellow flags, or 1 or more red flags was the FAST-M bundle initiated (if intervention phase)?

Yes ☐ No ☐

13. If one or more red flags present? Was the FAST-M bundle initiated (within 1 hour) (if intervention phase)?

Yes ☐ No ☐
Not recorded ☐ Go to question 12

14. If 2 or more yellow flags were present, did a review take place within 3 hours?

Yes ☐ No ☐ Not recorded ☐

15. Was the patient an inpatient for 24 hours or more prior to infection being suspected/ diagnosed?

Yes ☐ No ☐

16. If yes, During the last 24 hours or more, please state the number of times each observation has been recorded prior to infection being suspected / diagnosed:

	Number of times
Respiratory rate	
Temperature	
Heart rate	
Systolic BP	

	Number of times
Diastolic BP	
Urine output	
Mental state	
Fetal heart rate	

For peer review only

FORM 3: INDIVIDUAL PATIENT FORM **PATIENT ID NUMBER** ☐☐☐-☐☐☐

17. At the first time that a yellow or red trigger occurred, was action taken within 3 hours (if two or more yellow flags) or within 1 hour (if red)?

Yes ☐ No ☐ Not Recorded ☐

18. What action was taken (answer yes or no for each option)?

	Yes	No
Medical Officer informed	☐	☐
Nurse in charge informed	☐	☐
Resident Doctor informed	☐	☐
Treatment started	☐	☐
Consultant Doctor Informed	☐	☐
Nurse	☐	☐
Decision tool started, if intervention phase?	☐	☐

Others (please state) ________________

19. Who took action (answer yes or no for each option)?

	Yes	No
	☐	☐
Medical Officer	☐	☐
Resident Doctor	☐	☐
Consultant Doctor		
Medical assistant	☐	☐

	Yes	No
Nurse	☐	☐
Nurse In-charge	☐	☐

20. If the patient was in an outpatient setting was the patient subsequently admitted?

Yes ☐ No ☐ N/A ☐

21. Was the patient commenced on an observation chart?

Yes ☐ No ☐

For peer review only

FORM 3: INDIVIDUAL PATIENT FORM **PATIENT ID NUMBER** ☐☐☐-☐☐☐

22. In the 24 hours or more after suspecting maternal infection, did the patient have their observations repeated WHILST they were awaiting the clinical review?

Yes ☐ No ☐ No, because review happened at the same time ☐

Not Done ☐

	½ Hourly	Hourly	Less Frequent than hourly
Respiratory rate			
Temperature			
Heart rate			
Systolic BP			

	½ Hourly	Hourly	Less Frequent than hourly
Diastolic BP			
Urine output			
Mental state			
Fetal heart rate			

23. Did the patient receive a clinical review within 1 or 3 hours' review of recognition of maternal infection/sepsis?

Yes ☐ No ☐

Date and time of review d d - m m - y y y y h h : m m

For peer review only

FORM 3: INDIVIDUAL PATIENT FORM **PATIENT ID NUMBER** ☐☐☐ - ☐☐☐

a. Did the patient receive a clinical review within 3 hours? Yes ☐ No ☐
b. Did the patient receive a clinical review within 1 hour? Yes ☐ No ☐

24. Following the clinical review was the specified frequency of observations given?
Yes ☐ No ☐

25. Did the patient undergo observations at this specified frequency?
Yes ☐ No ☐

26. Was the patient treated for maternal infection/sepsis?
Yes ☐ No ☐

27. If intervention phase was the FAST-M decision tool was used for the patient?
Yes ☐ No ☐

MANAGEMENT

28. Were IV fluids given?

Yes ☐ No ☐ Why not? ______________________________

Date of started: d d - m m - y y y y Time of started h h m m

☐ or tick if not recorded ☐ or tick if not recorded

29. Were antibiotics administered within one hour?

Yes ☐ No ☐ Why not? ______________________________

Date of administered: d d - m m - y y y y Time of administered: h h m m

☐ or tick if not recorded ☐ or tick if not recorded

For peer review only

FORM 3: INDIVIDUAL PATIENT FORM

PATIENT ID NUMBER ☐☐☐-☐☐☐

SOURCE CONTROL/ IDENTIFICATION

30. Was an attempt made to identify the source of infection?

Yes ☐ No ☐ Why not? ______________________________

Date: d d - m m - y y y y Time: h h m m

☐ or tick if not recorded ☐ or tick if not recorded

What attempts were made to identify the source?

	Yes	No		Yes	No		Yes	No
Clinical history	☐	☐	HIV test	☐	☐	Imaging (abdominal, chest)	☐	☐
Clinical examination	☐	☐	Malaria test	☐	☐	Lumbar puncture	☐	☐
Blood test (if available)	☐	☐	Urine sample	☐	☐	Sputum sample	☐	☐
Blood cultures	☐	☐	Swabs (wound, vagina, throat)	☐	☐	Others (please state) _______________		

31. Was the source identified?

Yes ☐ No ☐

Date: d d - m m - y y y y Time: h h m m

☐ or tick if not recorded ☐ or tick if not recorded

For peer review only

FORM 3: INDIVIDUAL PATIENT FORM

PATIENT ID NUMBER ☐☐☐-☐☐☐

What was the source of infection?

	Yes	No
Breast	☐	☐
Chorioamnionitis	☐	☐
Endometritis	☐	☐
Infected Cannula/ line	☐	☐
Malaria	☐	☐
Meningitis/ cerebral infection	☐	☐
Other wound/ skin infection	☐	☐
Peritonitis	☐	☐
Respiratory infection	☐	☐
Urinary Tract Infection	☐	☐

Other (please state)________________________

32. Was the source treated?

Yes ☐ No ☐ Why not? ______________________________

Date: d d - m m - y y y y

☐ or tick if not recorded

Time: h h m m

☐ or tick if not recorded

How was it treated?

	Yes	No
Delivery of the baby	☐	☐
Removal of retained products of conception	☐	☐
Hysterectomy	☐	☐
Removal of infected cannula/line	☐	☐
Targeted antibiotics	☐	☐
Malaria treatment	☐	☐
Antivirals	☐	☐
Incision and drainage	☐	☐
Percutaneous drainage	☐	☐
Debridement of wound	☐	☐
Laparotomy and washout	☐	☐

Other (Please state) ____________________

For peer review only

FORM 3: INDIVIDUAL PATIENT FORM **PATIENT ID NUMBER** ☐☐☐-☐☐☐

TRANSPORT

33. Was there a need to transport the patient to a higher level of care considered?

Yes ☐ No ☐ Why not? ____________ Not recorded ☐

Date: dd-mm-yyyy ☐ or tick if not recorded

Time: hh mm ☐ or tick if not recorded

34. Was the patient transferred?

Yes ☐ No ☐ Why not? ____________ Not recorded ☐

Date requested: dd-mm-yyyy ☐ or tick if not recorded

Time requested: hh mm ☐ or tick if not recorded

Date patient left the facility: dd-mm-yyyy ☐ or tick if not recorded

Time: hh mm ☐ or tick if not recorded

35. Where was the patient transferred to? ____________

36. Was a transfer letter sent with the patient?

Yes ☐ No ☐ Why not? ____________

For peer review only

FORM 3: INDIVIDUAL PATIENT FORM PATIENT ID NUMBER ☐☐☐-☐☐☐

MONITORING

37. Did the patient have ongoing monitoring commenced after the recognition of maternal suspected/sepsis?

Yes ☐ No ☐ → N/A (24hrs hadn't elapsed) ☐

	1/2 hourly or Less	Hourly or Less	Daily or More	Less than Daily	not don e
Respiratory rate	☐	☐	☐	☐	☐
Temperatu re	☐	☐	☐	☐	☐
Heart rate	☐	☐	☐	☐	☐
Systolic BP	☐	☐	☐	☐	☐

	½ hourly or Less	Hourly or Less	Daily or More	Less than Daily	not done
Diastolic BP	☐	☐	☐	☐	☐
Urine output	☐	☐	☐	☐	☐
Neuro Response	☐	☐	☐	☐	☐
Fetal heartrate	☐	☐	☐	☐	☐

For peer review only

FORM 3: INDIVIDUAL PATIENT FORM

PATIENT ID NUMBER ☐☐☐-☐☐☐

PATIENT OUTCOMES

38. Did the patient have any of the following complications recorded during their treatment?

	Yes	No
Pulmonary oedema	☐	☐
Blood transfusion	☐	☐
Return to theatre	☐	☐
Allergic reaction	☐	☐

	Yes	No
Change in mental state	☐	☐
Reduced urine output (no output for >18 hours or <0.5ml/kg/hr)	☐	☐
Other	☐	☐
Please state	☐	☐

39. Was the patient admitted to high dependency/ intensive care during their treatment?

Yes ☐ No ☐

No. of days: ☐ Days

40. Did the patient have any of the following near miss events recorded during their stay?

Cardiovascular dysfunction		
**Event**	**Yes**	**No**
Shock	☐	☐
Cardiac arrest	☐	☐
Severe hypo perfusion (lactate >5mmol/L)	☐	☐
Severe acidosis (pH<7.1)	☐	☐
Use of continuous vasoactive drugs	☐	☐
Cardio-pulmonary resuscitation	☐	☐
**Respiratory dysfunction**		
Acute cyanosis	☐	☐
Gasping	☐	☐
Severe tachypnea (respiratory rate >40)	☐	☐
Severe bradypnea (respiratory rate <6)	☐	☐
Severe hypoxaemia (O2 sats <90% for >60 mins or PAO2/FiO2 <200)	☐	☐
Intubation and ventilation not related to anaesthesia	☐	☐

For peer review only

FORM 3: INDIVIDUAL PATIENT FORM **PATIENT ID NUMBER** ☐☐☐-☐☐☐

PATIENT OUTCOMES

**Renal dysfunction**		
Oliguria non-responsive to fluids or diuretics	☐	☐
Creatinine >300 µmol/ml or >3.5 mg/dL	☐	☐
Dialysis for acute renal failure	☐	☐
**Coagulation dysfunction**		
Failure to form clots	☐	☐
Severe acute thrombocytopenia (50,000 platelets/ml)	☐	☐
Massive transfusion of blood or red cells (≥5 units)	☐	☐
**Hepatic dysfunction**		
Jaundice in the presence of pre-eclampsia	☐	☐
Severe acute hyperbilirubinemia (bilirubin>100 µmol/L or >6.0 mg/dL)	☐	☐
**Neurological dysfunction**		
Prolonged unconsciousness or coma (lasting >12 hours)	☐	☐
Stroke	☐	☐
Uncontrollable fit / status epilepticus	☐	☐
Global paralysis	☐	☐
**Uterine dysfunction**		
Hysterectomy due to uterine infection or haemorrhage	☐	☐

For peer review only

FORM 3: INDIVIDUAL PATIENT FORM

PATIENT ID NUMBER ☐☐☐-☐☐☐

PATIENT OUTCOMES

41. Date of final discharge from hospital/health centre: d d - m m - y y y y

42. Total number of days spent in hospital: ☐ days

43. What was the outcome of the patient at discharge?

Alive and well, no follow-up required ☐

Alive and well, follow-up arranged ☐

Death ☐

Date of death: d d - m m - y y y y

Time of Death: h h : m m

Cause of death: ____________________

PREGNANCY OUTCOMES (if event occurred during same admission)

Tick if not applicable ☐

44. What was the pregnancy outcome?

Miscarriage ☐

Ectopic pregnancy ☐

Induced abortion ☐

Date occurred or diagnosed: d d - m m - y y y y

Live birth ☐

Still birth (> 28 weeks) ☐

Pregnancy terminated before admission ☐

For peer review only

FORM 3: INDIVIDUAL PATIENT FORM

PATIENT ID NUMBER ☐☐☐ - ☐☐☐

45. Was monitoring of the new-born commenced?

Yes ☐ No ☐ N/A ☐

Date: d d - m m - y y y y

☐ or tick if not recorded

Time: h h m m

☐ or tick if not recorded

46. Was fetal monitoring done if the patient was antenatal?

Yes ☐ No ☐ N/A ☐

Date: d d - m m - y y y y

☐ or tick if not recorded

Time: h h m m

☐ or tick if not recorded

For peer review only

FORM 3: INDIVIDUAL PATIENT FORM

PATIENT ID NUMBER ☐☐☐-☐☐☐

NEONATAL OUTCOMES AT BIRTH

Tick if not applicable ☐

47. Gestation: ☐ Months OR ☐ weeks ☐ days

48. Weight: ☐ g Not recorded ☐

49. Did the baby have any of the following complications?

	Yes	No	Not recorded
Resuscitation	☐	☐	☐
Agpars below 7 at 5 mins after birth	☐	☐	☐
Admitted to neonatal unit/special care	☐	☐	☐
Other (Please state)	☐	☐	________

50. Was the baby thought to have sepsis?

Yes ☐ No ☐

(If No →) Did the baby receive antibiotic prophylaxis anyway?

Yes ☐ No ☐

(If Yes →) Were they treated for sepsis?

Yes ☐ No ☐ Not recorded

51. What was the outcome of the baby at discharge?

Alive and well, no follow-up required ☐

Alive and well, follow-up required ☐

Death ☐

Date of Death d d - m m - y y y y

Time of Death: h h m m

Cause of Death ________

For peer review only

FORM 3: INDIVIDUAL PATIENT FORM

PATIENT ID NUMBER ☐☐☐-☐☐☐

Completed by: ______________________

Role: ______________________

Signature: ______________________ Date: DD / MM / YYYY

You must have signed the Site Signature & Delegation Log

For peer review only

Supplemental File 5

APPENDIX FIGURE 14 – **Participant Study ID Number: ___/_____– PATIENT CONSENT FORM (ENGLISH) for patients agreeing to access of their notes**

Title of study: Extension of the FAST- M maternal sepsis bundle in Pakistan, a feasibility study

What is the purpose of the overall study?

We are developing a way of improving the care of women with pregnancy or birth related infections around the world.

We are introducing three things: 1. a chart to help the nurses record your vital signs and detect infection before it becomes worse 2. A tool to help the medical staff treat infection 3. A training day to teach the medical staff about pregnancy or birth related infections

We hope that this will make caring for patients with pregnancy or birth related infections easier.

Why have I been invited to participate?

We are asking all patients pregnant or recently pregnant patients who are receiving treatment in hospital or who have an infection to participate. Being invited to take part does not necessarily mean you have an infection.

What will I have to do if I take part?

Nothing. If you agree to take part, you will receive the normal care. All we ask is that our research team from Aga Khan University Hospital can look at your medical notes to see what happened to you whilst you were in hospital. The information from your medical notes will help us decide if our study is working.

Do I have to take part?

No you do not have to take part it is your decision. If you don't want to take part that is fine, it will not affect how you are cared for when you are in hospital.

Will my taking part in the study be kept confidential?

Yes. All the information from your notes will be stored securely on a password protected computer system, which will be backed-up in a separate location to keep this information safe.

What are the possible benefits of taking part?

By giving us permission to look at your notes we hope that we will be able to find out if the study is working. If the study works, then this will hopefully help to improve the care of mothers with infections.

What will happen if I don't want to carry on with the study?

You may withdraw from the study without giving a reason for up to one month after. If you wish to withdraw we will delete all the information we had collected about you.

What if there is a problem?

For peer review only

If you have a concern about any aspect of this study, you can speak to the researchers, who will do their best to answer your questions. Their contact details are on the next page.

Who is organizing the research?

This study is being carried out by the Aga Khan University Hospital(AKUH), Pakistan in affiliation with University of Birmingham, UK and University of Liverpool, UK.

AKUH team will be facilitating the day to day running of the study, including looking at your medical notes and collecting the information.

Who has reviewed the study?

This study has been reviewed by the National Bioethics Committee Pakistan and AKUH Research Ethics Committee.

Contact details:

Lead Facilitator: Dr Sheikh Irfan Ahmed, Senior Instructor, AKUH National stadium road, Karachi

Email: sheikh.irfan@aku.edu   Telephone number: +92-021-34864650

Registered study ID number:

PLEASE INITIAL THE BOXES IF YOU AGREE WITH EACH SECTION:

1. I understand that my participation is voluntary and that I am free to withdraw up to one month after my participation without giving any reason. ☐

2. I agree for my notes to be accessed by the AKUH research team. I understand that all data collected about me will be anonymised and will not contain any identifiable information. ☐

3. I understand that the researchers might publish the results of this study. I give permission for anonymized information from my notes to be used for this purpose. ☐

4. I know how to contact the research team if I need to. ☐

5. I am happy for information about me might also be retained to include it anonymously in future studies

6. I agree to participate in this study. ☐

SIGNATURES:

_____________________________ ____________ ________________________ Patient name and surname

Date Signature / Thumbprint

For peer review only

____________________________ ____________ ________________________ Name and Surname of person
Date Signature taking consent

Participant Study ID Number: _____/_____

For peer review only

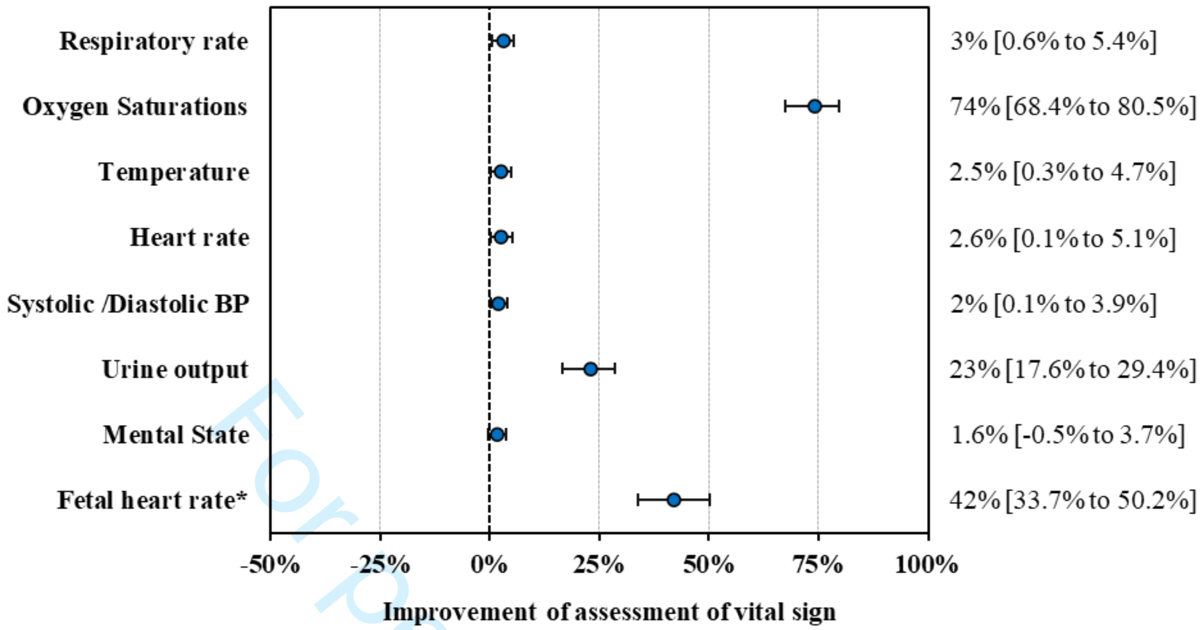

Supplement Figure 6: Patient's assessment of vital signs on admission (n=439)

For peer review only

Supplemental File 7

Table: Characteristics of suspected maternal sepsis patients (n=138)

Variables	Baseline	Intervention
	n=60	n=78
**Gestational at presentation**		
<12 weeks	2(3.3%)	0(0%)
12-28 weeks	2(3.3%)	1(1.3%)
>28 weeks	31(51.7%)	38(48.7%)
Post-natal (up to 6 weeks)	25(41.7%)	39(50%)
**Abnormal Trigger**		
Respiratory Rate (>25 or <10 per minute)	41(68.3%)	41(52.6%)
Temp (>38 or <35.9 °C)	25(41.7%)	35(44.9%)
Heart Rate (>120 or <39 beats per minute)	32(53.3%)	38(48.7%)
Systolic Blood Pressure (>160 or <89 mmhg)	43(71.7%)	43(55.1%)
Diastolic Blood Pressure (>110 or <39 mmhg)	35(58.3%)	38(48.7%)
Urine Output (In over 18 hours or less than 0.5ml/kg/hr)	4(6.7%)	4(5.1%)
Mental State (Not alert)	9(15%)	28(35.9%)
Appearance (Looks unwell)	7(11.7%)	49(62.8%)
**Source of maternal infection/sepsis***		
Breast	1(1.7%)	1(1.3%)
Chorioamnionitis	13(21.7%)	7(9%)
Endometritis	4(6.7%)	2(2.6%)
Infected Cannula/ line	1(1.7%)	0(0%)
Malaria	0(0%)	5(6.4%)
Meningitis/ cerebral infection	1(1.7%)	0(0%)
Other wound/ skin infection	3(5%)	5(6.4%)
Peritonitis	2(3.3%)	7(9%)
Respiratory infection	29(48.3%)	14(17.9%)
Urinary Tract Infection	9(15%)	6(7.7%)
Others	13(21.7%)	31(39.7%)
Unknown	7(11.7%)	14(17.9%)

*Thirty patients had multiple infection

For peer review only

Supplemental file 8

Table: Laboratory investigations of suspected maternal sepsis patients

Laboratory Investigations	Baseline phase (n=60)	Intervention phase (n=78)
Blood test (if available)	55 (92%)	78 (100%)
Blood cultures	27 (45%)	67 (86%)
HIV test	52 (87%)	72 (92%)
Malaria test	1 (2%)	21 (27%)
Urine sample	10 (17%)	63 (81%)
Swabs (wound, vagina, throat)	9 (15%)	2 (3%)
Imaging (abdominal, chest)	8 (13%)	38 (49%)
Lumbar puncture	0 (0%)	2 (3%)
Sputum sample	2 (3%)	2 (3%)

For peer review only

Supplemental File 9

Table: Assessment of Maternal statistics (from facility audit CRF1)

Total Admissions (N=24,000)	January to June 2021 (n=11205)	July to December 2021 (n=12795)	P-value
Maternal Sepsis	210 (1.8%)	160 (1.2%)	.0005
Maternal Mortality	159 (1.4%)	120 (0.9%)	.001
Post-Partum Hemorrhage	110 (0.9%)	175 (1.36%)	.006
Antepartum Hemorrhage	194 (1.73%)	110 (0.85%)	.0005
Severe Pre-Eclampsia	133 (1.18%)	88 (0.68%)	.0005
Uterine Rupture	36 (0.32%)	28 (0.21%)	.125

For peer review only

STROBE Statement—checklist of items that should be included in reports of observational studies

	Item No	Recommendation	Page No
**Title and abstract**	1	(*a*) Indicate the study's design with a commonly used term in the title or the abstract	1,3
		(*b*) Provide in the abstract an informative and balanced summary of what was done and what was found	3
**Introduction**			
Background/rationale	2	Explain the scientific background and rationale for the investigation being reported	5-7
Objectives	3	State specific objectives, including any prespecified hypotheses	7
**Methods**			
Study design	4	Present key elements of study design early in the paper	7
Setting	5	Describe the setting, locations, and relevant dates, including periods of recruitment, exposure, follow-up, and data collection	7-12
Participants	6	(*a*) *Cohort study*—Give the eligibility criteria, and the sources and methods of selection of participants. Describe methods of follow-up<br>*Case-control study*—Give the eligibility criteria, and the sources and methods of case ascertainment and control selection. Give the rationale for the choice of cases and controls<br>*Cross-sectional study*—Give the eligibility criteria, and the sources and methods of selection of participants	7
		(*b*) *Cohort study*—For matched studies, give matching criteria and number of exposed and unexposed<br>*Case-control study*—For matched studies, give matching criteria and the number of controls per case	
Variables	7	Clearly define all outcomes, exposures, predictors, potential confounders, and effect modifiers. Give diagnostic criteria, if applicable	11
Data sources/ measurement	8*	For each variable of interest, give sources of data and details of methods of assessment (measurement). Describe comparability of assessment methods if there is more than one group	12
Bias	9	Describe any efforts to address potential sources of bias	11-12
Study size	10	Explain how the study size was arrived at	12-13
Quantitative variables	11	Explain how quantitative variables were handled in the analyses. If applicable, describe which groupings were chosen and why	12
Statistical methods	12	(*a*) Describe all statistical methods, including those used to control for confounding	12
		(*b*) Describe any methods used to examine subgroups and interactions	
		(*c*) Explain how missing data were addressed	
		(*d*) *Cohort study*—If applicable, explain how loss to follow-up was addressed<br>*Case-control study*—If applicable, explain how matching of cases and controls was addressed<br>*Cross-sectional study*—If applicable, describe analytical methods taking account of sampling strategy	

For peer review only

(*e*) Describe any sensitivity analyses

Continued on next page

For peer review only

For peer review only - http://bmjopen.bmj.com/site/about/guidelines.xhtml

**Results**			
Participants	13*	(a) Report numbers of individuals at each stage of study—eg numbers potentially eligible, examined for eligibility, confirmed eligible, included in the study, completing follow-up, and analysed	8,12-13
		(b) Give reasons for non-participation at each stage	12
		(c) Consider use of a flow diagram	8
Descriptive data	14*	(a) Give characteristics of study participants (eg demographic, clinical, social) and information on exposures and potential confounders	13
		(b) Indicate number of participants with missing data for each variable of interest	12-13
		(c) *Cohort study*—Summarise follow-up time (eg, average and total amount)	NA
Outcome data	15*	*Cohort study*—Report numbers of outcome events or summary measures over time	*NA*
		Case-control study—Report numbers in each exposure category, or summary measures of exposure	*NA*
		Cross-sectional study—Report numbers of outcome events or summary measures	12-15
Main results	16	(*a*) Give unadjusted estimates and, if applicable, confounder-adjusted estimates and their precision (eg, 95% confidence interval). Make clear which confounders were adjusted for and why they were included	NA
		(*b*) Report category boundaries when continuous variables were categorized	NA
		(*c*) If relevant, consider translating estimates of relative risk into absolute risk for a meaningful time period	NA
Other analyses	17	Report other analyses done—eg analyses of subgroups and interactions, and sensitivity analyses	12-15
**Discussion**			
Key results	18	Summarise key results with reference to study objectives	15
Limitations	19	Discuss limitations of the study, taking into account sources of potential bias or imprecision. Discuss both direction and magnitude of any potential bias	20-21
Interpretation	20	Give a cautious overall interpretation of results considering objectives, limitations, multiplicity of analyses, results from similar studies, and other relevant evidence	15-21
Generalisability	21	Discuss the generalisability (external validity) of the study results	21
**Other information**			
Funding	22	Give the source of funding and the role of the funders for the present study and, if applicable, for the original study on which the present article is based	22

*Give information separately for cases and controls in case-control studies and, if applicable, for exposed and unexposed groups in cohort and cross-sectional studies.

Note: An Explanation and Elaboration article discusses each checklist item and gives methodological background and published examples of transparent reporting. The STROBE checklist is best used in conjunction with this article (freely available on the Web sites of PLoS Medicine at http://www.plosmedicine.org/, Annals of Internal Medicine at http://www.annals.org/, and Epidemiology at http://www.epidem.com/). Information on the STROBE Initiative is available at www.strobe-statement.org.

For peer review only - http://bmjopen.bmj.com/site/about/guidelines.xhtml

BMJ Open

Early recognition and management of maternal sepsis in Pakistan: A feasibility study of the implementation of FAST-M Intervention

Journal:	*BMJ Open*
Manuscript ID	bmjopen-2022-069135.R1
Article Type:	Original research
Date Submitted by the Author:	08-Feb-2023

Complete List of Authors:	Ahmed, Sheikh; The Aga Khan University, Obs and Gynae<br>Rind , Ghulam Kubra; The Aga Khan University Hospital<br>Sikandar , Raheel ; Liaquat University of Medical and Health Sciences, OBGYN<br>Raza , Amir ; The Aga Khan University Hospital Main Campus Karachi<br>Khowaja, Bakhtawar; The Aga Khan University Hospital Main Campus Karachi<br>Parveen, Fahmida; Liaquat University of Medical and Health Sciences<br>Khan , Sehrish ; Liaquat University of Medical and Health Sciences<br>Memon, Nazia; Liaquat University of Medical and Health Sciences<br>Jahangir, Arshia ; The Aga Khan University<br>Mirza, Daayl ; Ziauddin University<br>Cheshire , James ; University of Birmingham<br>Dunlop, Catherine; University of Birmingham<br>Shakoor, Sadia; The Aga Khan University Hospital Main Campus Karachi<br>Barolia, Rubina; The Aga Khan University Hospital Main Campus Karachi<br>Sheikh, Lumaan; The Aga Khan University Hospital Main Campus Karachi<br>Saleem, Sarah; The Aga Khan University<br>Coomarasamy, Arri; University of Birmingham<br>Lissauer, David ; Malawi-Liverpool-Wellcome Trust Clinical Research Programme, ; University of Liverpool,

**Primary Subject Heading**:	Obstetrics and gynaecology
Secondary Subject Heading:	Infectious diseases, Obstetrics and gynaecology, Evidence based practice
Keywords:	OBSTETRICS, INFECTIOUS DISEASES, Maternal medicine < OBSTETRICS

SCHOLARONE™ Manuscripts

I, the Submitting Author has the right to grant and does grant on behalf of all authors of the Work (as defined in the below author licence), an exclusive licence and/or a non-exclusive licence for contributions from authors who are: i) UK Crown employees; ii) where BMJ has agreed a CC-BY licence shall apply, and/or iii) in accordance with the terms applicable for US Federal Government officers or employees acting as part of their official duties; on a worldwide, perpetual, irrevocable, royalty-free basis to BMJ Publishing Group Ltd ("BMJ") its licensees and where the relevant Journal is co-owned by BMJ to the co-owners of the Journal, to publish the Work in this journal and any other BMJ products and to exploit all rights, as set out in our licence.

The Submitting Author accepts and understands that any supply made under these terms is made by BMJ to the Submitting Author unless you are acting as an employee on behalf of your employer or a postgraduate student of an affiliated institution which is paying any applicable article publishing charge ("APC") for Open Access articles. Where the Submitting Author wishes to make the Work available on an Open Access basis (and intends to pay the relevant APC), the terms of reuse of such Open Access shall be governed by a Creative Commons licence – details of these licences and which Creative Commons licence will apply to this Work are set out in our licence referred to above.

Other than as permitted in any relevant BMJ Author's Self Archiving Policies, I confirm this Work has not been accepted for publication elsewhere, is not being considered for publication elsewhere and does not duplicate material already published. I confirm all authors consent to publication of this Work and authorise the granting of this licence.

For peer review only

Paper Title:

Early recognition and management of maternal sepsis in Pakistan: A feasibility study of the implementation of FAST-M Intervention

Corresponding Author*

Sheikh Irfan Ahmed

Aga Khan University Hospital, National Stadium Road, Karachi city, Pakistan. P.O. Box 3500 Postal code: 74800

Email: sheikh.irfan@aku.edu

Author Names:

Sheikh Irfan Ahmed[1]

Ghulam Kubra Rind[1]

Raheel Sikandar[2]

Amir Raza[1]

Bakhtawar M.Hanif Khowaja[1]

Fahmida Parveen [2]

Sehrish Khan[2]

Nazia Memon [2]

Arshia Jahangir[3]

Daayl Naim Mirza[4]

James Cheshire[5]

Catherine Dunlop [5]

Sadia Shakoor[1]

Rubina Barolia[1]

Lumaan Sheikh[1]

Sarah Saleem[1]

Arri Coomarasamy[5]

David Lissauer[6, 7]

Affiliations & full institutional mailing addresses of all authors

[1] Aga Khan University Hospital, National Stadium Road, Karachi city, Pakistan. P.O. Box 3500 Postal code: 74800

[2] LUMHS Hospital Liaquat University of Health and Medical Sciences, Hyderabad city, Pakistan. Postal Code: 76090

[3] Aga Khan University, National Stadium Road, Karachi city, Pakistan. P.O. Box 3500 Postal code: 74800

[4] Ziauddin University4/B Shahrah-e-Ghalib Rd, Block 6 Clifton, Karachi, Karachi City, Sindh 75000

For peer review only

[5] Institute of Metabolism and Systems Research, University of Birmingham, Edgbaston, Birmingham, UK, B15 2TT.

[6] Institute of Life Course and Medical Sciences, University of Liverpool, Liverpool, William Henry Duncan Building, Liverpool, UK, L7 8TX.

[7] Malawi-Liverpool-Wellcome Clinical Research Programme, Chichiri, Blantyre 3, Malawi

Email addresses

sheikh.irfan@aku.edu (SI)
ghulam.kubra@aku.edu (GK)
raheel.sikandar@lumhs.edu.pk (RS)
amir.raza@aku.edu (AR)
bakhtawar.hanif@aku.edu (BK)
dr_memon16@yahoo.com (FP)
drkhanhyd@gmail.com (SK)
nazia.memon23@yahoo.com (NM)
arshia.jahangir@scholar.aku.edu (AJ)
daayl.13094@zu.edu.pk (DM)
sadia.shakoor@aku.edu (SSH)
james.cheshire@nhs.net (JC)
catherinedunlop@nhs.net (CD)
rubina.barolia@aku.edu (RB)
lumaan.sheikh@aku.edu (LS)
sarah.saleem@aku.edu (SS)
A.Coomarasamy@bham.ac.uk (AC)
David.Lissauer@liverpool.ac.uk (DL)

Word Count: 4680

For peer review only

ABSTRACT

Objective: Maternal sepsis is the 3rd leading cause of maternal mortality globally. World Health Organization and collaborators developed a care bundle called FAST-M (**F**luids, **A**ntibiotics, **S**ource identification and treatment, **T**ransfer, and **M**onitoring) for early identification and management of maternal sepsis in low-resource settings. This study aimed to determine feasibility of FAST-M intervention in a low-resource setting in Pakistan. The FAST-M intervention consists of maternal sepsis screening tools, treatment bundle and implementation program.

Design and setting: A feasibility study with before and after design was conducted in women with suspected maternal sepsis admitted at the LUMHS hospital Hyderabad. The study outcomes were compared between baseline and intervention phase. In the baseline phase (2 months), the existing sepsis care practices were recorded, followed by a training program for healthcare providers on the application of FAST-M tools. These tools were implemented in the intervention phase (4 months) to assess any change in clinical practices compared to the baseline phase.

Results: During the FAST-M implementation, 439 women were included in the study. 242/439 were suspected maternal infection cases, and 138/242 were women with suspected maternal sepsis. The FAST-M bundle was implemented in women with suspected maternal sepsis. Following the FAST-M intervention, significant changes were observed. Improvements were seen in the monitoring of oxygen saturation measurements [25.5% vs 100%; difference: 74%; 95% CI:68.4%-80.5% p<0.01], fetal heart rate assessment [58% vs100%; difference:42.0%; 95%CI: 33.7% to 50.3%; p=<0.01], and measurement of urine output [76.5% vs 100%; difference 23.5%; 95%CI: 17.6% to 29.4%; p<0.01]). Women with suspected maternal sepsis received all components of the treatment bundle within one hour of sepsis recognition [0% vs70.5%; difference 70.5%; 95%CI: 60.4% to 80.6%; p<0.01].

Conclusion: Implementation of the FAST-M intervention was feasible and significantly improved the quality of maternal sepsis care, including enhanced early identification and management of maternal sepsis.

Trial registration number: ISRCTN17105658

For peer review only

Strengths and limitations:

- The study design provided the possibility to analyse operational, and practical factors for introducing the FAST-M intervention in the local setting.
- The tools used in the study were adapted in the context of Pakistan before its implementation.
- The study design does not account for the temporal effects, hence is exposed to possible selection and reporting bias.
- This study was conducted at only one tertiary level hospital.
- The intervention period was of only four months.

For peer review only

INTRODCUTION

Maternal sepsis is ‘a life-threatening condition defined as organ dysfunction resulting from infection during pregnancy, childbirth, post-abortion, or the post-partum period’.[1] Worldwide, it’s the 3rd most common cause of maternal mortality and represents around 11% of maternal deaths.[2] Pregnant patients and those that have recently delivered are more susceptible to maternal infections that can rapidly progress towards sepsis. [3,4]

Modelling studies, based on currently available data, observed that globally around 17000 maternal deaths per year may be attributed to maternal sepsis and other maternal infections (MSMI).[5] Approximately 5·7 million women suffered complications of maternal sepsis globally in 2017.[6] Annually, 99% of the 302,000 maternal deaths occurred in LMICs,[7] with the highest rates reported in Africa and Asia.[7,8] The overall maternal mortality ratio (MMR) in Pakistan is 186 per 100,000 live births in 2020,[9] and sepsis ranked as the 3rd leading cause of maternal deaths (16.3%)[10,11] following haemorrhage and eclampsia.[2]

In 2015, the Global maternal and neonatal sepsis initiative was introduced by the WHO and other partners to design strategies that could help in improving the recognition and management of maternal and neonatal sepsis in low-resource settings.[12] This initiative aimed to develop and test the effective strategies to prevent, detect, and manage the maternal and neonatal deaths caused by sepsis.[12] This included the key recommendation to develop a sepsis care bundle specific to the maternal population.

The use of care bundles in the management of sepsis has been found to reduce mortality and improve patient outcomes in high-income countries.[13] Most notably, the surviving sepsis campaign (SSC) bundles,[14,15] as well as the UK Sepsis Trusts' Sepsis 6 bundle[16] have been widely used.[15,16] Despite this, there was no sepsis care bundle in particular for the maternal population which can be implemented efficiently in low-resource settings.[17-19]

For peer review only - http://bmjopen.bmj.com/site/about/guidelines.xhtml

To address this, a care bundle for treatment of maternal sepsis in low resource settings was developed after reaching the consensus utilising a modified Delphi approach by an international health expert panel.[20] The selected components were Fluids, Antibiotics, Source identification and control, Transfer to an appropriate level of care, and ongoing Monitoring of mother and neonate. The bundle was named "FAST-M" as a memorable acronym for supporting implementation and communication.[20] The FAST-M bundle was first implemented in 15 government healthcare facilities in Malawi, and it was not only found to be feasible to implement but also resulted in improved clinical care.[21]

Despite existence of national sepsis guidelines for Pakistan (SGP), their uptake remains low especially for obstetric patients.[22] Additionally, there is uncertainty that the existing evidence-based approach to prevent, recognise, and treat maternal sepsis is effective and implementable in a low-resource setting. It was, therefore, planned to determine the feasibility of the FAST-M intervention in the local setting before recommending its implementation at scale. The study protocol and procedures have been described in detail and published elsewhere.[23]

To optimize the use of the FAST-M intervention in Pakistan, a qualitative study was conducted to understand the existing sepsis management practices/behaviours, and to identify potential gaps and resource availability in the local setting.[24] Based on the qualitative findings described in a separate paper and availability of resources in the facility, the FAST-M bundle care tools which were used in Malawi[21] were modified to plan a practical approach for the implementation of the intervention in our local context. [24] This study aimed to determine whether the FAST-M intervention is feasible to introduce for the early detection and management of maternal sepsis in a low-resource setting in Pakistan. We hypothesise that the FAST-M intervention in this adapted form could be successfully implemented and would then improve maternal sepsis care, including its early recognition and management.

For peer review only

METHODS

A prospective feasibility study with a before and after design was conducted at the Liaquat University of Medical and Health Sciences, (LUMHS) Hyderabad, Pakistan from the period of June 2021 to December 2021. Women admitted to obstetrics and gynecology units of the facility, who were pregnant, or within 6 weeks of miscarriage, ectopic pregnancy, termination of pregnancy, or delivery were considered eligible for this study. These eligible patients were monitored on MEOWS chart during their inpatient admission. Those who had abnormal maternal observations, or healthcare practitioner's concern regarding potential maternal infection, or pregnant women with a fetal tachycardia greater than or equal to 160 beats per minute were included in the study. There were no exclusion criteria for the study.

In the baseline phase, data were collected from facility record using CRF1 (facility audit form) to assess available resources for sepsis management and overall maternal outcomes in last six months. Data of patients who were meeting eligibility criteria (Fig.1.) were recorded on CRF 2 and CRF 3 to assess existing practices for management of maternal sepsis. Following this, the intervention phase was carried out. At first, HCPs were trained on the use of FAST-M bundle care tools (MEOWS chart, decision tool and treatment bundle) to early identify and manage sepsis patients. The HCPs training was an ongoing process throughout the implementation of intervention. The FAST-M bundle care tools were then implemented in intervention phase in the facility and data of patients meeting the eligibility criteria were recorded using CRF 2 and CRF 3. The flow of study participants has been demonstrated in **(Figure 1).**

Comparisons were made between baseline and intervention phase to assess any change in time and frequency of vital signs monitoring (on MEOWS chart) of patients, and application of all components of FAST-M bundle care tool (supplemental file 1) within an hour of sepsis recognition. Maternal outcomes (sepsis rate and related mortality) were also compared between baseline and intervention phase.

For peer review only

Intervention:

The planned intervention consisted of 3 components:

1. Maternal sepsis screening tools
2. FAST-M treatment bundle
3. FAST-M implementation Programme

Component 1- Maternal sepsis screening tools

Maternal sepsis screening tools included the Modified Early Obstetric Warning Scores chart (MEOWS) and the FAST-M decision tool **(Supplemental File 1**). MEOWS is the tool adapted by Malawi study based on an early warning score approach and simplified from the approach adopted in UK hospitals.[25] The tool assisted in charting an individual's physiological parameters over time with guiding thresholds that signal clinical action if they become abnormal.[26]

The MEOWS chart used in FAST-M implementation in the districts of Malawi [21] was adapted in the context of Pakistan for this study. During the adaptation phase, the health care practitioners recommended the inclusion of oxygen saturation into the MEOWs chart to determine patient's clinical condition considering the outbreak of COVID-19 infection and the availability of pulse oximeters at the study site. Therefore, the oxygen saturation component was incorporated in the modified MEOWS chart based on the scope of existing practices and the significance of this indicator in the identification of patient's clinical condition.

In the intervention period, eligible patients who had abnormal observations (indicated by a single red or two yellow flags) on MEOWS chart (Supplemental file 1) screened for potential sepsis using the FAST-M decision tool. Those who developed a red trigger on the MEOWS chart received an immediate clinical review. FAST-M treatment bundle was initiated within an hour on clinician's judgment on suspicion of sepsis and continued until advised to end the treatment.

For peer review only - http://bmjopen.bmj.com/site/about/guidelines.xhtml

Those patients who triggered two yellow flags on the MEOWS chart and were thought to have possible infection were reviewed within three hours. All suspected cases (those without red or two yellow triggers, were still considered for having a risk of sepsis and remained under observation for possible development of red or yellow triggers), these patients were monitored on the MEOWS chart hourly throughout the day and were managed as per local guidelines.

Component 2-FAST-M Treatment Bundle

The FAST-M treatment bundle consists of Fluids, Antibiotics, Source identification and Control, Assessment of the need to Transport / Transfer to a high level of care, and Ongoing Monitoring (of the mother and neonate). The first and second-line antibiotic therapy guidelines were modified as per the local context during the adaptation phase. [24] **(Supplemental File 1)**. Patients with suspected sepsis were commenced immediately on the FAST-M treatment bundle. Each patient's treatment was recorded on the treatment bundle, along with any actions taken and any reasons for not completing certain elements.

Component 3-FAST-M implementation Programme

The implementation program **(Figure 2)** consisted of the FAST-M training, selection of clinical champions, and feedback achieved from data collected during the baseline phase, and through the facility audit form.

The two-day training sessions were delivered by health care experts after the completion of the baseline phase, and before the implementation of the FAST-M bundle intervention. The training session started with classroom-based instruction on the FAST-M intervention followed by interactive sessions used as a strategy for hands-on training of healthcare providers on the FAST-M tools. Follow-up training sessions were also conducted every 2 weeks by the local team leads during the implementation of the FAST-M bundle intervention.

Health care practitioners including doctors and nurses (n= 40) working in obstetrics and gynecology units, internal medicine, operating rooms, emergency, and intensive care units of LUMHS hospital were trained to record patients' vital signs on the MEOWS chart (heart rate, respiratory rate, oxygen saturation, blood

For peer review only - http://bmjopen.bmj.com/site/about/guidelines.xhtml

pressure, conscious level, urine output, temperature, and fetal heart rate (if applicable). These observations were then charted on a MEOWS chart in the inpatient setting and then inserted into the patient's files.

The local clinical champions and team leaders were identified during training programs and trained to take the lead in implementation of the FAST-M intervention at the study site and remained engaged throughout the implementation process. These champions were selected from different hospital departments primarily involved in the management of maternal sepsis patient including obstetrics and gynecology units, emergency department, internal medicine, and intensive care unit. The selected champions included doctors and nurses.

The overarching goal of each champion was to encourage engagement and compliance with the FAST-M bundle. To achieve this goal, champions at each unit were engaged in several key activities that included disseminating knowledge, advocating, navigating boundaries, facilitating consensus, arranging meetings with stakeholders, tracking quality indicators, and developing organizational communication strategies and relationships. The patient records collected were also counter-checked by the clinical champions of the assigned units. These champions also helped the study team in the identification of patients with suspected sepsis and their enrolment in the study.

In addition, training certificates were distributed among the participants. Moreover, FAST-M tools (MEOWS chart, decision, and treatment tool) were displayed in the form of posters throughout all the units involved in the management of maternal sepsis and implementation of FAST-M intervention.

Study Period:

The study comprised a baseline period of two months in which usual practices to detect and treat maternal sepsis existing locally were assessed. The FAST-M training was conducted on the completion of the baseline period. Following training, an intervention period of four months was commenced to assess any change in practice.

For peer review only - http://bmjopen.bmj.com/site/about/guidelines.xhtml

Outcomes:

Primary process outcomes included

1) The proportion of patients admitted with indications of sepsis, who received appropriate monitoring (full set of vital sign measurements on admission recorded on MEOWS chart).

2) The proportion of women with suspected maternal sepsis receiving the FAST-M treatment bundle (including each bundle component) within 1 hour of identification of sepsis.

Secondary outcomes included

1) Rate of vital signs with complete recording of MEOWS chart components [heart rate, respiratory rate, oxygen saturation, blood pressure, mental state, urine output, temperature, and fetal heart rate (in pregnant women)].

2) Rate of application of FAST-M Bundle components [Fluids, Antibiotics, source identification (identified clinically) and control, assessment of the need to transport/ transfer to a high level of care, and ongoing monitoring (of the mother and neonate)].

3) Rate of maternal morbidity and mortality after FAST-M intervention.

Data Collection:

Quantitative data for this feasibility study was collected by a senior research assistant (SRA) on the facility record CRF 1 **(Supplement File 2)**, and patient notes including the MEOWS charts, FAST-M decision, and treatment tools onto the relevant paper-based CRFs 2 & 3 **(Supplemental File 3-4)** following patient consent **(Supplemental File 5)** Data were composed of various outcomes; structural, clinical, organization, and any adverse events. The data were maintained in an investigator file to be secured in a locked cabinet. Anonymised and deidentified patients' information recorded on the data collection sheet were recorded in a database located on a secure server.

During the study, biweekly monitoring of the study site was undertaken by the research coordinator, to observe practice, collect feedback and provide updates on the study site's performance, and evaluate any additional training requirements.

For peer review only

Statistical Analysis:

Statistical Package for Social Sciences (SPSS) version 19.0 (IBM Corporation, Armonk, NY, USA) and STATA 14 computer software (Stata Corporation, Texas USA) was used for data processing and statistical analysis. Data were reported in frequency and percentages for categorical observations. A Chi-square test for independence was used to compare components of FAST-M bundle between phases (baseline and intervention). The Chi-square test for trend (P trend) was used to observe the performance during the intervention phase. Statistical significance was determined at $P \leq 0.05$.

Patient and public involvement:

Patients were not involved in the design of this study. The FAST-M intervention was implemented on the enrolled patients however, they were not invited to comment on the study design, interpret the results or contribute to writing or editing of this document for accuracy of findings. Hospital staff participated in the implementation but were not involved in the analysis of findings.

RESULTS:

Vital signs monitoring assessment: 439 women were included in the study (200 in baseline and 239 in the intervention phase), with vital signs recorded on admission ***(Table 1)***. 242/439 were cases of suspected maternal infection, and 138/242 were women with suspected maternal sepsis. Figure 1 provides the summary of the screening, and enrolment of the study participants. Following the FAST-M implementation, significant improvements were seen in the assessment of oxygen saturations in the intervention phase compared to the baseline phase [Difference: 74%; 95%CI:68.4%-80.5% p<0.01], fetal heart rate assessment [Difference:42.02%; 95%CI: 33.7% to 50.28%; p=<0.01], measurement of urine output [Difference 23.5%; 95%CI: 17.6% to 29.4%; p<0.01]. Temperature, respiratory rate, heart rate, and blood pressure were measured in 97% of the cases in the baseline phase but showed an increase of almost 3% in the intervention phase [Difference 2.5% 95%CI: 0.34% to 4.66%; p≤0.05]. No significant difference was found in the assessment of mental status in the intervention phase when compared to the baseline phase [Difference 1.6%; 95%CI: -0.52% to 3.7%; p=0.18] **(Supplemental File 6)**.

For peer review only

Table 1: Patient's assessment of vital signs on admission (n=439)

Vital Sign	Baseline n=200	Intervention n=239	P-Value
Respiratory rate	194(97%)	239(100%)	0.009
Oxygen Saturations	51(25.5%)	239(100%)	0.0005
Temperature	195(97.5%)	239(100%)	0.019
Heart rate	194(97%)	238(99.6%)	0.051
Systolic BP	196(98%)	239(100%)	0.042
Diastolic BP	196(98%)	239(100%)	0.042
Urine output	153(76.5%)	239(100%)	0.0005
Mental State	196(98%)	238(99.6%)	0.182
Fetal heart rate (in pregnant woman) *	80 (58%)	123 (100%)	0.0005

*For FHR the denominator is 138 in baseline and 123 in the intervention phase.

Baseline characteristics of women with suspected maternal sepsis: 242 women were identified by the FAST-M decision tool as fulfilling the criteria for suspected maternal infection, of which 65% (130/200) women were during the baseline and 46.9% (112/239) during the intervention phase. Out of 242 women with maternal infections, 138 (57%) were identified with suspected maternal sepsis. Sixty of 138 (46.2%) were during the baseline phase and 78 of 112 (69.6%) were observed during the intervention phase. The characteristics of the patients with suspected maternal sepsis and various laboratory investigations carried out for the identification of source of infection are presented in **(Supplemental File 7&8)**.

Compliance with the FAST-M treatment bundle within 1 hour: Women with suspected maternal sepsis received all the components of the FAST-M treatment bundle, with the goal of treatment within 1 hour. The mean start time of the FAST-M treatment bundle was 30.67±15.18 minutes for fluids, 26.43±13.45 for antibiotics administration, 53±12.72 minutes for investigation of the source of infection and its treatment, 19.62±16.26 to assess the need to transfer high level and 54.43±10.1 for monitoring. Compliance improved following the intervention, as compared to the baseline [70.5%; 95%CI: 60.4% to 80.6%; p<0.01] ***(Table 2)***. Improvements in the management of sepsis cases were seen in all FAST-M bundle components, women

For peer review only - http://bmjopen.bmj.com/site/about/guidelines.xhtml

were more likely to get IV fluids [52.2%; 95%CI: 38% to 66.3% ; p<0.01], antibiotics [24.5%; 95%CI: 12.3% to 36.6% ;p<0.001], source identification [27.8%; 95%CI: 15.3% to 40.3%; p<0.01], assessment of the need to transfer required to higher level of care [65.3%; 95%CI: 52.8% to 77.6% ;p<0.01], and ongoing monitoring [63.3% ; 95%CI: 50.3% to 76.4%; p<0.01] within an hour of suspected sepsis identification compared to the baseline phase **(Figure 3)**. The improvements in the management of women with suspected maternal sepsis were maintained throughout the intervention, with no significant decline in performance over time (P trend value >0.05) **(Figure 4)**.

For peer review only

Table 2: Completion of FAST-M bundle within 1 hour of recognition of maternal sepsis

	Baseline (n=60)	Intervention FAST-M (n=78)	Intervention over time				P-Value	P-trend
			Month 1 (n=29)	Month 2 (n=18)	Month 3 (n=17)	Month 4 (n=14)		
**Total Bundle**	**0(0%)**	**55(70.5%)**	**20(69%)**	**9(50%)**	**15(88.2%)**	**11(78.6%)**	**0.0005**	**0.2069**
Fluids	21(35%)	68(87.2%)	24(82.8%)	17(94.4%)	15(88.2%)	12(85.7%)	0.0005	0.7516
Antibiotics	43(71.7%)	75(96.2%)	27(93.1%)	18(100%)	16(94.1%)	14(100%)	0.0001	0.3979
Source identification	41(68.3%)	75(96.2%)	28(96.6%)	17(94.4%)	17(100%)	13(92.9%)	0.0005	0.8405
Assessment of need to transfer high level	7(11.7%)	60(76.9%)	22(75.9%)	10(55.6%)	16(94.1%)	12(85.7%)	0.0005	0.1739
Monitoring	12(20%)	65(83.3%)	24(82.8%)	10(55.6%)	17(100%)	14(100%)	0.0005	0.0384

Maternal Outcomes: The facility data shows, 80 (28.6%) deaths due to maternal sepsis out of total 279 total maternal deaths recorded from the period of January to December 2021. **(Supplemental File 9)**. A 9.4% decline in the sepsis related maternal mortality was observed: 52/159 (32.7%) in the month of January-June and 28/120 (23.3%) during the July-December 2021 (p-value=0.087).

DISCUSSION

Main Findings:

The implementation of the FAST-M intervention was feasible, and effective in early identification, and management of maternal sepsis at the study setting. There was high compliance with all monitoring components after the intervention, with significant increase in compliance with completion of the MEOWS charts. Following the intervention, women with suspected sepsis were more likely to receive all of the critical early interventions needed to treat maternal sepsis. There was high and sustained compliance during the post-interventional observation period with completion of the FAST-M treatment bundle within an hour once maternal sepsis was suspected.

Interpretation:

The use of the MEOWS chart has been related to improved health outcomes and has been shown to predict severe maternal morbidity and mortality. Though there is limited evidence on its feasibility and impact on outcomes in low resource settings.[27] The present study confirms the applicability of the MEOWS chart in a low resource setting in Pakistan.. In the present study, we found that with the combined use of the MEOWS chart along with health care provider training, resulted in improvement in the monitoring of vital signs after the implementation of the FAST-M intervention.

In low-resource settings, the lack of screening processes and resources can be an obstacle to the early detection and treatment of maternal sepsis. [28,29] It was observed, that whilst the Royal College of Obstetricians and Gynaecologist guidelines (RCOG) were recommended for patient management and treatment at the LUMHS hospital, there was no standardised chart for the recording of patients' vital signs.

The MEOWS chart used in the Malawian settings,[21] was adapted before its implementation in Pakistan based on health care providers' suggestions during the adaptation phase of the study. Consequently, the health care providers were trained on an adapted MEOWS chart for regular recording of a full set of vital signs to screen patients who are at risk of developing sepsis, including the recording of oxygen saturations.

For peer review only

Providers showed a large improvement in the reliability of routine oxygen saturation recording following the intervention, so this component was shown to be a feasible addition in the Pakistan context.

Early detection of sepsis patients is critical for prompt treatment and improved maternal outcomes. [3,30,31] Research indicates that early warning scores accompanied by a clear escalation policy guide the healthcare providers on the need for additional action to optimise patient care.[31,32] The decision tool used guided the health care providers on when to proceed with treating the patient as having suspected sepsis and initiating the FAST-M bundle.[33,34] As noted earlier, the FAST-M screening tools in Malawi facilitated the identification of maternal sepsis patients and guided health care staff to escalate management plans.[21] The present study shows that it was also true in the Pakistan context where the FAST-M decision tool along with the MEOWS chart guided health care providers in appropriate screening and identification of women with suspected sepsis and assisted in making timely decisions for such cases.

The study was not powered to demonstrate reductions in maternal mortality or severe morbidity, but due to the critical importance of this outcome it was pre-specified as a secondary outcome. Following intervention, we observed a 9.4% decline in mortality of maternal sepsis patients), which suggests that the early identification of the sepsis patients and their timely management may as hoped lead to improved clinical outcomes. This finding is consistent with evidence from high income countries where the use of sepsis care bundles for the immediate treatment of sepsis has been demonstrated in large observational and interventional studies to reduce mortality and improve patient's condition.[13, 35] It's been reported that each part of the care bundle should be completed to reach the desired result, and failure to complete any of the components may have an impact on the overall patient outcome.[32] In the Malawi study, the FAST-M bundle implementation did not demonstrate a reduction in maternal mortality, [21] so our mortality reduction finding in Pakistan is particularly important.

Fluid administration is considered an important part of early sepsis management. [20,36] We observed a significant improvement in timely fluid administration once the FAST-M bundle was implemented. The restoration of organ perfusion is an important goal of maternal sepsis treatment and fluid resuscitation

For peer review only - http://bmjopen.bmj.com/site/about/guidelines.xhtml

should seek to restore organ perfusion while maintaining hemodynamic stability.[32] However, the benefits and risks of intensive early goal-guided fluid resuscitation have been the subject of intensive research.[33-36] The bundle care tools we used guided health care providers with a recommended fluid administration approach and to cautiously reassess the patient's hemodynamic condition between fluid boluses, through clinical examination and monitoring of the patient's vital signs. This sought to avoid overloading the patient which can actually worsen sepsis outcomes. [37,38] Higher volume of fluids in patients with sepsis and septic shock has been associated with harm, although a meta-analysis showed that the quality of evidence was very low [38]. The CLASSIC pilot study showed that restrictive fluid therapy was feasible in the ICU setting and it improved the mortality outcomes in the restrictive group, although not significantly [39].

Fluid management in patients with maternal sepsis and pre-eclampsia is challenging due to the increased risk of pulmonary oedema and must therefore be particularly cautiously managed by healthcare professionals.[32] During the FAST-M implementation, clear teaching and guidance were provided to the health care providers for proper fluid administration in pre-eclamptic or eclamptic patients.

Antibiotics are considered the cornerstone of sepsis treatment, and studies have shown that timely administration of antibiotics improves patient outcomes and reduces mortality.[40,41] The risk of death from sepsis increases with each hour of antibiotic delay (OR 1.04 per hour; 95 percent CI 1.03–1.06; p 0.001).[40,41] As a result, empiric broad-spectrum antibiotics should be given as early as possible, and it is normally suggested that clinicians should aim to provide this where possible within 1 hour of sepsis detection.[42] In Malawi, 42.3% improvement was seen in intravenous antibiotics administration within an hour of sepsis detection, from a relatively low baseline performance of 25%.[21] It is possible that the 24.5 percent increase in the present study in timely initiation of antibiotic administration may have led to improved clinical outcomes of women with suspected maternal sepsis.

For peer review only

Source identification was improved by 27.8% after FAST-M implementation in the study setting. Similar findings were reported in Malawi, where source identification improved by 18.2% within an hour of sepsis detection after FAST-M intervention.[21]

In the baseline phase, it was observed that clinicians had reservations to advise additional tests to identify the source of infection due to the logistics of undertaking these investigations in the facility and potential additional costs to be incurred by the patients. Considering this, the health care providers were given guidance during the training sessions regarding the significance of further investigations if the source is not initially clear with the clinical history and examination. [32] Consequently, an increase was seen in measurements of blood cultures, imaging (abdominal, and chest), urine and other blood tests in the intervention phase **(Supplemental File 8)**.

Amongst patients receiving treatment for maternal sepsis, the need for transfer to a higher level of care should be considered, and the decision on a patient's transfer made by a senior clinician as part of their review.[32] In Malawi, 43.9% improvement was seen in the consideration of transfer to the high level of care after the FAST-M intervention implementation.[21] During the training program, the importance of assessment of a patient for transfer within one hour of recognition of sepsis was highlighted, and emphasis was made on expediting the process if their vital signs were not improving after initial sepsis treatment. Following this, the assessment of need to transfer was improved by 65.3% within an hour of sepsis detection in the intervention period.

Overall, following the FAST-M intervention, more women were monitored and screened for maternal sepsis, and the complete FAST-M treatment bundle was administered within an hour in 70.5% of women with suspected sepsis. The study findings demonstrate that the FAST-M intervention was feasible and improved the quality of care and clinical outcomes when implemented in the context of a large government hospital in Pakistan. FAST-M has the potential to be used as an integrated approach for early recognition and management of maternal sepsis in low-resource health settings of Pakistan and other LMIC's. However,

For peer review only - http://bmjopen.bmj.com/site/about/guidelines.xhtml

as we found when translating the FAST-M tools from Malawi to Pakistan some modifications in the intervention were needed to ensure they were optimised for the local context and available resources.

Strengths and limitations:

The strength of our research is that it's the first feasibility study conducted for the assessment of a maternal sepsis care bundle in a low-resource setting within Pakistan. This approach provided the opportunity to analyse practical, and operational factors for introducing the FAST-M intervention in the local setting. The findings of this study can lead to optimization of the bundle prior to the large-scale intervention trial. Also, this study adds further weight to Malawi work demonstrating the feasibility of the FAST-M bundle in different low resource settings.

However, the study has several limitations. Our research design does not account for possible temporal effects, hence is exposed to possible selection and reporting bias.[43] Although a before and after design couldn't identify the cause-and-effect relationship, this was the practical approach for determining the feasibility of the FAST-M intervention locally.

This study was conducted at one site only i.e., LUMHS which is a tertiary hospital that was comparatively better equipped than basic health units (BHUs) in the country to implement any care bundle approach. Hence, the intervention will next be implemented in other low-resource settings including BHU's across Pakistan. A large multi-country randomised trial is also underway to more robustly ascertain the effectiveness of the FAST-M bundle to improve maternal sepsis care and outcomes in LMICs [44]

Although the intervention was found to be feasible, we recognise that the intervention period was short. A further assessment period is required to examine how well the FAST-M intervention is sustained and if it becomes normalised as a routine part of clinical care in the longer term.. This additional information has been collected via an embedded qualitative study and will be reported separately.

For peer review only

Additionally, the high influx of patients and shortage of health care staff in LUMHS could have led to irregular monitoring which might have resulted in limited screening of the patients. Some of the women with suspected sepsis may have been categorised as maternal infection cases, that might have restricted the ability to demonstrate the differences between intervention and baseline phase.

Some of the referred patients in the baseline phase were observed to be already on fluid and antibiotics before admission to the hospital, which could have limited the ability of a before and after comparison, though it was only 9.4% (13/138) of patients in the baseline phase who were noted to have already received fluids and antibiotics. Additionally, the health care providers might have been more compliant with the FAST-M intervention, and the outcomes of this study therefore better than one would see if such an intervention was rolled out at scale due to the close monitoring of the site during the conduct of the study.

CONCLUSION

Implementation of the FAST-M intervention was feasible and significantly improved the quality of maternal sepsis care, including enhanced early identification and management of maternal sepsis. The long-term vision is that the intervention will be trialled in other settings across Pakistan, based on the outcomes of this feasibility study, which will help to reduce the high rate of maternal deaths caused by sepsis in the country.

Acknowledgments: We would like to acknowledge the healthcare providers from the LUMHS hospital Dr Prince Akash, Dr Aqsa, Dr Batool, Dr Sana, Dr Qurat ul ain, Dr. Sahiba, and staff nurse Tomsina who served as FAST-M champions and actively took on their role in implementing the FAST-M bundle intervention. We would also like to acknowledge Ms. Ruqaiya Bano for data collection of this study and Ms Maliha Fazal for editing the manuscript.

Contributors: DL and SI designed the study and its processes. RS, FP, NM and SK provided support in data collection. AR, SI GK, BK, AJ and DM carried out the data analysis and interpretation. SI, GK, AR, and BK took the lead in writing the manuscript. SI takes responsibility for the overall content as the guarantor. AC, DL, CD, JC, LS, RS, RB, SSH SS, FP reviewed the manuscript and provided feedback on the article.

For peer review only - http://bmjopen.bmj.com/site/about/guidelines.xhtml

Funding: This research was commissioned by the National Institute for Health Research (NIHR). Award Ref: NIHR300808 Host: University of Liverpool. The views expressed in this paper are those of the authors and not necessarily those of the NIHR or the Department of Health and Social Care. The project was also supported by PhD award of WHO Eastern Mediterranean Hub (AKU) of HRP Alliance, for Research Capacity Development in Sexual and Reproductive Health, University of Birmingham Alumni funds, and Aga Khan University Research Funds for Faculty.

Competing interests: Declared none.

Patient and public involvement: Patients were not involved in planning the design of this study. The FAST-M intervention was implemented on the enrolled patients however, they were not invited to comment on the study design, interpret the results or contribute to writing or editing of this document for accuracy of study findings. Hospital staff participated in the implementation but were not involved in the analysis of findings for accuracy and readability.

Patient consent for publication: Not applicable.

Ethics approval: Ethical approval for this study was obtained from the LUMHS hospital [REC/-886, 4-87], Aga Khan University Ethical Review Committee [2019-2061-7102], and National Bioethics Committee [515/20/].

Provenance and peer review: Not commissioned; externally peer reviewed.

Data availability statement: The datasets were collected and analyzed and can be made available from the corresponding author on reasonable request.

For peer review only

ORCID iDs:

SI: 0000-0002-8391-8559

GK: 0000-0003-3224-730X

RS: 0000-0002-8015-5744

AR: 0000-0003-3970-8236

BK: 0000-0002-8141-9203

FP: 0000-0002-7820-6565

SK: 0000-0002-9655-378X

NM: 0000-0002-5174-8671

AJ: 0000-0002-6674-8543

DM:0000-0002-3396-3087

SS: 0000-0003-1301-5239

JC: 0000-0002-0967-7320

CD: 0000-0002-4792-9496

RB: 0000-0002-7377-9524

LS: 0000-0002-7694-3561

SS: 0000-0002-6797-8631

AC: 0000-0002-3261-9807

DL: 0000-0002-7878-232

For peer review only

References:

1. Bonet M, Pileggi V, Rijken MJ, Coomarasamy A, et al. Towards a consensus definition of maternal sepsis: results of a systematic review and expert consultation. *Reprod Health* 2017;14:67.
2. Say L, Chou D, Gemmill A, et al. Global causes of maternal death: a WHO systematic analysis. *Lancet Global Health*. 2014 Jun 1;2(6):e323-33.
3. Kourtis AP, Read JS, Jamieson DJ. Pregnancy and infection. *N Engl J Med*. 2014;370(23):2211–8.
4. Vasudevan R. Urinary tract infection: an overview of the infection and the associated risk factors. *J Microbiol Exp*. 2014;1(2):42–54.
5. Bonet M, Brizuela V, Abalos E, et al. Frequency and management of maternal infection in health facilities in 52 countries (GLOSS): a 1-week inception cohort study. *Lancet Global Health.* 2020 May 1;8(5):e661-71
6. Vos T, Lim SS, Abbafati C, et al. Global burden of 369 diseases and injuries in 204 countries and territories, 1990–2019: a systematic analysis for the Global Burden 2019. *Lancet*. 2020;396(10258):1204–22
7. Rudd KE, Johnson SC, Agesa KM, et al. Global, regional, and national sepsis incidence and mortality, 1990–2017: analysis for the Global Burden of Disease Study. *Lancet.*2020; 395: 200–11.
8. World Health Organization. Trends in maternal mortality: 1990 to 2015: estimates from WHO, UNICEF, UNFPA, World Bank Group and the United Nations Population Division: executive summary. (apps.who.int/iris/handle/10665/193994) Accessed on 1st Sept 2022
9. Hanif M, Khalid S, Rasul A, et al. Maternal mortality in rural areas of Pakistan: challenges and prospects. *Rural Heal*. 2021 Jun 7;27:1040-7.
10. Fikree FF, Ali T, Durocher JM, et al. Health service utilization for perceived postpartum morbidity among poor women living in Karachi. *Social science & medicine*. 2004 Aug 1;59(4):681-94.
11. National Institute of Population Studies (NIPS), ICF International (2013) Pakistan Demographic and Health Survey 2012-13. NIPS and ICF International, Islamabad and Calverton. - References - Scientific Research Publishing (www.scirp.org/(S(351jmbntvnsjt1aadkposzje))/reference/referencespapers.aspx?referenceid=2849234) Accessed on 1st Sept 2022
12. Reinhart K, Daniels R, Kissoon N, et al. Recognizing sepsis as a global health priority – A WHO resolution. *N Engl J Med* 2017;377:414–7.

For peer review only - http://bmjopen.bmj.com/site/about/guidelines.xhtml

13. Damiani E, Donati A, Serafini G, et al. Effect of performance improvement programs on compliance with sepsis bundles and mortality: a systematic review and meta-analysis of observational studies. *PloS one*. 2015 May 6;10(5):e0125827.

14. Levy MM, Dellinger RP, Townsend SR, et al. The Surviving Sepsis Campaign: results of an international guideline-based performance improvement program targeting severe sepsis. *Crit Care Med* 2010;38(2):367–74.

15. Evans L, Rhodes A, Alhazzani W, et al. Surviving sepsis campaign: international guidelines for management of sepsis and septic shock 2021. *Int care med*. 2021 Nov;47(11):1181-247.

16. Nutbeam T, Daniels R, Keep J, UK Sepsis Trust. Toolkit: Emergency Department management of Sepsis in adults and young people over 12 years- 2016. The Royal College of Emergency Medicine; National Institute for Health and Care Excellence (NICE); 2016.

17. Abdu M, Wilson A, Mhango C, et al. Resource availability for the management of maternal sepsis in Malawi, other low-income countries, and lower-middle-income countries. *Int J Gynecol Obstet* 2018;140:175–83.

18. Baelani I, Jochberger S, Laimer T, et al. Availability of critical care resources to treat patients with severe sepsis or septic shock in Africa: a self-reported, continent wide survey of anaesthesia providers. *Crit Care* 2011;15:R10.

19. Bataar O, Lundeg G, Tsenddorj G, et al. Nationwide survey on resource availability for implementing current sepsis guidelines in Mongolia. *Bull World Health Organ* 2010;88:839–46.

20. Lissauer D, Cheshire J, Dunlop C, et al. Development of the FAST-M maternal sepsis bundle for use in low-resource settings: a modified Delphi process. *BJOG-INT J OBS*. 2020 Feb;127(3):416-23

21. Cheshire J, Jones L, Munthali L, et al. The FAST-M complex intervention for the detection and management of maternal sepsis in low-resource settings: a multi-site evaluation. *BJOG-INT J OBS*. 2021 Feb 4.

22. Reinhart K, Kissoon N. Sepsis Guidelines for Pakistan. *Anaesth Pain & intensive Care*. 2015 Apr 1;19(2):105-7.

23. Ahmed SI, Sikandar R, Barolia R, et al.Evaluation of the feasibility of the FAST-M maternal sepsis intervention in Pakistan: a protocol. *Pilot and feasibility studies*. 2022 Dec;8(1):1-2.

For peer review only

24. Ahmed SI, Khowaja BMH, Barolia R, et al. Adapting the FAST-M maternal sepsis intervention for implementation in Pakistan: a qualitative exploratory study. *BMJ Open* 2022;12:e059273.

25. Umar A, Ameh CA, Muriithi F, et al. Early warning systems in obstetrics: a systematic literature review. *PLoS One* 2019;14: e0217864.

26. Downey CL, Tahir W, Randell R, et al. Strengths and limitations of early warning scores: A systematic review and narrative synthesis.*I nt J Nurs Stud* 2017;76:106–19.

27. Pakenham-Walsh N, Bukachi F. Information needs of health care workers in developing countries: a literature review with a focus on Africa. *Hum Resour Health* 2009;7:30.

28. Papali A, McCurdy MT, Calvello EJB. A ‘three delays’ model for severe sepsis in resource-limited countries. *J Crit Care* 2015;30:861.e9– 861.e14.

29. Gatewood MO, Wemple M, Greco S, et al. A quality improvement project to improve early sepsis care in the emergency department. *BMJ Qual Saf* 2015;24:787–95.

30. Hayden GE, Tuuri RE, Scott R, et al. Triage sepsis alert and sepsis protocol lower times to fluids and antibiotics in the ED. *Am J Emerg Med* 2016;34:1–9.

31. Seymour CW, Gesten F, Prescott HC, et al. Time to treatment and mortality during mandated emergency care for sepsis. *N Engl J Med* 2017;376:2235–44.

32. Cheshire J, Dunlop C, et al, Glob. libr. women's med., ISSN: 1756-2228; DOI 10.3843/GLOWM.413113

33. The Uk Sepsis Trust. Clinical Professional Resources. 2018.

34. Suleiman I, Vousden N, Shennan AH. Recognition and Management of Maternal Sepsis in Low and Middle-Income Countries: What do we know and where are the Gaps? Open access. *Glob Womens Heal* 2018;1(1):1–5.

35. Rivers E, Nguyen B, Havstad S, et al. Early Goal-Directed Therapy in the Treatment of Severe Sepsis and Septic Shock.*N Engl J Med* 2001;345(19):1368–77.

36. Mouncey PR, Osborn TM, Power GS, et al. Trial of Early, Goal-Directed Resuscitation for Septic Shock. *N Engl J Med* 2015;372(14):1301–11.

37. RISE Investigators, ANZICS Clinical Trials Group, Peake SL, Delaney A, Bailey M, Bellomo R,e t al. Goal-Directed Resuscitation for Patients with Early Septic Shock. *N Engl J Med* 2014;371(16):1496–506.

For peer review only

38. Angus DC, Yealy DM, Kellum JA. Protocol-based care for early septic shock. *N Engl J Med*. 2014 Jul 1;371(4):386-.

39. Meyhoff TS, Hjortrup PB, Wetterslev J, Sivapalan P, Laake JH, Cronhjort M, Jakob SM, Cecconi M, Nalos M, Ostermann M, Malbrain M. Restriction of intravenous fluid in ICU patients with septic shock. New England Journal of Medicine. 2022 Jun 30;386(26):2459-70.

40. de Oliveira FSV, Freitas FGR, Ferreira EM, *et al.* Positive fluid balance as a prognostic factor for mortality and acute kidney injury in severe sepsis and septic shock. *J Crit Care* 2015;30(1):97–101.

41. Liu VX, Fielding-Singh V, Greene JD, et al. The Timing of Early Antibiotics and Hospital Mortality in Sepsis.*Am J Respir Crit Care Med* 2017;196(7):856–63.

42. Rhodes A, Evans LE, Alhazzani W, et al. Surviving Sepsis Campaign: International Guidelines for Management of Sepsis and Septic Shock: 2016. *Int Care Med* 2017;43(3):304–77.

43. Songer T. Before and after studies in injury research. Aufgerufen am. 2008; 13:2015.

44. Lindiwe. 2020 MLW Annual report is released! [Internet]. Malawi Liverpool Wellcome Trust Clinical Research Program. 2021 [cited 2023Feb2]. Available from: https://www.mlw.mw/2020-mlw-annual-report-is-released/

Figure Legends

Figure 1: Flow of participants through the study

Figure 2: Components of FAST-M Intervention

Figure 3: Completion of FAST-M bundle within 1 hour of recognition of maternal sepsis

Figure 4: Completion of FAST-M bundle within 1 hour of recognition of maternal sepsis according to time point

For peer review only

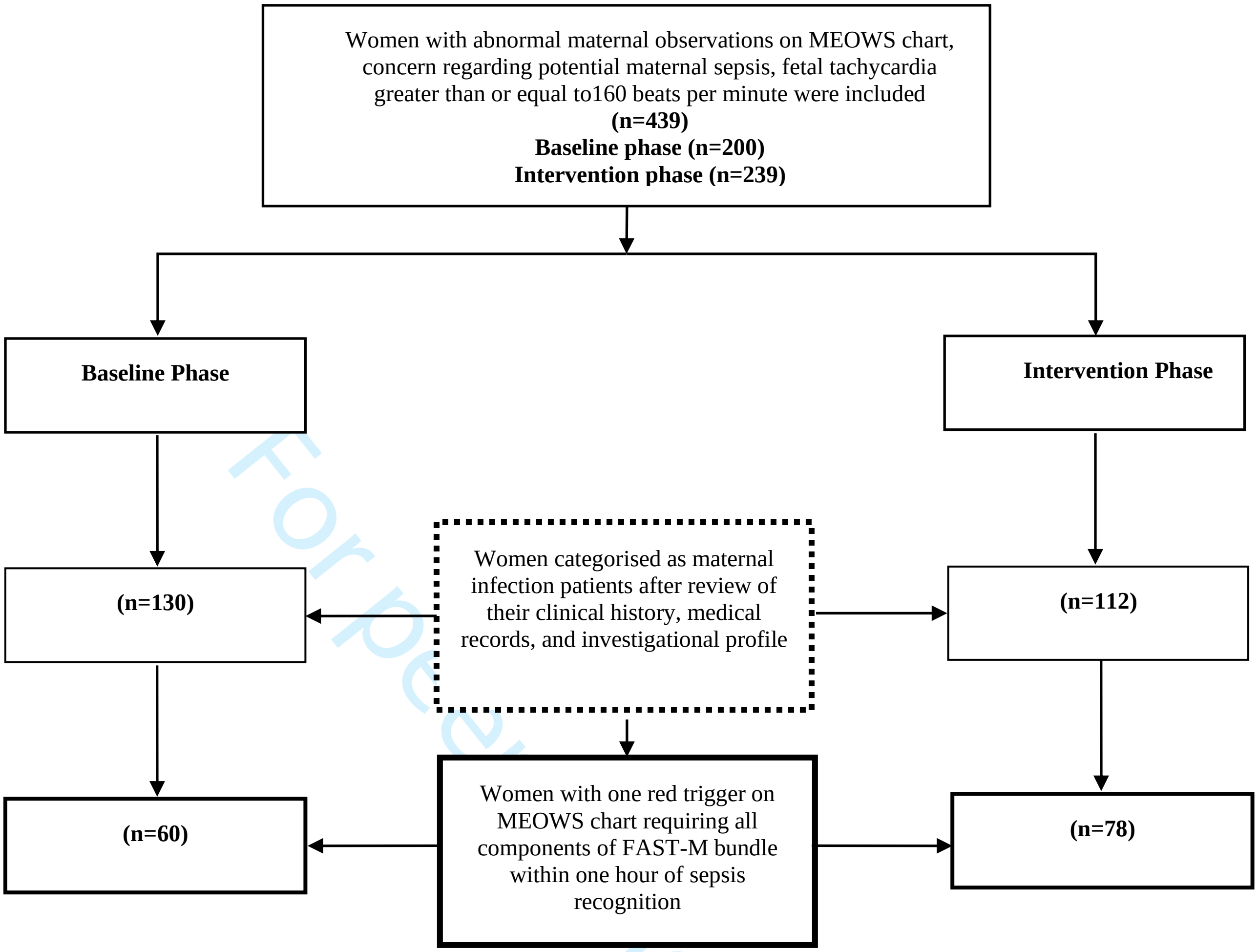

Figure 1: Flow of participants through the study

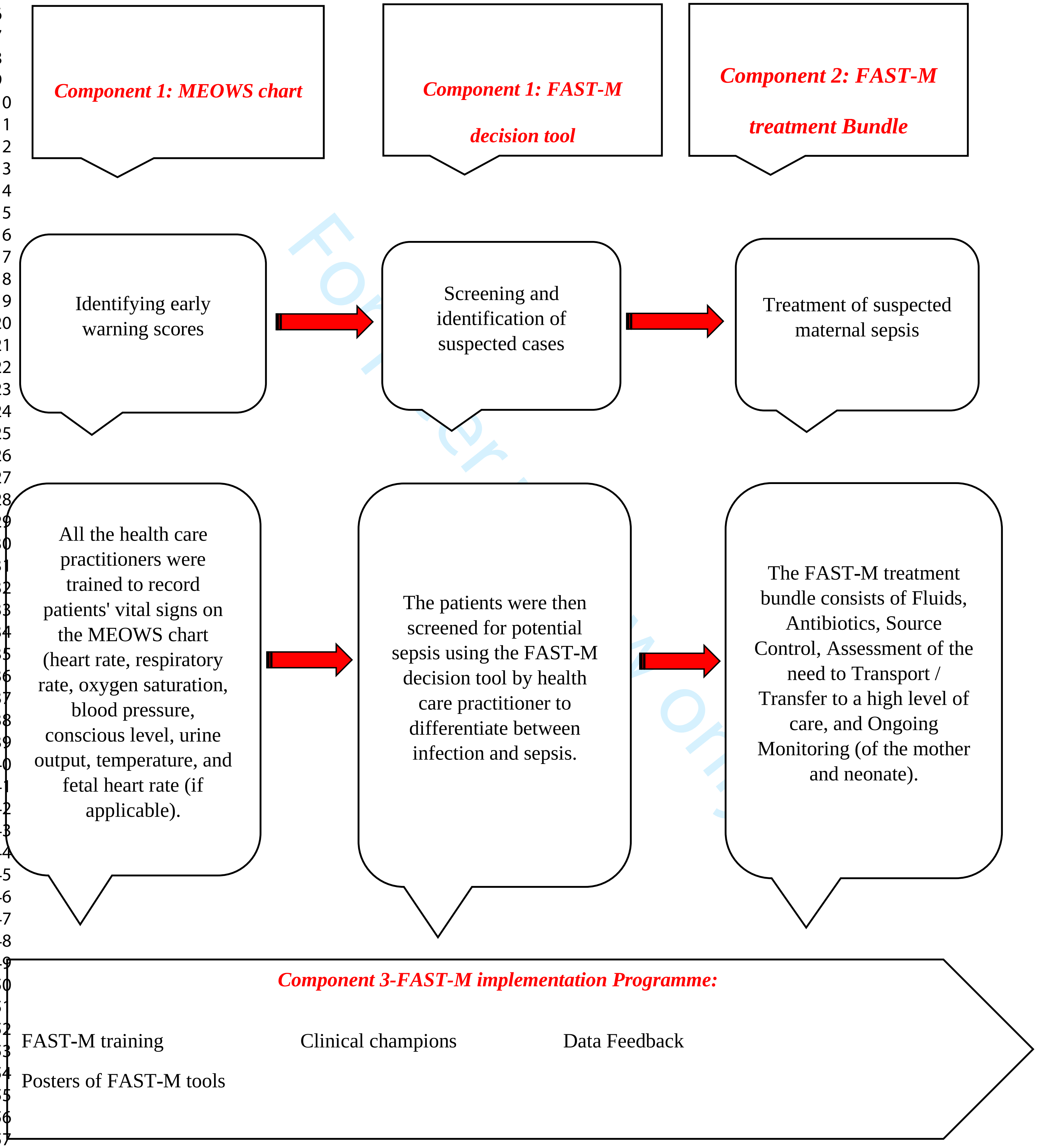

Figure 2: Components of FAST-M Intervention

For peer review only - http://bmjopen.bmj.com/site/about/guidelines.xhtml

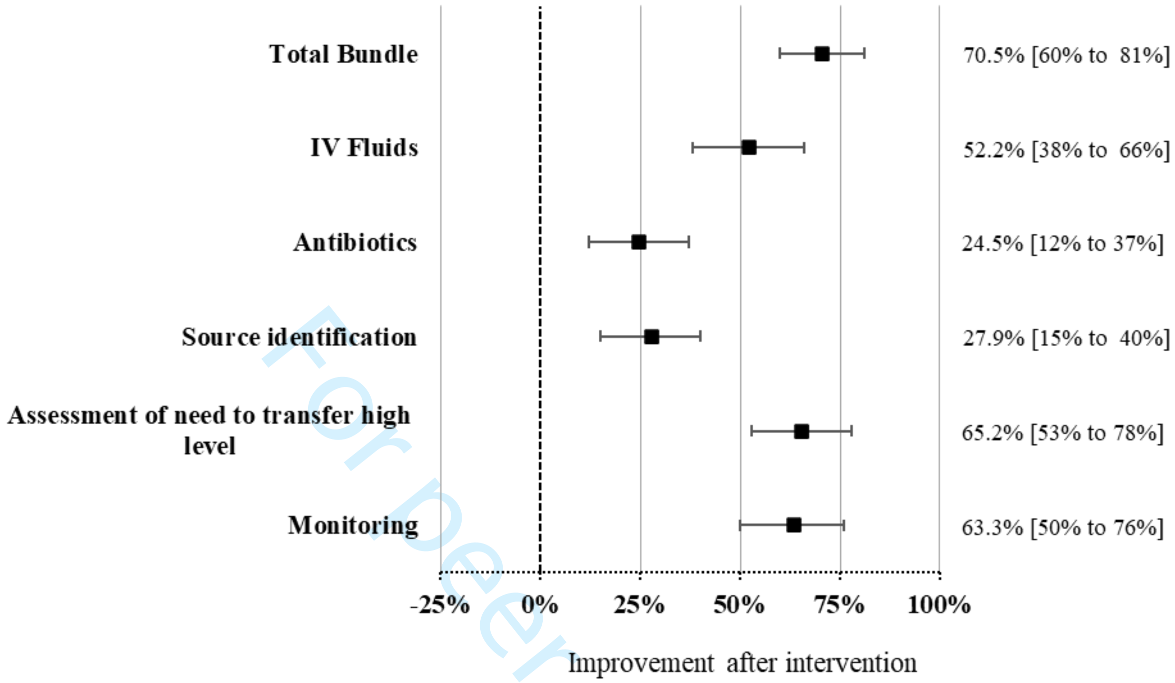

Figure 3: Completion of FAST-M bundle within 1 hour of recognition of maternal sepsis

For peer review only

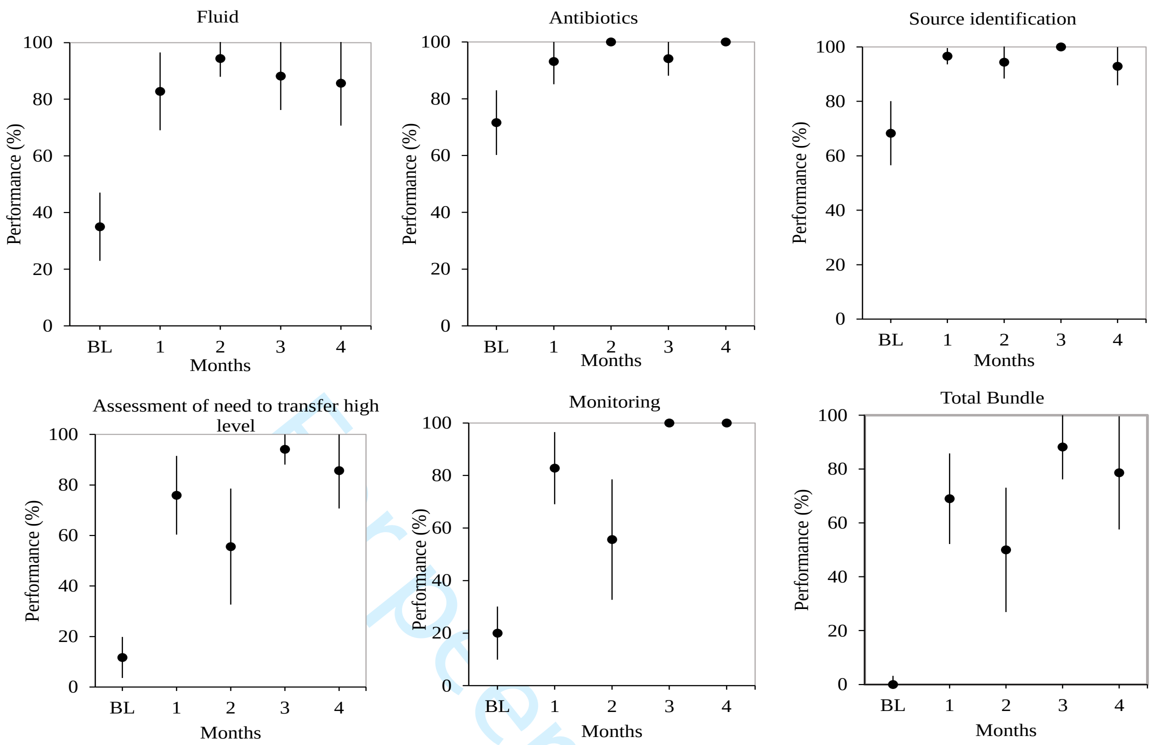

Figure 4: Completion of FAST-M bundle within 1 hour of recognition of maternal sepsis according to time point

MODIFIED EARLY OBSTETRIC WARNING CHART (MEOWS CHART)

FAST-M

Contact clinical decision maker if patient triggers **ONE RED** or **TWO YELLOW** flags at any one time.

Patient		Patient ID		DOB/Age	

WRITE **VALUES** IN BOXES PROVIDED

		Date															
		Time															
		Initials															
**Respiratory rate** (breaths per minute)	25 or more	RED															
	21 - 24	YELLOW															
	11 - 20	NORMAL															
	10 or less	RED															
**Oxygen saturations** (%)	95 or more	NORMAL															
	94 or less OR needing oxygen	RED															
**Temperature** (°c)	38 or more	YELLOW															
	36.0 to 37.9	NORMAL															
	35.9 or less	YELLOW															
**Heart rate** (beats per minute)	120 or more	RED															
	100 - 119	YELLOW															
	50 - 99	NORMAL															
	40 - 49	YELLOW															
	39 or less	RED															
**Systolic blood pressure** (mmHg)	160 or more	RED															
	140 -159	YELLOW															
	100 -139	NORMAL															
	90 - 99	YELLOW															
	89 or less	RED															
**Diastolic blood pressure** (mmHg)	110 or more	RED															
	90 -109	YELLOW															
	40 - 89	NORMAL															
	39 or less	RED															
**Urine output** Hours since patient passed urine (tick box)	12 hours or less	NORMAL															
	12 - 18 hours	YELLOW															
	18 hours or more OR less than 0.5 ml/kg/hour	RED															
**Mental State** (tick box)	Alert	NORMAL															
	Not Alert	RED															
**Looks unwell** (tick box)	No	NORMAL															
	Yes	YELLOW															
**TOTAL YELLOW FLAGS**																	
**TOTAL RED FLAGS**																	
ACTION TAKEN (IF REQUIRED) Yes (Y) / No (N)																	

ACT NOW if patient triggers ONE RED or TWO YELLOW flags at any time. Escalate to clinical decision maker and start FAST-M decision tool.

UNITED AGAINST MATERNAL SEPSIS

THINK SEPSIS, ACT FAST-M

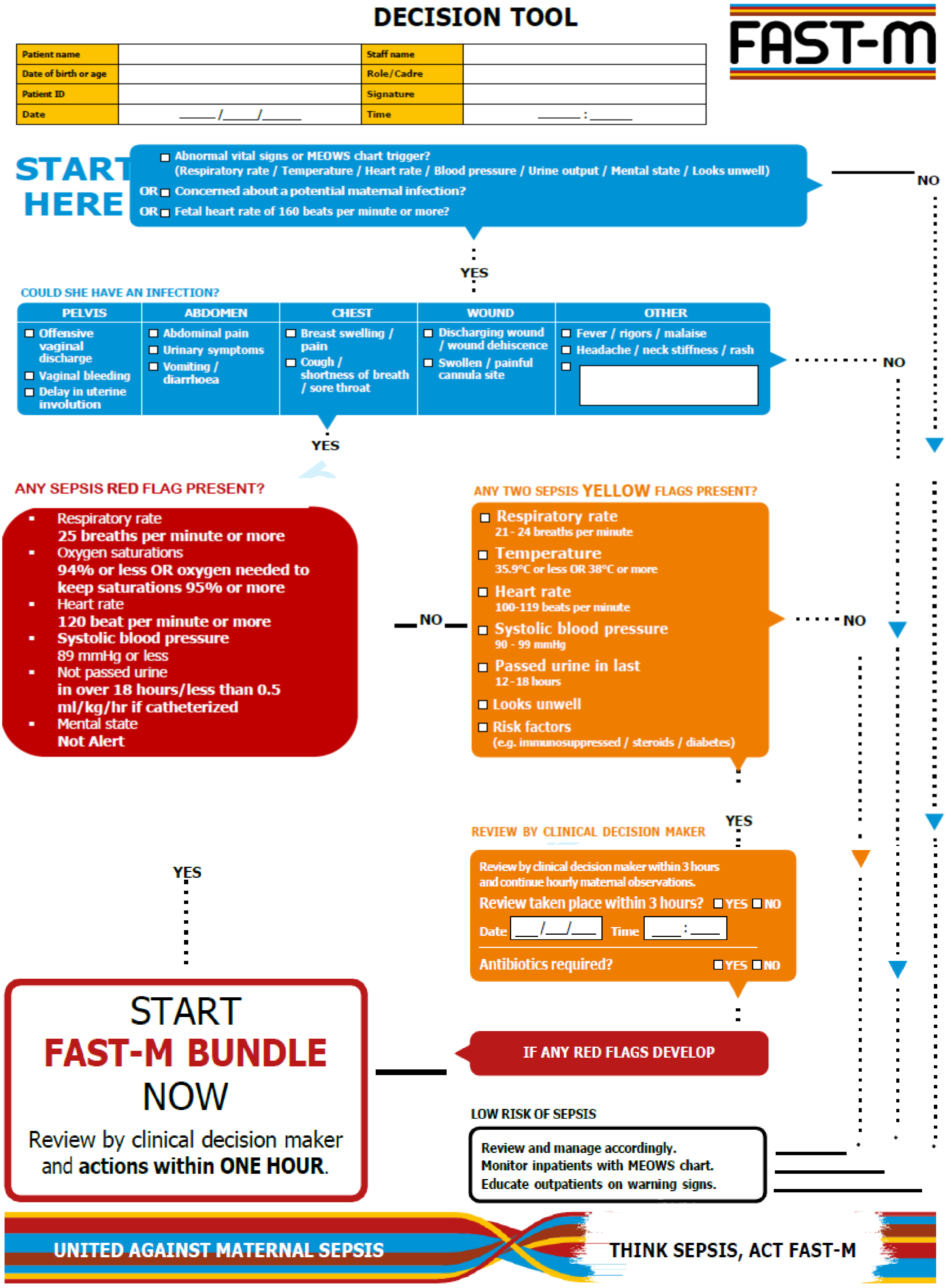
DECISION TOOL
FAST-M
Patient name
Date of birth or age
Patient ID
Date ____/____/____
Staff name
Role/Cadre
Signature
Time ____:____
START HERE
Abnormal vital signs or MEOWS chart trigger?
(Respiratory rate / Temperature / Heart rate / Blood pressure / Urine output / Mental state / Looks unwell)
OR Concerned about a potential maternal infection?
OR Fetal heart rate of 160 beats per minute or more?
NO
YES
COULD SHE HAVE AN INFECTION?
PELVIS
Offensive vaginal discharge
Vaginal bleeding
Delay in uterine involution
ABDOMEN
Abdominal pain
Urinary symptoms
Vomiting / diarrhoea
CHEST
Breast swelling / pain
Cough / shortness of breath / sore throat
WOUND
Discharging wound / wound dehiscence
Swollen / painful cannula site
OTHER
Fever / rigors / malaise
Headache / neck stiffness / rash
NO
YES
ANY SEPSIS RED FLAG PRESENT?
Respiratory rate
25 breaths per minute or more
Oxygen saturations
94% or less OR oxygen needed to keep saturations 95% or more
Heart rate
120 beat per minute or more
Systolic blood pressure
89 mmHg or less
Not passed urine
in over 18 hours/less than 0.5 ml/kg/hr if catheterized
Mental state
Not Alert
NO
ANY TWO SEPSIS YELLOW FLAGS PRESENT?
Respiratory rate
21 - 24 breaths per minute
Temperature
35.9°C or less OR 38°C or more
Heart rate
100-119 beats per minute
Systolic blood pressure
90 - 99 mmHg
Passed urine in last
12 - 18 hours
Looks unwell
Risk factors
(e.g. immunosuppressed / steroids / diabetes)
NO
YES
REVIEW BY CLINICAL DECISION MAKER
Review by clinical decision maker within 3 hours and continue hourly maternal observations.
Review taken place within 3 hours? YES NO
Date ___/___/___ Time ___:___
Antibiotics required? YES NO
YES
START
FAST-M BUNDLE
NOW
Review by clinical decision maker and actions within ONE HOUR.
IF ANY RED FLAGS DEVELOP
LOW RISK OF SEPSIS
Review and manage accordingly.
Monitor inpatients with MEOWS chart.
Educate outpatients on warning signs.
UNITED AGAINST MATERNAL SEPSIS
THINK SEPSIS, ACT FAST-M

TREATMENT BUNDLE

FAST-M

REMEMBER TO COMPLETE THESE ACTIONS WITHIN ONE HOUR

Patient name		Staff name			
D.O.B or age		Role/Cadre			
Patient ID		Signature			
Date & time of red flag observation	__/__/__ __:__	Date & time bundle started	__/__/__ __:__	Date & time of review by clinical decision maker	__/__/__ __:__

F — FLUIDS (caution in pre-eclampsia, severe anaemia and pulmonary oedema)

Date	___/___/___	Time fluids initiated	___ : ___	Initials	
Details / reason not completed					

Give 500 ml crystalloid immediately. Repeat 500 ml boluses to a maximum of 30 ml/kg if hypotension persists.

A — ANTIBIOTICS

Date	___/___/___	Time started	___ : ___	Initials	
Details / reason not completed					

See antibiotic guidelines below

S — SOURCE – identify and treat the source of infection

Date	___/___/___	Time considered	___ : ___	Initials	
Details / reason not completed					

See source identification and treatment boxes below

T — TRANSPORT (to higher level hospital or location within hospital, if required)

Date & time transport considered	___/___/___ ___:___	Initials		Transport Required ☐ YES ☐ NO
Date & time transport requested	___/___/___ ___:___	Initials		☐ N/A
Date & time patient left facility	___/___/___ ___:___	Initials		
Destination				
Reason for any delay				

M — MONITORING (start MEOWS chart if not already started. Repeat observations every 30 minutes until otherwise decided by clinical decision maker)

Date & time monitoring commenced	___/___/___ ___ : ___	Details / reason not completed
Maternal / fetal monitoring should include	• Respiratory rate • Oxygen Saturations • Temperature • Heart rate • Blood pressure • Urine output • Mental state • Fetal heart rate	
Neonatal monitoring and review commenced	☐ YES ☐ NO ☐ N/A	

ANTIBIOTIC GUIDELINES

Insert local guidance here

Immediate treatment for Maternal Sepsis:

- Ceftriaxone 2 g IV once daily (if no IV access this can be given as 2 IM injections of 1 g in different sites).
- If possible intra-abdominal source add Metronidazole 500 mg IV three times daily or 400 mg PO three times daily.

If above antibiotic regime is not available then give:

- Tazobactam 4.5 g IV daily two time a day
- Meropenem 1 g IV daily two times a day

IDENTIFY THE SOURCE

Consider

- Clinical history
- Clinical examination
- Blood tests (if available) (FBC, U&Es, LFTs, CRP, clotting)
- Blood cultures
- HIV and Malaria testing
- Urine sample
- Swabs (wound, vagina, throat)
- Sputum sample
- Imaging (abdominal, chest)
- Lumbar puncture
- Other ___________

REMOVE / TREAT THE SOURCE

Consider

- Malaria treatment
- Consider delivery of baby
- Removal of retained products of conception
- Debridement of wound / drainage of collection
- Removal of infected cannula / line
- Hysterectomy
- Targeted antibiotics once source known

UNITED AGAINST MATERNAL SEPSIS   THINK SEPSIS, ACT FAST-M

FORM 1: FACILITY AUDIT

FACILITY ID [ ][ ] – [ ][ ][ ]

Facility ID e.g. DH, KH *Visit ID e.g. 001, 002 etc.*

Today's Date: d d – m m – y y y y Time: h h : m m

Date previous form completed (or if first Visit, date study opened at this site): d d – m m – y y y y

Are you collecting the data during? **Baseline** ☐ **Intervention** ☐

3. How many of the following **MATERNAL OUTCOMES** have you had since the last visit or if first time since last 6 months?

Maternal Outcome	Number
Maternal Sepsis	
Maternal Deaths	
Post-Partum haemorrhage (>1L)	
Ante-Partum haemorrhage (>50ml)	
Severe pre-eclampsia/eclampsia (>160/110 and >2+protein in urine)	
Blood transfusions	
Uterine rupture	
Patient with reduced conscious level	
Injury (e.g. uterine, bladder, bowel)	
Anaphylaxis	
Pulmonary oedema	
Return to theatre	
Admission to ICU/HDU	
Broken down wounds (e.g. c/s, perineal)	

4. How many of the following **NEONATAL OUTCOMES** have you had since the last visit?

Neonatal Outcome	Number
Live Births	
Neonatal deaths before discharge from hospital	
Babies requiring antibiotics	
Sill births	
Admission to nursery care	
Apgar score of less than 7 at 5 mins after birth	

For peer review only

FORM 1: FACILITY AUDIT

FACILITY ID ☐☐ – ☐☐☐

Facility ID e.g. DH, KH *Visit ID e.g. 001, 002 etc.*

5. How many of the following **RESOURCES** are available today?

**Resource**	**Amount**			
	BAGS			
**Type of IV Fluids:**	0	1-5	6-10	>10
A) 0.9% Saline	☐	☐	☐	☐
B) Ringers Lactate	☐	☐	☐	☐
C) 5% dextrose	☐	☐	☐	☐
D) 50% dextrose	☐	☐	☐	☐

	**Type of oral antibiotics**			
**Type of oral antibiotics**	0	1-5	6-10	>10
A) Amoxicillin	☐	☐	☐	☐
B) Augmentin	☐	☐	☐	☐
C) Cephalosporin (e.g. Cefalexin)	☐	☐	☐	☐
D) Cefixime	☐	☐	☐	☐
E) Ciprofloxacin	☐	☐	☐	☐
F) Doxycycline	☐	☐	☐	☐
G) Clindamycin	☐	☐	☐	☐
H) Chloramphenical	☐	☐	☐	☐
I) Erythromycin	☐	☐	☐	☐
J) Metronidazole	☐	☐	☐	☐
K) Other (please state):	☐	☐	☐	☐

	**VIALS**			
**Type of IM / IV antibiotics**	0	1-5	6-10	>10
A) Amoxicillin	☐	☐	☐	☐
B) Ampicillin	☐	☐	☐	☐
C) Benzyl-penicillin	☐	☐	☐	☐
D) Penicillin G	☐	☐	☐	☐
E) Cefazolin	☐	☐	☐	☐
F) Cephalosporin (e.g. ceftriazone etc.)	☐	☐	☐	☐
G) Chloramphenical	☐	☐	☐	☐
H) Ciprofloxacin	☐	☐	☐	☐
I) Clindamycin	☐	☐	☐	☐
J) Erythromycin	☐	☐	☐	☐
K) Gentamycin	☐	☐	☐	☐
L) Metronidazole	☐	☐	☐	☐
M) Vancomycin	☐	☐	☐	☐
N) Other (please state)	☐	☐	☐	☐

	KITS			
**Manual vacuum aspiration (MVA) kits ready for use:**	0	1-5	6-10	>10
	☐	☐	☐	☐

* Good availability defined as a supply that is unlikely to run out before the next anticipated delivery

For peer review only

FORM 1: FACILITY AUDIT

FACILITY ID ☐☐ – ☐☐☐

Facility ID e.g. DH, KH *Visit ID e.g. 001, 002 etc.*

Resource	Availability		
	Good *	Limited	None
Equipment for IV line (cannula, dressings etc)	☐	☐	☐
Malaria tests	☐	☐	☐
Syphilis test	☐	☐	☐
HIV test	☐	☐	☐
Functioning theatre and staff able to remove source of infection	☐	☐	☐
Working thermometers	☐	☐	☐
Working BP machines	☐	☐	☐
Working O2 saturation machines(Pulse Oximeters)	☐	☐	☐
Fetoscopes / Pinnards/ fetal stethoscopes	☐	☐	☐
Clocks/watches	☐	☐	☐
Spare batteries	☐	☐	☐
Wound swabs	☐	☐	☐
Corona virus swabs	☐	☐	☐
Urine dips	☐	☐	☐
Sputum cultures	☐	☐	☐
Blood cultures	☐	☐	☐
Pregnancy tests	☐	☐	☐
Haematology blood tests	☐	☐	☐
Biochemistry blood tests	☐	☐	☐
Corona virus tests	☐	☐	☐
Blood taking equipment	☐	☐	☐
Functioning radiology department	☐	☐	☐
Functioning ultrasound machine and person to scan	☐	☐	☐
Functioning theatre and staff able to remove source of infection	☐	☐	☐
Weighing scales	☐	☐	☐
Urinary catheters	☐	☐	☐
Gloves	☐	☐	☐
Aprons	☐	☐	☐

6. How many of the following **STAFF** is available TODAY?

Resource	Number
Doctors	
Medical Officers	
Medical Students	
Registered Nurse	
Nursing students	
Hospital and Ward Attendants	

For peer review only

FORM 1: FACILITY AUDIT

FACILITY ID [ ][ ] – [ ][ ][ ]

Facility ID e.g. DH, KH *Visit ID e.g. 001, 002 etc.*

7. Are the following **FACILITIES** in working order TODAY?

Resource	Yes	No
Does the ward have electricity?		
Does the ward have running water?		
Does the ward have soap?		
Does the ward have alcohol gel?		
Are the autoclaves working?		
Are the washing facilities for staff available?		
Are the washing facilities for patients available?		
Are flushable toilet facilities for staff available?		
Are flushable toilet facilities for patients available?		

* Good availability defined as a supply that is unlikely to run out before the next anticipated delivery

Completed by: ______________________

Role: ______________________

Signature: ______________________

Date: *DD* / *MMM* / *YYYY*

You must have signed the Site Signature & Delegation Log

For peer review only

FORM 2: PATIENT CARE AUDIT FORM

Patient ID Number: ☐☐ - ☐☐☐

Facility ID e.g. A01, A02 etc *Unique ID e.g. 001, 002 etc*

Baseline ☐ **or** Intervention ☐

Date of audit: d d - m m - y y y y   Time of audit: h h : m m

1. Clinical status at admission:

Antenatal ☐   Miscarriage ☐

Post-natal ☐   Termination of pregnancy ☐

2. Which of the following observations were recorded when the patient was first assessed?

	Yes	No	Not recorded
**Respiratory rate**			
**Saturations**			
**Temperature**			
**Heart rate**			
**Systolic BP**			

	Yes	No	Not recorded
**Diastolic BP**			
**Urine output**			
**Mental State**			
**Fetal heart rate**			

3. Were the observations recorded on a chart?

Yes ☐   No ☐   N/A ☐

→ If intervention phase, were they recorded on a MEOWS chart?

Yes ☐   No ☐

For peer review only

FORM 2: PATIENT CARE AUDIT FORM **PATIENT ID NUMBER:** ☐☐☐ - ☐☐☐☐

4. Has the patient been admitted for the last 24 hours

Yes ☐ No ☐

If yes, go to question 5, if No got to Question 6

5. During the last 24 hours, please state the number of times each observation has been taken and charted:

	Number of times
Respiratory rate	☐
Oxygen saturation	☐
Temperature	☐
Heart rate	☐
Systolic BP	☐

	Number of times
Diastolic BP	
Urine output	
Mental state	
Fetal heart rate	

For peer review only

REVIEW EVERY PATIENT'S NOTES WITH AN ABNORMAL OBSERVATION AND CONSIDER COMPLETING FORM 3 IF THERE IS CONCERN ABOUT MATERNAL INFECTION

Completed by: ______________________

Role: ______________________

Signature: ______________________ Date: DD / MMM / YYYY

You must have signed the Site Signature & Delegation Log

For peer review only - http://bmjopen.bmj.com/site/about/guidelines.xhtml

FORM 3: INDIVIDUAL PATIENT FORM

Screen the notes for every patient that had a suspected infection.
Only complete this form for patients that fulfil the criteria in q1-4 below.

Also complete this form for any patient you observe during the patient care audit (Form2) that fulfils the criteria below.

CONSIDER QUESTIONS 1-4 TO DETERMINE IF THIS FORM NEEDS TO BE COMPLETED

1. Was there a concern about a potential maternal infection?

   Yes ☐ No ☐ → **DO NOT COMPLETE FORM**

   Date and time suspected: d d - m m - y y y y h h : m m

2. Have observations taken?

   Yes ☐ No ☐ → **COMPLETE FORM**

3. Was there a fetal tachycardia (heart rate) greater than or equal to 160 bpm?

   Yes ☐ No ☐ Not Recorded ☐

4. Did the patient have abnormal vital signs or MEOWS chart trigger - 1 or more red flags or 2 or more yellow flags?

   Yes ☐ No ☐

IF NO TO BOTH QUESTIONS, DO NOT COMPLETE FORM

For peer review only

FORM 3: INDIVIDUAL PATIENT FORM

Patient ID number: ☐☐☐☐ ☐☐☐☐

Date of assessment medical record: d D / m m / y y y y

Time of assessment medical record: ☐☐ ☐☐

5. **Study phase:**

Baseline ☐ Intervention ☐

6. At the time of assessment for maternal infection by the health care staff, were they an inpatient or outpatient?

Inpatient ☐ Outpatient ☐

Date of admission: d d - m m - y y y y

Whilst an inpatient, was a chart used for the patient? FAST-M MEOWS chart: ☐ Other chart ☐

If no, why not? ____________________

____

7. Were the patient referred from health facility

Yes ☐ No ☐

8. What treatment(s) did the patient receive before being referred? (Tick all the boxes that have been collected)

Fluids ☐ Antibiotics ☐ Source control ☐ Monitoring ☐ Not recorded ☐

9. What was the patient's pregnancy status when infection was first suspected/diagnosed?

<12 weeks ☐ 28+ weeks ☐ Miscarriage/ Abortion within the last 6 weeks ☐

12-28 ☐ Post-natal (up to 6 weeks) ☐

10. What type of infection was suspected? (Tick one or more options)

Offensive vaginal discharge ☐

Vaginal bleeding ☐

Delay in uterine involution ☐

Abnormal pain ☐

Urinary symptoms ☐

Vomiting/diarrhoea ☐

Breast swelling/pain ☐

Cough/shortness of breath/sore throat ☐

Discharging wound/wound dehiscence ☐

Swollen /painful cannula site ☐

Fever /rigors/malaise ☐

Headache/Neck stiffness/rash ☐

Others, Please state ________________

For peer review only

FORM 3: INDIVIDUAL PATIENT FORM **PATIENT ID NUMBER** ☐☐☐-☐☐☐

11. Did the patient have any of the following observations at time infection was first suspected/diagnosed (see q1)?

	Yellow trigger		Red trigger		No abnormal Observation	Not recorded
Respiratory rate	21-24		25 or more	10 or less	11 - 20	
	☐		☐	☐	☐	☐
Oxygen Saturations			94 or less Or needing oxygen		95 or more	
			☐		☐	☐
Temperature	38 or more	35.9 or less			36.0 to 37.9	
	☐	☐			☐	☐
Heart rate	100-119	40-49	120 or more	39 or less	50 – 99	
	☐	☐	☐	☐	☐	☐
Systolic Blood Pressure	140-159	90-99	160 or more	89 or less	100 -139	
	☐	☐	☐	☐	☐	☐
Diastolic Blood Pressure	90-109		110 or more	39 or less	40 – 89	
	☐		☐	☐	☐	☐
Not passed urine	In last 12-18 hours		In over 18 hours or less than 0.5ml/kg/hr.		12 hours or less	
	☐		☐		☐	☐
Appearance	Looks unwell				Look well	
	☐				☐	☐
Mental state			Not alert		Alert	
			☐		☐	☐

Date of observation d d - m m - y y y y

Time of observation h h m m

☐ or tick if not recorded ☐ or tick if not recorded

For peer review only - http://bmjopen.bmj.com/site/about/guidelines.xhtml

FORM 3: INDIVIDUAL PATIENT FORM **PATIENT ID NUMBER** ☐☐☐-☐☐☐

12. At the time the decision tool was initiated (if intervention phase) or if not used that the infection was most serious there were:

Yellow flags ___ (enter number)

Red flags ___ (enter number)

If 2 or more yellow flags, or 1 or more red flags was the FAST-M bundle initiated (if intervention phase)?

Yes ☐ No ☐

13. If one or more red flags present? Was the FAST-M bundle initiated (within 1 hour) (if intervention phase)?

Yes ☐ No ☐
Not recorded ☐ Go to question 12

14. If 2 or more yellow flags were present, did a review take place within 3 hours?

Yes ☐ No ☐ Not recorded ☐

15. Was the patient an inpatient for 24 hours or more prior to infection being suspected/ diagnosed?

Yes ☐ No ☐

16. If yes, During the last 24 hours or more, please state the number of times each observation has been recorded prior to infection being suspected / diagnosed:

	Number of times
Respiratory rate	
Temperature	
Heart rate	
Systolic BP	

	Number of times
Diastolic BP	
Urine output	
Mental state	
Fetal heart rate	

For peer review only

FORM 3: INDIVIDUAL PATIENT FORM **PATIENT ID NUMBER** ☐☐☐-☐☐☐

17. At the first time that a yellow or red trigger occurred, was action taken within 3 hours (if two or more yellow flags) or within 1 hour (if red)?

Yes ☐ No ☐ Not Recorded ☐

18. What action was taken (answer yes or no for each option)?

	Yes	No
Medical Officer informed	☐	☐
Nurse in charge informed	☐	☐
Resident Doctor informed	☐	☐
Treatment started	☐	☐
Consultant Doctor Informed	☐	☐
Nurse	☐	☐
Decision tool started, if intervention phase?	☐	☐

Others (please state) _______________

19. Who took action (answer yes or no for each option)?

	Yes	No
	☐	☐
Medical Officer	☐	☐
Resident Doctor	☐	☐
Consultant Doctor		
Medical assistant	☐	☐

	Yes	No
Nurse	☐	☐
Nurse In-charge	☐	☐

20. If the patient was in an outpatient setting was the patient subsequently admitted?

Yes ☐ No ☐ N/A ☐

21. Was the patient commenced on an observation chart?

Yes ☐ No ☐

For peer review only

FORM 3: INDIVIDUAL PATIENT FORM **PATIENT ID NUMBER** ☐☐☐-☐☐☐

22. In the 24 hours or more after suspecting maternal infection, did the patient have their observations repeated WHILST they were awaiting the clinical review?

Yes ☐ No ☐ No, because review happened at the same time ☐

Not Done ☐

	½ Hourly	Hourly	Less Frequent than hourly
Respiratory rate			
Temperature			
Heart rate			
Systolic BP			

	½ Hourly	Hourly	Less Frequent than hourly
Diastolic BP			
Urine output			
Mental state			
Fetal heart rate			

23. Did the patient receive a clinical review within 1 or 3 hours' review of recognition of maternal infection/sepsis?

Yes ☐ No ☐

Date and time of review d d - m m - y y y y h h : m m

For peer review only

FORM 3: INDIVIDUAL PATIENT FORM

PATIENT ID NUMBER ☐☐☐-☐☐☐

a. Did the patient receive a clinical review within 3 hours? Yes ☐ No ☐
b. Did the patient receive a clinical review within 1 hour? Yes ☐ No ☐

24. Following the clinical review was the specified frequency of observations given?
Yes ☐ No ☐

25. Did the patient undergo observations at this specified frequency?
Yes ☐ No ☐

26. Was the patient treated for maternal infection/sepsis?
Yes ☐ No ☐

27. If intervention phase was the FAST-M decision tool was used for the patient?
Yes ☐ No ☐

MANAGEMENT

28. Were IV fluids given?

Yes ☐ No ☐ Why not? ____________________

Date of started: d d - m m - y y y y

Time of started h h m m

☐ or tick if not recorded

☐ or tick if not recorded

29. Were antibiotics administered within one hour?

Yes ☐ No ☐ Why not? ____________________

Date of administered: d d - m m - y y y y

Time of administered: h h m m

☐ or tick if not recorded

☐ or tick if not recorded

For peer review only

FORM 3: INDIVIDUAL PATIENT FORM **PATIENT ID NUMBER** ☐☐☐-☐☐☐

SOURCE CONTROL/ IDENTIFICATION

30. Was an attempt made to identify the source of infection?

Yes ☐ No ☐ Why not? ________________________________

Date: d d - m m - y y y y Time: h h m m

☐ or tick if not recorded ☐ or tick if not recorded

What attempts were made to identify the source?

	Yes	No
Clinical history	☐	☐
Clinical examination	☐	☐
Blood test (if available)	☐	☐
Blood cultures	☐	☐
HIV test	☐	☐
Malaria test	☐	☐
Urine sample	☐	☐
Swabs (wound, vagina, throat)	☐	☐
Imaging (abdominal, chest)	☐	☐
Lumbar puncture	☐	☐
Sputum sample	☐	☐
Others (please state) ________________		

31. Was the source identified?

Yes ☐ No ☐

Date: d d - m m - y y y y Time: h h m m

☐ or tick if not recorded ☐ or tick if not recorded

For peer review only

FORM 3: INDIVIDUAL PATIENT FORM

PATIENT ID NUMBER ☐☐☐-☐☐☐

What was the source of infection?

	Yes	No
Breast	☐	☐
Chorioamnionitis	☐	☐
Endometritis	☐	☐
Infected Cannula/ line	☐	☐
Malaria	☐	☐
Meningitis/ cerebral infection	☐	☐
Other wound/ skin infection	☐	☐
Peritonitis	☐	☐
Respiratory infection	☐	☐
Urinary Tract Infection	☐	☐

Other (please state)________________________

32. Was the source treated?

Yes ☐ No ☐ Why not? ______________________________

Date: d d - m m - y y y y Time: h h m m

☐ or tick if not recorded ☐ or tick if not recorded

How was it treated?

	Yes	No
Delivery of the baby	☐	☐
Removal of retained products of conception	☐	☐
Hysterectomy	☐	☐
Removal of infected cannula/line	☐	☐
Targeted antibiotics	☐	☐
Malaria treatment	☐	☐
Antivirals	☐	☐
Incision and drainage	☐	☐
Percutaneous drainage	☐	☐
Debridement of wound	☐	☐
Laparotomy and washout	☐	☐

Other (Please state) ____________________

For peer review only

FORM 3: INDIVIDUAL PATIENT FORM

PATIENT ID NUMBER ☐☐☐-☐☐☐

TRANSPORT

33. Was there a need to transport the patient to a higher level of care considered?

Yes ☐ No ☐ Why not? ____________________ Not recorded ☐

Date: d d - m m - y y y y Time: h h m m

☐ or tick if not recorded ☐ or tick if not recorded

34. Was the patient transferred?

Yes ☐ No ☐ Why not? ____________________ Not recorded ☐

Date requested: d d - m m - y y y y Time requested: h h m m

☐ or tick if not recorded ☐ or tick if not recorded

Date patient left the facility: d d - m m - y y y y Time: h h m m

☐ or tick if not recorded ☐ or tick if not recorded

35. Where was the patient transferred to? ____________________

36. Was a transfer letter sent with the patient?

Yes ☐ No ☐ Why not? ____________________

For peer review only

FORM 3: INDIVIDUAL PATIENT FORM **PATIENT ID NUMBER** ☐☐☐-☐☐☐

MONITORING

37. Did the patient have ongoing monitoring commenced after the recognition of maternal suspected/sepsis?

Yes ☐ No ☐ → N/A (24hrs hadn't elapsed) ☐

	1/2 hourly or Less	Hourly or Less	Daily or More	Less than Daily	not done
Respiratory rate	☐	☐	☐	☐	☐
Temperature	☐	☐	☐	☐	☐
Heart rate	☐	☐	☐	☐	☐
Systolic BP	☐	☐	☐	☐	☐

	½ hourly or Less	Hourly or Less	Daily or More	Less than Daily	not done
Diastolic BP	☐	☐	☐	☐	☐
Urine output	☐	☐	☐	☐	☐
Neuro Response	☐	☐	☐	☐	☐
Fetal heartrate	☐	☐	☐	☐	☐

For peer review only

FORM 3: INDIVIDUAL PATIENT FORM

PATIENT ID NUMBER ☐☐☐-☐☐☐

PATIENT OUTCOMES

38. Did the patient have any of the following complications recorded during their treatment?

	Yes	No
Pulmonary oedema	☐	☐
Blood transfusion	☐	☐
Return to theatre	☐	☐
Allergic reaction	☐	☐

	Yes	No
Change in mental state	☐	☐
Reduced urine output (no output for >18 hours or <0.5ml/kg/hr)	☐	☐
Other	☐	☐
Please state	☐	☐

39. Was the patient admitted to high dependency/ intensive care during their treatment?

Yes ☐ No ☐

No. of days: ☐ Days

40. Did the patient have any of the following near miss events recorded during their stay?

Cardiovascular dysfunction		
**Event**	**Yes**	**No**
Shock	☐	☐
Cardiac arrest	☐	☐
Severe hypo perfusion (lactate >5mmol/L)	☐	☐
Severe acidosis (pH<7.1)	☐	☐
Use of continuous vasoactive drugs	☐	☐
Cardio-pulmonary resuscitation	☐	☐
**Respiratory dysfunction**		
Acute cyanosis	☐	☐
Gasping	☐	☐
Severe tachypnea (respiratory rate >40)	☐	☐
Severe bradypnea (respiratory rate <6)	☐	☐
Severe hypoxaemia (O2 sats <90% for >60 mins or PAO2/FiO2 <200)	☐	☐
Intubation and ventilation not related to anaesthesia	☐	☐

For peer review only

FORM 3: INDIVIDUAL PATIENT FORM **PATIENT ID NUMBER** ☐☐☐-☐☐☐

PATIENT OUTCOMES

Renal dysfunction		
Oliguria non-responsive to fluids or diuretics	☐	☐
Creatinine >300 µmol/ml or >3.5 mg/dL	☐	☐
Dialysis for acute renal failure	☐	☐
**Coagulation dysfunction**		
Failure to form clots	☐	☐
Severe acute thrombocytopenia (50,000 platelets/ml)	☐	☐
Massive transfusion of blood or red cells (≥5 units)	☐	☐
**Hepatic dysfunction**		
Jaundice in the presence of pre-eclampsia	☐	☐
Severe acute hyperbilirubinemia (bilirubin>100 µmol/L or >6.0 mg/dL)	☐	☐
**Neurological dysfunction**		
Prolonged unconsciousness or coma (lasting >12 hours)	☐	☐
Stroke	☐	☐
Uncontrollable fit / status epilepticus	☐	☐
Global paralysis	☐	☐
**Uterine dysfunction**		
Hysterectomy due to uterine infection or haemorrhage	☐	☐

For peer review only

FORM 3: INDIVIDUAL PATIENT FORM

PATIENT ID NUMBER ☐☐☐-☐☐☐

PATIENT OUTCOMES

41. Date of final discharge from hospital/health centre: d d - m m - y y y y

42. Total number of days spent in hospital: ☐ days

43. What was the outcome of the patient at discharge?

Alive and well, no follow-up required ☐

Alive and well, follow-up arranged ☐

Death ☐

Date of death: d d - m m - y y y y

Time of Death: h h : m m

Cause of death: ____________________

PREGNANCY OUTCOMES (if event occurred during same admission)

Tick if not applicable ☐

44. What was the pregnancy outcome?

Miscarriage ☐

Ectopic pregnancy ☐

Induced abortion ☐

Live birth ☐

Still birth (> 28 weeks) ☐

Pregnancy terminated before admission ☐

Date occurred or diagnosed: d d - m m - y y y y

For peer review only

FORM 3: INDIVIDUAL PATIENT FORM **PATIENT ID NUMBER** ☐☐☐-☐☐☐

45. Was monitoring of the new-born commenced?

Yes ☐ No ☐ N/A ☐

Date: d d - m m - y y y y Time: h h m m

☐ or tick if not recorded ☐ or tick if not recorded

46. Was fetal monitoring done if the patient was antenatal?

Yes ☐ No ☐ N/A ☐

Date: d d - m m - y y y y Time: h h m m

☐ or tick if not recorded ☐ or tick if not recorded

For peer review only

FORM 3: INDIVIDUAL PATIENT FORM **PATIENT ID NUMBER** ☐☐☐-☐☐☐

NEONATAL OUTCOMES AT BIRTH

Tick if not applicable ☐

47. Gestation: ☐ Months OR ☐ weeks ☐ days

48. Weight: ☐ g Not recorded ☐

49. Did the baby have any of the following complications?

	Yes	No	Not recorded
Resuscitation	☐	☐	☐
Agpars below 7 at 5 mins after birth	☐	☐	☐
Admitted to neonatal unit/special care	☐	☐	☐
Other (Please state)	☐	☐	________________

50. Was the baby thought to have sepsis?

Yes ☐ No ☐

Did the baby receive antibiotic prophylaxis anyway?

Yes ☐ No ☐

Were they treated for sepsis?

Yes ☐ No ☐ Not recorded

51. What was the outcome of the baby at discharge?

Alive and well, no follow-up required ☐

Alive and well, follow-up required ☐

Death ☐

Date of Death d d - m m - y y y y

Time of Death: h h m m

Cause of Death ________________

For peer review only

FORM 3: INDIVIDUAL PATIENT FORM **PATIENT ID NUMBER** [ ][ ][ ] - [ ][ ][ ]

Completed by: ______________________________

Role: ______________________________

Signature: ______________________________ Date: DD / MM / YYYY

You must have signed the Site Signature & Delegation Log

For peer review only

Supplemental File 5

APPENDIX FIGURE 14 – Participant Study ID Number: ___/_____– PATIENT CONSENT FORM (ENGLISH) for patients agreeing to access of their notes

Title of study: Extension of the FAST- M maternal sepsis bundle in Pakistan, a feasibility study

What is the purpose of the overall study?

We are developing a way of improving the care of women with pregnancy or birth related infections around the world.

We are introducing three things: 1. a chart to help the nurses record your vital signs and detect infection before it becomes worse 2. A tool to help the medical staff treat infection 3. A training day to teach the medical staff about pregnancy or birth related infections

We hope that this will make caring for patients with pregnancy or birth related infections easier.

Why have I been invited to participate?

We are asking all patients pregnant or recently pregnant patients who are receiving treatment in hospital or who have an infection to participate. Being invited to take part does not necessarily mean you have an infection.

What will I have to do if I take part?

Nothing. If you agree to take part, you will receive the normal care. All we ask is that our research team from Aga Khan University Hospital can look at your medical notes to see what happened to you whilst you were in hospital. The information from your medical notes will help us decide if our study is working.

Do I have to take part?

No you do not have to take part it is your decision. If you don't want to take part that is fine, it will not affect how you are cared for when you are in hospital.

Will my taking part in the study be kept confidential?

Yes. All the information from your notes will be stored securely on a password protected computer system, which will be backed-up in a separate location to keep this information safe.

What are the possible benefits of taking part?

By giving us permission to look at your notes we hope that we will be able to find out if the study is working. If the study works, then this will hopefully help to improve the care of mothers with infections.

What will happen if I don't want to carry on with the study?

You may withdraw from the study without giving a reason for up to one month after. If you wish to withdraw we will delete all the information we had collected about you.

What if there is a problem?

For peer review only

If you have a concern about any aspect of this study, you can speak to the researchers, who will do their best to answer your questions. Their contact details are on the next page.

Who is organizing the research?

This study is being carried out by the Aga Khan University Hospital(AKUH), Pakistan in affiliation with University of Birmingham, UK and University of Liverpool, UK.

AKUH team will be facilitating the day to day running of the study, including looking at your medical notes and collecting the information.

Who has reviewed the study?

This study has been reviewed by the National Bioethics Committee Pakistan and AKUH Research Ethics Committee.

Contact details:

Lead Facilitator: Dr Sheikh Irfan Ahmed, Senior Instructor, AKUH National stadium road, Karachi

Email: sheikh.irfan@aku.edu Telephone number: +92-021-34864650

Registered study ID number:

PLEASE INITIAL THE BOXES IF YOU AGREE WITH EACH SECTION:

1. I understand that my participation is voluntary and that I am free to withdraw up to one month after my participation without giving any reason. ☐
2. I agree for my notes to be accessed by the AKUH research team. I understand that all data collected about me will be anonymised and will not contain any identifiable information. ☐
3. I understand that the researchers might publish the results of this study. I give permission for anonymized information from my notes to be used for this purpose. ☐
4. I know how to contact the research team if I need to. ☐
5. I am happy for information about me might also be retained to include it anonymously in future studies
6. I agree to participate in this study. ☐

SIGNATURES:

____________________________ ____________ ________________________ Patient name and surname

Date Signature / Thumbprint

For peer review only

___________________________ ___________ _______________________ Name and Surname of person

Date Signature taking consent

Participant Study ID Number: _____/_____

For peer review only

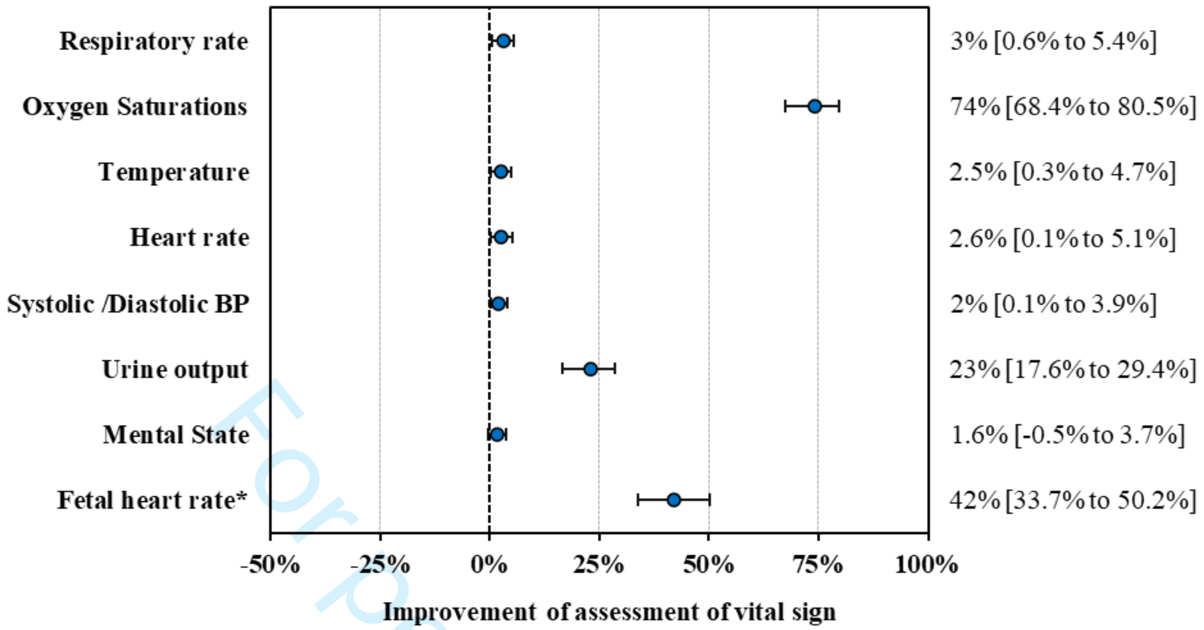

Supplement Figure 6: Patient's assessment of vital signs on admission (n=439)

For peer review only

Supplemental File 7

Table: Characteristics of suspected maternal sepsis patients (n=138)

Variables	Baseline n=60	Intervention n=78
**Gestational at presentation**		
<12 weeks	2(3.3%)	0(0%)
12-28 weeks	2(3.3%)	1(1.3%)
>28 weeks	31(51.7%)	38(48.7%)
Post-natal (up to 6 weeks)	25(41.7%)	39(50%)
**Abnormal Trigger**		
Respiratory Rate (>25 or <10 per minute)	41(68.3%)	41(52.6%)
Temp (>38 or <35.9 °C)	25(41.7%)	35(44.9%)
Heart Rate (>120 or <39 beats per minute)	32(53.3%)	38(48.7%)
Systolic Blood Pressure (>160 or <89 mmhg)	43(71.7%)	43(55.1%)
Diastolic Blood Pressure (>110 or <39 mmhg)	35(58.3%)	38(48.7%)
Urine Output (In over 18 hours or less than 0.5ml/kg/hr)	4(6.7%)	4(5.1%)
Mental State (Not alert)	9(15%)	28(35.9%)
Appearance (Looks unwell)	7(11.7%)	49(62.8%)
**Source of maternal infection/sepsis***		
Breast	1(1.7%)	1(1.3%)
Chorioamnionitis	13(21.7%)	7(9%)
Endometritis	4(6.7%)	2(2.6%)
Infected Cannula/ line	1(1.7%)	0(0%)
Malaria	0(0%)	5(6.4%)
Meningitis/ cerebral infection	1(1.7%)	0(0%)
Other wound/ skin infection	3(5%)	5(6.4%)
Peritonitis	2(3.3%)	7(9%)
Respiratory infection	29(48.3%)	14(17.9%)
Urinary Tract Infection	9(15%)	6(7.7%)
Others	13(21.7%)	31(39.7%)
Unknown	7(11.7%)	14(17.9%)

*Thirty patients had multiple infection

For peer review only

Supplemental file 8

Table: Laboratory investigations of suspected maternal sepsis patients

Laboratory Investigations	Baseline phase (n=60)	Intervention phase (n=78)
Blood test (if available)	55 (92%)	78 (100%)
Blood cultures	27 (45%)	67 (86%)
HIV test	52 (87%)	72 (92%)
Malaria test	1 (2%)	21 (27%)
Urine sample	10 (17%)	63 (81%)
Swabs (wound, vagina, throat)	9 (15%)	2 (3%)
Imaging (abdominal, chest)	8 (13%)	38 (49%)
Lumbar puncture	0 (0%)	2 (3%)
Sputum sample	2 (3%)	2 (3%)

For peer review only

Supplemental File 9

Table: Assessment of Maternal statistics (from facility audit CRF1)

Total Admissions (N=24,000)	January to June 2021 (n=11205)	July to December 2021 (n=12795)	P-value
Maternal Sepsis	210 (1.8%)	160 (1.2%)	.0005
Maternal Mortality	159 (1.4%)	120 (0.9%)	.001
Post-Partum Hemorrhage	110 (0.9%)	175 (1.36%)	.006
Antepartum Hemorrhage	194 (1.73%)	110 (0.85%)	.0005
Severe Pre-Eclampsia	133 (1.18%)	88 (0.68%)	.0005
Uterine Rupture	36 (0.32%)	28 (0.21%)	.125

For peer review only

STROBE Statement—checklist of items that should be included in reports of observational studies

	Item No	Recommendation	Page No
**Title and abstract**	1	(*a*) Indicate the study's design with a commonly used term in the title or the abstract	1,3
		(*b*) Provide in the abstract an informative and balanced summary of what was done and what was found	3
**Introduction**			
Background/rationale	2	Explain the scientific background and rationale for the investigation being reported	5-6
Objectives	3	State specific objectives, including any prespecified hypotheses	6
**Methods**			
Study design	4	Present key elements of study design early in the paper	7
Setting	5	Describe the setting, locations, and relevant dates, including periods of recruitment, exposure, follow-up, and data collection	7-12
Participants	6	(*a*) *Cohort study*—Give the eligibility criteria, and the sources and methods of selection of participants. Describe methods of follow-up<br>*Case-control study*—Give the eligibility criteria, and the sources and methods of case ascertainment and control selection. Give the rationale for the choice of cases and controls<br>*Cross-sectional study*—Give the eligibility criteria, and the sources and methods of selection of participants	7-8
		(*b*) *Cohort study*—For matched studies, give matching criteria and number of exposed and unexposed<br>*Case-control study*—For matched studies, give matching criteria and the number of controls per case	
Variables	7	Clearly define all outcomes, exposures, predictors, potential confounders, and effect modifiers. Give diagnostic criteria, if applicable	11
Data sources/ measurement	8*	For each variable of interest, give sources of data and details of methods of assessment (measurement). Describe comparability of assessment methods if there is more than one group	12
Bias	9	Describe any efforts to address potential sources of bias	11-12
Study size	10	Explain how the study size was arrived at	12-13
Quantitative variables	11	Explain how quantitative variables were handled in the analyses. If applicable, describe which groupings were chosen and why	12
Statistical methods	12	(*a*) Describe all statistical methods, including those used to control for confounding	12
		(*b*) Describe any methods used to examine subgroups and interactions	
		(*c*) Explain how missing data were addressed	
		(*d*) *Cohort study*—If applicable, explain how loss to follow-up was addressed<br>*Case-control study*—If applicable, explain how matching of cases and controls was addressed<br>*Cross-sectional study*—If applicable, describe analytical methods taking account of sampling strategy	

For peer review only - http://bmjopen.bmj.com/site/about/guidelines.xhtml

(*e*) Describe any sensitivity analyses

Continued on next page

For peer review only

For peer review only - http://bmjopen.bmj.com/site/about/guidelines.xhtml

**Results**			
Participants	13*	(a) Report numbers of individuals at each stage of study—eg numbers potentially eligible, examined for eligibility, confirmed eligible, included in the study, completing follow-up, and analysed	8,12-13
		(b) Give reasons for non-participation at each stage	12
		(c) Consider use of a flow diagram	8
Descriptive data	14*	(a) Give characteristics of study participants (eg demographic, clinical, social) and information on exposures and potential confounders	13
		(b) Indicate number of participants with missing data for each variable of interest	12-13
		(c) *Cohort study*—Summarise follow-up time (eg, average and total amount)	NA
Outcome data	15*	*Cohort study*—Report numbers of outcome events or summary measures over time	*NA*
		Case-control study—Report numbers in each exposure category, or summary measures of exposure	*NA*
		Cross-sectional study—Report numbers of outcome events or summary measures	12-15
Main results	16	(*a*) Give unadjusted estimates and, if applicable, confounder-adjusted estimates and their precision (eg, 95% confidence interval). Make clear which confounders were adjusted for and why they were included	NA
		(*b*) Report category boundaries when continuous variables were categorized	NA
		(*c*) If relevant, consider translating estimates of relative risk into absolute risk for a meaningful time period	NA
Other analyses	17	Report other analyses done—eg analyses of subgroups and interactions, and sensitivity analyses	12-15
**Discussion**			
Key results	18	Summarise key results with reference to study objectives	15
Limitations	19	Discuss limitations of the study, taking into account sources of potential bias or imprecision. Discuss both direction and magnitude of any potential bias	19-20
Interpretation	20	Give a cautious overall interpretation of results considering objectives, limitations, multiplicity of analyses, results from similar studies, and other relevant evidence	15-20
Generalisability	21	Discuss the generalisability (external validity) of the study results	20
**Other information**			
Funding	22	Give the source of funding and the role of the funders for the present study and, if applicable, for the original study on which the present article is based	21

*Give information separately for cases and controls in case-control studies and, if applicable, for exposed and unexposed groups in cohort and cross-sectional studies.

Note: An Explanation and Elaboration article discusses each checklist item and gives methodological background and published examples of transparent reporting. The STROBE checklist is best used in conjunction with this article (freely available on the Web sites of PLoS Medicine at http://www.plosmedicine.org/, Annals of Internal Medicine at http://www.annals.org/, and Epidemiology at http://www.epidem.com/). Information on the STROBE Initiative is available at www.strobe-statement.org.

For peer review only - http://bmjopen.bmj.com/site/about/guidelines.xhtml

BMJ Open

Early recognition and management of maternal sepsis in Pakistan: A feasibility study of the implementation of FAST-M Intervention

Journal:	*BMJ Open*
Manuscript ID	bmjopen-2022-069135.R2
Article Type:	Original research
Date Submitted by the Author:	28-Jun-2023
Complete List of Authors:	Ahmed, Sheikh; The Aga Khan University, Obs and Gynae<br>Rind , Ghulam Kubra; The Aga Khan University Hospital<br>Sikandar , Raheel ; Liaquat University of Medical and Health Sciences, OBGYN<br>Raza , Amir ; The Aga Khan University Hospital Main Campus Karachi<br>Khowaja, Bakhtawar; The Aga Khan University Hospital Main Campus Karachi<br>Parveen, Fahmida; Liaquat University of Medical and Health Sciences<br>Khan , Sehrish ; Liaquat University of Medical and Health Sciences<br>Memon, Nazia; Liaquat University of Medical and Health Sciences<br>Jahangir, Arshia ; The Aga Khan University<br>Mirza, Daayl ; Ziauddin University<br>Cheshire , James ; University of Birmingham<br>Dunlop, Catherine; University of Birmingham<br>Shakoor, Sadia; The Aga Khan University Hospital Main Campus Karachi<br>Barolia, Rubina; The Aga Khan University Hospital Main Campus Karachi<br>Sheikh, Lumaan; The Aga Khan University Hospital Main Campus Karachi<br>Saleem, Sarah; The Aga Khan University<br>Coomarasamy, Arri; University of Birmingham<br>Lissauer, David ; Malawi-Liverpool-Wellcome Trust Clinical Research Programme, ; University of Liverpool,
**Primary Subject Heading**:	Obstetrics and gynaecology
Secondary Subject Heading:	Infectious diseases, Obstetrics and gynaecology, Evidence based practice
Keywords:	OBSTETRICS, INFECTIOUS DISEASES, Maternal medicine < OBSTETRICS

SCHOLARONE™
Manuscripts

I, the Submitting Author has the right to grant and does grant on behalf of all authors of the Work (as defined in the below author licence), an exclusive licence and/or a non-exclusive licence for contributions from authors who are: i) UK Crown employees; ii) where BMJ has agreed a CC-BY licence shall apply, and/or iii) in accordance with the terms applicable for US Federal Government officers or employees acting as part of their official duties; on a worldwide, perpetual, irrevocable, royalty-free basis to BMJ Publishing Group Ltd ("BMJ") its licensees and where the relevant Journal is co-owned by BMJ to the co-owners of the Journal, to publish the Work in this journal and any other BMJ products and to exploit all rights, as set out in our licence.

The Submitting Author accepts and understands that any supply made under these terms is made by BMJ to the Submitting Author unless you are acting as an employee on behalf of your employer or a postgraduate student of an affiliated institution which is paying any applicable article publishing charge ("APC") for Open Access articles. Where the Submitting Author wishes to make the Work available on an Open Access basis (and intends to pay the relevant APC), the terms of reuse of such Open Access shall be governed by a Creative Commons licence – details of these licences and which Creative Commons licence will apply to this Work are set out in our licence referred to above.

Other than as permitted in any relevant BMJ Author's Self Archiving Policies, I confirm this Work has not been accepted for publication elsewhere, is not being considered for publication elsewhere and does not duplicate material already published. I confirm all authors consent to publication of this Work and authorise the granting of this licence.

For peer review only

Paper Title:

Early recognition and management of maternal sepsis in Pakistan: A feasibility study of the implementation of FAST-M Intervention

Corresponding Author*

Sheikh Irfan Ahmed

Aga Khan University Hospital, National Stadium Road, Karachi city, Pakistan. P.O. Box 3500 Postal code: 74800

Email: sheikh.irfan@aku.edu

Author Names:

Sheikh Irfan Ahmed[1]

Ghulam Kubra Rind[1]

Raheel Sikandar[2]

Amir Raza[1]

Bakhtawar M.Hanif Khowaja[1]

Fahmida Parveen [2]

Sehrish Khan[2]

Nazia Memon [2]

Arshia Jahangir[3]

Daayl Naim Mirza[4]

James Cheshire[5]

Catherine Dunlop [5]

Sadia Shakoor[1]

Rubina Barolia[1]

Lumaan Sheikh[1]

Sarah Saleem[1]

Arri Coomarasamy[5]

David Lissauer[6, 7]

Affiliations & full institutional mailing addresses of all authors

[1] Aga Khan University Hospital, National Stadium Road, Karachi city, Pakistan. P.O. Box 3500 Postal code: 74800

[2] LUMHS Hospital Liaquat University of Health and Medical Sciences, Hyderabad city, Pakistan. Postal Code: 76090

[3] Aga Khan University, National Stadium Road, Karachi city, Pakistan. P.O. Box 3500 Postal code: 74800

[4] Ziauddin University4/B Shahrah-e-Ghalib Rd, Block 6 Clifton, Karachi, Karachi City, Sindh 75000

For peer review only

[5] Institute of Metabolism and Systems Research, University of Birmingham, Edgbaston, Birmingham, UK, B15 2TT.

[6] Institute of Life Course and Medical Sciences, University of Liverpool, Liverpool, William Henry Duncan Building, Liverpool, UK, L7 8TX.

[7] Malawi-Liverpool-Wellcome Clinical Research Programme, Chichiri, Blantyre 3, Malawi

Email addresses

sheikh.irfan@aku.edu (SI)

ghulam.kubra@aku.edu (GK)

raheel.sikandar@lumhs.edu.pk (RS)

amir.raza@aku.edu (AR)

bakhtawar.hanif@aku.edu (BK)

dr_memon16@yahoo.com (FP)

drkhanhyd@gmail.com (SK)

nazia.memon23@yahoo.com (NM)

arshia.jahangir@scholar.aku.edu (AJ)

daayl.13094@zu.edu.pk (DM)

sadia.shakoor@aku.edu (SSH)

james.cheshire@nhs.net (JC)

catherinedunlop@nhs.net (CD)

rubina.barolia@aku.edu (RB)

lumaan.sheikh@aku.edu (LS)

sarah.saleem@aku.edu (SS)

A.Coomarasamy@bham.ac.uk (AC)

David.Lissauer@liverpool.ac.uk (DL)

Word Count: 4693

For peer review only

ABSTRACT

Objective: Maternal sepsis is the 3rd leading cause of maternal mortality globally. World Health Organization and collaborators developed a care bundle called FAST-M (**F**luids, **A**ntibiotics, **S**ource identification and treatment, **T**ransfer, and **M**onitoring) for early identification and management of maternal sepsis in low-resource settings. This study aimed to determine feasibility of FAST-M intervention in a low-resource setting in Pakistan. The FAST-M intervention consists of maternal sepsis screening tools, treatment bundle and implementation program.

Design and setting: A feasibility study with before and after design was conducted in women with suspected maternal sepsis admitted at the LUMHS hospital Hyderabad. The study outcomes were compared between baseline and intervention phase. In the baseline phase (2 months), the existing sepsis care practices were recorded, followed by a training program for healthcare providers on the application of FAST-M tools. These tools were implemented in the intervention phase (4 months) to assess any change in clinical practices compared to the baseline phase.

Results: During the FAST-M implementation, 439 women were included in the study. 242/439 were suspected maternal infection cases, and 138/242 were women with suspected maternal sepsis. The FAST-M bundle was implemented in women with suspected maternal sepsis. Following the FAST-M intervention, significant changes were observed. Improvements were seen in the monitoring of oxygen saturation measurements [25.5% vs 100%; difference: 74%; 95% CI:68.4%-80.5% p<0.01], fetal heart rate assessment [58% vs100%; difference:42.0%; 95%CI: 33.7% to 50.3%; p=<0.01], and measurement of urine output [76.5% vs 100%; difference 23.5%; 95%CI: 17.6% to 29.4%; p<0.01]). Women with suspected maternal sepsis received all components of the treatment bundle within one hour of sepsis recognition [0% vs70.5%; difference 70.5%; 95%CI: 60.4% to 80.6%; p<0.01].

Conclusion: Implementation of the FAST-M intervention was considered feasible and enhanced early identification and management of maternal sepsis at the study site.

Trial registration number: ISRCTN17105658

For peer review only

Strengths and limitations:

- The study design provided the possibility to analyse operational, and practical factors for introducing the FAST-M intervention in the local setting.
- The tools used in the study were adapted in the context of Pakistan before its implementation.
- The study design does not account for the temporal effects, hence is exposed to possible selection and reporting bias.
- This study was conducted at only one tertiary level hospital.
- The intervention period was of only four months.

For peer review only

INTRODCUTION

Maternal sepsis is ‘a life-threatening condition defined as organ dysfunction resulting from infection during pregnancy, childbirth, post-abortion, or the post-partum period’.[1] Worldwide, it’s the 3rd most common cause of maternal mortality and represents around 11% of maternal deaths.[2] Pregnant patients and those that have recently delivered are more susceptible to maternal infections that can rapidly progress towards sepsis. [3,4]

Modelling studies, based on currently available data, observed that globally around 17000 maternal deaths per year may be attributed to maternal sepsis and other maternal infections (MSMI).[5] Approximately 5·7 million women suffered complications of maternal sepsis globally in 2017.[6] Annually, 99% of the 302,000 maternal deaths occurred in LMICs,[7] with the highest rates reported in Africa and Asia.[7,8] The overall maternal mortality ratio (MMR) in Pakistan is 186 per 100,000 live births in 2020,[9] and sepsis ranked as the 3rd leading cause of maternal deaths (16.3%)[10,11] following haemorrhage and eclampsia.[2]

In 2015, the Global maternal and neonatal sepsis initiative was introduced by the WHO and other partners to design strategies that could help in improving the recognition and management of maternal and neonatal sepsis in low-resource settings.[12] This initiative aimed to develop and test the effective strategies to prevent, detect, and manage the maternal and neonatal deaths caused by sepsis.[12] This included the key recommendation to develop a sepsis care bundle specific to the maternal population.

The use of care bundles in the management of sepsis has been found to reduce mortality and improve patient outcomes in high-income countries.[13] Most notably, the surviving sepsis campaign (SSC) bundles,[14,15] as well as the UK Sepsis Trusts' Sepsis 6 bundle[16] have been widely used.[15,16] Despite this, there was no sepsis care bundle in particular for the maternal population which can be implemented efficiently in low-resource settings.[17-19]

For peer review only - http://bmjopen.bmj.com/site/about/guidelines.xhtml

To address this, a care bundle for treatment of maternal sepsis in low resource settings was developed after reaching the consensus utilising a modified Delphi approach by an international health expert panel.[20] The selected components were Fluids, Antibiotics, Source identification and control, Transfer to an appropriate level of care, and ongoing Monitoring of mother and neonate. The bundle was named "FAST-M" as a memorable acronym for supporting implementation and communication.[20] The FAST-M bundle was first implemented in 15 government healthcare facilities in Malawi, and it was not only found to be feasible to implement but also resulted in improved clinical care.[21]

Despite existence of national sepsis guidelines for Pakistan (SGP), their uptake remains low especially for obstetric patients.[22] Additionally, there is uncertainty that the existing evidence-based approach to prevent, recognise, and treat maternal sepsis is effective and implementable in a low-resource setting. It was, therefore, planned to determine the feasibility of the FAST-M intervention in the local setting before recommending its implementation at scale. The study protocol and procedures have been described in detail and published elsewhere.[23]

To optimise the use of the FAST-M intervention in Pakistan, a qualitative study was conducted to understand the existing sepsis management practices/behaviours, and to identify potential gaps and resource availability in the local setting.[24] Based on the qualitative findings described in a separate paper and availability of resources in the facility, the FAST-M bundle care tools which were used in Malawi[21] were modified to plan a practical approach for the implementation of the intervention in our local context. [24] This study aimed to determine whether the FAST-M intervention is feasible to introduce for the early detection and management of maternal sepsis in a low-resource setting in Pakistan. We hypothesise that the FAST-M intervention in this adapted form could be successfully implemented and would then improve maternal sepsis care, including its early recognition and management.

For peer review only

METHODS

A prospective feasibility study with a before and after design was conducted at the Liaquat University of Medical and Health Sciences, (LUMHS) Hyderabad, Pakistan from the period of June 2021 to December 2021. Women admitted to obstetrics and gynecology units of the facility, who were pregnant, or within 6 weeks of miscarriage, ectopic pregnancy, termination of pregnancy, or delivery were considered eligible for this study. These eligible patients were monitored on MEOWS chart during their inpatient admission. Those who had abnormal maternal observations, or healthcare practitioner's concern regarding potential maternal infection, or pregnant women with a fetal tachycardia greater than or equal to 160 beats per minute were included in the study. There were no exclusion criteria for the study.

In the baseline phase, data were collected from facility record using CRF1 (facility audit form) to assess available resources for sepsis management and overall maternal outcomes in last six months. Data of patients who were meeting eligibility criteria (Fig.1.) were recorded on CRF 2 and CRF 3 to assess existing practices for management of maternal sepsis. Following this, the intervention phase was carried out. At first, HCPs were trained on the use of FAST-M bundle care tools (MEOWS chart, decision tool, and treatment bundle) to early identify and manage sepsis patients. The HCPs training was an ongoing process throughout the implementation of the intervention. The FAST-M bundle care tools were then implemented in the intervention phase in the facility and data of patients meeting the eligibility criteria were recorded using CRF 2 and CRF 3. The flow of study participants has been demonstrated in **(Figure 1).**

Comparisons were made between baseline and intervention phase to assess any change in time and frequency of vital signs monitoring (on MEOWS chart) of patients, and application of all components of FAST-M treatment bundle (supplemental file 1) within an hour of sepsis recognition. Time 0 or start time was considered when the patient had developed one or more red trigger/s on the MEOWS chart during an inpatient stay. Same applied for the patient transferred from other facilities already on treatment. Maternal outcomes (sepsis rate and related mortality) were also compared between baseline and intervention phase.

For peer review only

Intervention:

The planned intervention consisted of 3 components:

1. Maternal sepsis screening tools
2. FAST-M treatment bundle
3. FAST-M implementation Programme

Component 1- Maternal sepsis screening tools

Maternal sepsis screening tools included the Modified Early Obstetric Warning Scores chart (MEOWS) and the FAST-M decision tool **(Supplemental File 1**). MEOWS is the tool adapted by Malawi study based on an early warning score approach and simplified from the approach adopted in UK hospitals.[25] The tool assisted in charting an individual's physiological parameters over time with guiding thresholds that signal clinical action if they become abnormal.[26]

The MEOWS chart used in FAST-M implementation in the districts of Malawi [21] was adapted in the context of Pakistan for this study. During the adaptation phase, the health care practitioners recommended the inclusion of oxygen saturation into the MEOWs chart to determine patient's clinical condition considering the outbreak of COVID-19 infection and the availability of pulse oximeters at the study site. Therefore, the oxygen saturation component was incorporated in the modified MEOWS chart based on the scope of existing practices and the significance of this indicator in the identification of patient's clinical condition.

In the intervention period, eligible patients who had abnormal observations (indicated by a single red or two yellow flags) on MEOWS chart (Supplemental file 1) screened for potential sepsis using the FAST-M decision tool. Those who developed a red trigger on the MEOWS chart received an immediate clinical review. FAST-M treatment bundle was initiated within an hour on clinician's judgment on suspicion of sepsis and continued until advised to end the treatment.

For peer review only - http://bmjopen.bmj.com/site/about/guidelines.xhtml

Those patients who triggered two yellow flags on the MEOWS chart and were thought to have possible infection were reviewed within three hours. All suspected cases (those without red or two yellow triggers, were still considered for having a risk of sepsis and remained under observation for possible development of red or yellow triggers), these patients were monitored on the MEOWS chart hourly throughout the day and were managed as per local guidelines.

Component 2-FAST-M Treatment Bundle

The FAST-M treatment bundle consists of Fluids, Antibiotics, Source identification and Control, Assessment of the need to Transport / Transfer to a high level of care, and Ongoing Monitoring (of the mother and neonate). The first and second-line antibiotic therapy guidelines were modified as per the local context during the adaptation phase. [24] **(Supplemental File 1)**. Patients with suspected sepsis were commenced immediately on the FAST-M treatment bundle within an hour of identification of sepsis. Each patient's treatment was recorded on the treatment bundle, along with any actions taken and any reasons for not completing certain elements.

Component 3-FAST-M implementation Programme

The implementation program **(Figure 2)** consisted of the FAST-M training, selection of clinical champions, and feedback achieved from data collected during the baseline phase, and through the facility audit form.

The two-day interactive training sessions were delivered by healthcare experts after the completion of the baseline phase and before the implementation of the FAST-M bundle intervention. Instructions on the use of FAST-M bundle care tools were provided, and hands-on training of healthcare providers was conducted during the sessions.

Health care practitioners including doctors and nurses (n= 40) working in obstetrics and gynecology units, internal medicine, operating rooms, emergency, and intensive care units of LUMHS hospital were trained to record patients' vital signs on the MEOWS chart (heart rate, respiratory rate, oxygen saturation, blood

For peer review only - http://bmjopen.bmj.com/site/about/guidelines.xhtml

pressure, conscious level, urine output, temperature, and fetal heart rate (if applicable). These observations were then charted on a MEOWS chart in the inpatient setting and then inserted into the patient's files. Follow-up training sessions were also conducted every 2 weeks by the local team leads during the implementation of the FAST-M bundle intervention.

The local clinical champions and team leaders were identified and trained to take the lead in implementation of the FAST-M intervention at the study site The selected champions included doctors and nurses from different hospital departments primarily involved in the management of maternal sepsis patients including obstetrics and gynecology units, emergency department, internal medicine, and intensive care unit.

The overarching goal of each champion was to encourage engagement and compliance with the FAST-M bundle. To achieve this goal, champions at each unit were engaged in several key activities that included disseminating knowledge, advocating, navigating boundaries, facilitating consensus, arranging meetings with stakeholders, tracking quality indicators, and developing organisational communication strategies and relationships. The patient records collected were also counter-checked by the clinical champions of the assigned units. These champions also helped the study team in the identification of patients with suspected sepsis and their enrolment in the study and remained engaged throughout the implementation process.

In addition, training certificates were distributed among the participants. Moreover, FAST-M tools (MEOWS chart, decision, and treatment tool) were displayed in the form of posters throughout all the units involved in the management of maternal sepsis and implementation of FAST-M intervention.

Study Period:

The study comprised a baseline period of two months in which usual practices to detect and treat maternal sepsis existing locally were assessed. The FAST-M training was conducted on the completion of the baseline period. Following training, an intervention period of four months was commenced to assess any change in practice.

For peer review only

Outcomes:

Primary process outcomes included

1) The proportion of patients admitted with indications of sepsis, who received appropriate monitoring (full set of vital sign measurements on admission recorded on MEOWS chart).

2) The proportion of women with suspected maternal sepsis receiving the FAST-M treatment bundle (including each bundle component) within 1 hour of identification of sepsis.

Secondary outcomes included

1) Rate of vital signs with complete recording of MEOWS chart components [heart rate, respiratory rate, oxygen saturation, blood pressure, mental state, urine output, temperature, and fetal heart rate (in pregnant women)].

2) Rate of application of FAST-M bundle components [Fluids, Antibiotics, source identification (identified clinically) and control, assessment of the need to transport/ transfer to a high level of care, and ongoing monitoring (of the mother and neonate)].

3) Rate of maternal morbidity and mortality after FAST-M intervention.

Data Collection:

Quantitative data for this feasibility study was collected by a senior research assistant (SRA) on the facility record CRF 1 **(Supplement File 2)**, and patient notes including the MEOWS charts, FAST-M decision, and treatment tools onto the relevant paper-based CRFs 2 & 3 **(Supplemental File 3-4)** following patient consent **(Supplemental File 5).** Data were composed of various outcomes; structural, clinical, organisation, and any adverse events. The data were maintained in an investigator file to be secured in a locked cabinet. Anonymised and deidentified patients' information recorded on the data collection sheet were recorded in a database located on a secure server.

During the study, biweekly monitoring of the study site was undertaken by the research coordinator, to observe practice, collect feedback and provide updates on the study site's performance, and evaluate any additional training requirements.

For peer review only - http://bmjopen.bmj.com/site/about/guidelines.xhtml

Statistical Analysis:

Statistical Package for Social Sciences (SPSS) version 19.0 (IBM Corporation, Armonk, NY, USA) and STATA 14 computer software (Stata Corporation, Texas USA) was used for data processing and statistical analysis. Data were reported in frequency and percentages for categorical observations. A Chi-square test for independence was used to compare components of FAST-M bundle between phases (baseline and intervention). The Chi-square test for trend (P trend) was used to observe the performance during the intervention phase. Statistical significance was determined at $P \leq 0.05$.

Patient and public involvement:

Patients were not involved in the design of this study. The FAST-M intervention was implemented on the enrolled patients however, they were not invited to comment on the study design, interpret the results or contribute to writing or editing of this document for accuracy of findings. Hospital staff participated in the implementation but were not involved in the analysis of findings.

RESULTS:

Vital signs monitoring assessment: 439 women were included in the study (200 in baseline and 239 in the intervention phase), with vital signs recorded on admission ***(Table 1)***. 242/439 were cases of suspected maternal infection, and 138/242 were women with suspected maternal sepsis. Figure 1 provides the summary of the screening, and enrolment of the study participants. Following the FAST-M implementation, significant improvements were seen in the assessment of oxygen saturations in the intervention phase compared to the baseline phase [Difference: 74%; 95%CI:68.4%-80.5% p<0.01], fetal heart rate assessment [Difference:42.02%; 95%CI: 33.7% to 50.28%; p=<0.01], measurement of urine output [Difference 23.5%; 95%CI: 17.6% to 29.4%; p<0.01]. Temperature, respiratory rate, heart rate, and blood pressure were measured in 97% of the cases in the baseline phase but showed an increase of almost 3% in the intervention phase [Difference 2.5% 95%CI: 0.34% to 4.66%; p≤0.05]. No significant difference was found in the assessment of mental status in the intervention phase when compared to the baseline phase [Difference 1.6%; 95%CI: -0.52% to 3.7%; p=0.18] **(Supplemental File 6)**.

For peer review only

Table 1: Patient's assessment of vital signs on admission (n=439)

Vital Sign	Baseline n=200	Intervention n=239	P-Value
Respiratory rate	194(97%)	239(100%)	0.009
Oxygen Saturations	51(25.5%)	239(100%)	0.0005
Temperature	195(97.5%)	239(100%)	0.019
Heart rate	194(97%)	238(99.6%)	0.051
Systolic BP	196(98%)	239(100%)	0.042
Diastolic BP	196(98%)	239(100%)	0.042
Urine output	153(76.5%)	239(100%)	0.0005
Mental State	196(98%)	238(99.6%)	0.182
Fetal heart rate (in pregnant woman) *	80 (58%)	123 (100%)	0.0005

*For FHR the denominator is 138 in baseline and 123 in the intervention phase.

Baseline characteristics of women with suspected maternal sepsis: 242 women were identified by the FAST-M decision tool as fulfilling the criteria for suspected maternal infection, of which 65% (130/200) women were during the baseline and 46.9% (112/239) during the intervention phase. Out of 242 women with maternal infections, 138 (57%) were identified with suspected maternal sepsis. Sixty of 138 (46.2%) were during the baseline phase and 78 of 112 (69.6%) were observed during the intervention phase. The characteristics of the patients with suspected maternal sepsis and various laboratory investigations carried out for the identification of source of infection are presented in **(Supplemental File 7&8)**.

Compliance with the FAST-M treatment bundle within 1 hour: Women with suspected maternal sepsis received all the components of the FAST-M treatment bundle, with the goal of treatment within 1 hour. The mean start time of the FAST-M treatment bundle was 30.67±15.18 minutes for fluids, 26.43±13.45 for antibiotics administration, 53±12.72 minutes for investigation of the source of infection and its treatment, 19.62±16.26 to assess the need to transfer high level and 54.43±10.1 for monitoring. Compliance improved following the intervention, as compared to the baseline [70.5%; 95%CI: 60.4% to 80.6%; p<0.01] ***(Table 2)***. Improvements in the management of sepsis cases were seen in all FAST-M bundle components, women

For peer review only - http://bmjopen.bmj.com/site/about/guidelines.xhtml

were more likely to get IV fluids [52.2%; 95%CI: 38% to 66.3% ; p<0.01], antibiotics [24.5%; 95%CI: 12.3% to 36.6% ;p<0.001], source identification [27.8%; 95%CI: 15.3% to 40.3%; p<0.01], assessment of the need to transfer required to higher level of care [65.3%; 95%CI: 52.8% to 77.6% ;p<0.01], and ongoing monitoring [63.3% ; 95%CI: 50.3% to 76.4%; p<0.01] within an hour of suspected sepsis identification compared to the baseline phase **(Figure 3)**. The improvements in the management of women with suspected maternal sepsis were maintained throughout the intervention, with no significant decline in performance over time (P trend value >0.05) **(Supplemental file 9)**.

Table 2: Completion of FAST-M bundle within 1 hour of recognition of maternal sepsis

	Baseline (n=60)	Intervention FAST-M (n=78)	Intervention over time				P-Value	P-trend
			Month 1 (n=29)	Month 2 (n=18)	Month 3 (n=17)	Month 4 (n=14)		
**Total Bundle**	**0(0%)**	**55(70.5%)**	**20(69%)**	**9(50%)**	**15(88.2%)**	**11(78.6%)**	**0.0005**	**0.2069**
Fluids	21(35%)	68(87.2%)	24(82.8%)	17(94.4%)	15(88.2%)	12(85.7%)	0.0005	0.7516
Antibiotics	43(71.7%)	75(96.2%)	27(93.1%)	18(100%)	16(94.1%)	14(100%)	0.0001	0.3979
Source identification	41(68.3%)	75(96.2%)	28(96.6%)	17(94.4%)	17(100%)	13(92.9%)	0.0005	0.8405
Assessment of need to transfer high level	7(11.7%)	60(76.9%)	22(75.9%)	10(55.6%)	16(94.1%)	12(85.7%)	0.0005	0.1739
Monitoring	12(20%)	65(83.3%)	24(82.8%)	10(55.6%)	17(100%)	14(100%)	0.0005	0.0384

Maternal Outcomes: The facility data shows, 80 (28.6%) deaths due to maternal sepsis out of total 279 total maternal deaths recorded from the period of January to December 2021. **(Supplemental File 10)**. A 9.4% decline in the sepsis related maternal mortality was observed: 52/159 (32.7%) in the month of January-June and 28/120 (23.3%) during the July-December 2021 (p-value=0.087).

For peer review only - http://bmjopen.bmj.com/site/about/guidelines.xhtml

DISCUSSION

Main Findings:

The implementation of the FAST-M intervention was feasible, and effective in early identification, and management of maternal sepsis at the study setting. There was high compliance with all monitoring components after the intervention, with significant increase in compliance with completion of the MEOWS charts. Following the intervention, women with suspected sepsis were more likely to receive all the critical early interventions needed to treat maternal sepsis. There was high and sustained compliance during the post-interventional observation period with completion of the FAST-M treatment bundle within an hour once maternal sepsis was suspected.

Interpretation:

The use of the MEOWS chart has been related to improved health outcomes and has been shown to predict severe maternal morbidity and mortality. Though there is limited evidence on its feasibility and impact on outcomes in low resource settings.[27] The present study confirms the applicability of the MEOWS chart in a low resource setting in Pakistan. In the present study, we found that with the combined use of the MEOWS chart along with health care provider training, resulted in improvement in the monitoring of vital signs after the implementation of the FAST-M intervention.

In low-resource settings, the lack of screening processes and resources can be an obstacle to the early detection and treatment of maternal sepsis. [28,29] It was observed, that whilst the Royal College of Obstetricians and Gynaecologist guidelines (RCOG) were recommended for patient management and treatment at the LUMHS hospital, there was no standardised chart for the recording of patients' vital signs.

The MEOWS chart used in the Malawian settings,[21] was adapted before its implementation in Pakistan based on health care providers' suggestions during the adaptation phase of the study. Consequently, the health care providers were trained on an adapted MEOWS chart for regular recording of a full set of vital signs to screen patients who are at risk of developing sepsis, including the recording of oxygen saturations.

For peer review only

Providers showed a large improvement in the reliability of routine oxygen saturation recording following the intervention, so this component was shown to be a feasible addition in the Pakistan context.

Early detection of sepsis patients is critical for prompt treatment and improved maternal outcomes. [3,30,31] Research indicates that early warning scores accompanied by a clear escalation policy guide the healthcare providers on the need for additional action to optimise patient care.[31,32] The decision tool used guided the healthcare providers on when to proceed with treating the patient as having suspected sepsis and initiating the FAST-M bundle.[33,34] As noted earlier, the FAST-M screening tools in Malawi facilitated the identification of maternal sepsis patients and guided healthcare staff to escalate management plans.[21] The present study shows that it was also true in the Pakistan context where the FAST-M decision tool along with the MEOWS chart guided healthcare providers in appropriate screening and identification of women with suspected sepsis and assisted in making timely decisions for such cases.

The study was not powered to demonstrate reductions in maternal mortality or severe morbidity, but due to the critical importance of this outcome, it was pre-specified as a secondary outcome. Following intervention, we observed a 9.4% decline in mortality of maternal sepsis patients, which suggests that the early identification of the sepsis patients and their timely management may as hoped lead to improved clinical outcomes. This finding is consistent with evidence from high income countries where the use of sepsis care bundles for the immediate treatment of sepsis has been demonstrated in large observational and interventional studies to reduce mortality and improve patient's condition.[13, 35] It's been reported that each part of the care bundle should be completed to reach the desired result, and failure to complete any of the components may have an impact on the overall patient outcome.[32] In the Malawi study, the FAST-M bundle implementation did not demonstrate a reduction in maternal mortality, [21] so our mortality reduction finding in Pakistan is particularly important.

Fluid administration is considered an important part of early sepsis management. [20,36] We observed a significant improvement in timely fluid administration once the FAST-M bundle was implemented. The restoration of organ perfusion is an important goal of maternal sepsis treatment and fluid resuscitation

For peer review only - http://bmjopen.bmj.com/site/about/guidelines.xhtml

should seek to restore organ perfusion while maintaining hemodynamic stability.[32] However, the benefits and risks of intensive early goal-guided fluid resuscitation have been the subject of intensive research.[33-36] The bundle care tools we used guided health care providers with a recommended fluid administration approach and to cautiously reassess the patient's hemodynamic condition between fluid boluses, through clinical examination and monitoring of the patient's vital signs. This sought to avoid overloading the patient which can actually worsen sepsis outcomes. [37,38] Higher volume of fluids in patients with sepsis and septic shock has been associated with harm, although a meta-analysis showed that the quality of evidence was very low [38]. The CLASSIC pilot study showed that restrictive fluid therapy was feasible in the ICU setting and it improved the mortality outcomes in the restrictive group, although not significantly [39].

Fluid management in patients with maternal sepsis and pre-eclampsia is challenging due to the increased risk of pulmonary oedema and must therefore be particularly cautiously managed by healthcare professionals.[32] During the FAST-M implementation, clear teaching and guidance were provided to the healthcare providers for proper fluid administration in pre-eclamptic or eclamptic patients.

Antibiotics are considered the cornerstone of sepsis treatment, and studies have shown that timely administration of antibiotics improves patient outcomes and reduces mortality.[40,41] The risk of death from sepsis increases with each hour of antibiotic delay (OR 1.04 per hour; 95 percent CI 1.03–1.06; p 0.001).[40,41] As a result, empiric broad-spectrum antibiotics should be given as early as possible, and it is normally suggested that clinicians should aim to provide this where possible within 1 hour of sepsis detection.[42] In Malawi, 42.3% improvement was seen in intravenous antibiotics administration within an hour of sepsis detection, from a relatively low baseline performance of 25%.[21] It is possible that the 24.5 percent increase in the present study in timely initiation of antibiotic administration may have led to improved clinical outcomes of women with suspected maternal sepsis.

For peer review only

Source identification was improved by 27.8% after FAST-M implementation in the study setting. Similar findings were reported in Malawi, where source identification improved by 18.2% within an hour of sepsis detection after FAST-M intervention.[21]

In the baseline phase, it was observed that clinicians had reservations to advise additional tests to identify the source of infection due to the logistics of undertaking these investigations in the facility and potential additional costs to be incurred by the patients. Considering this, the health care providers were given guidance during the training sessions regarding the significance of further investigations if the source is not initially clear with the clinical history and examination. [32] Consequently, an increase was seen in measurements of blood cultures, imaging (abdominal, and chest), urine, and other blood tests in the intervention phase **(Supplemental File 8)**.

Amongst patients receiving treatment for maternal sepsis, the need for transfer to a higher level of care should be considered, and the decision on a patient's transfer made by a senior clinician as part of their review.[32] In Malawi, 43.9% improvement was seen in the consideration of transfer to the high level of care after the FAST-M intervention implementation.[21] During the training program, the importance of assessment of a patient for transfer within one hour of recognition of sepsis was highlighted, and emphasis was made on expediting the process if their vital signs were not improving after initial sepsis treatment. Following this, the assessment of need to transfer was improved by 65.3% within an hour of sepsis detection in the intervention period.

Overall, following the FAST-M intervention, more women were monitored and screened for maternal sepsis, and the complete FAST-M treatment bundle was administered within an hour in 70.5% of women with suspected sepsis. The study findings demonstrate that the FAST-M intervention was feasible and improved the quality of care and clinical outcomes when implemented in the context of a large government hospital in Pakistan. FAST-M has the potential to be used as an integrated approach for early recognition and management of maternal sepsis in low-resource health settings of Pakistan and other LMIC's. However,

For peer review only

as we found when translating the FAST-M tools from Malawi to Pakistan some modifications in the intervention were needed to ensure they were optimised for the local context and available resources.

Strengths and limitations:

The strength of our research is that it's the first feasibility study conducted for the assessment of a maternal sepsis care bundle in a low-resource setting within Pakistan. This approach provided the opportunity to analyse practical, and operational factors for introducing the FAST-M intervention in the local setting. The findings of this study can lead to optimisation of the bundle prior to the large-scale intervention trial. Also, this study adds further weight to Malawi work demonstrating the feasibility of the FAST-M bundle in different low resource settings.

However, the study has several limitations. Our research design does not account for possible temporal effects, hence is exposed to possible selection and reporting bias.[43] Although a before and after design couldn't identify the cause-and-effect relationship, this was the practical approach for determining the feasibility of the FAST-M intervention locally.

This study was conducted at one site only i.e., LUMHS which is a tertiary hospital that was comparatively better equipped than basic health units (BHUs) in the country to implement any care bundle approach. Hence, the intervention will next be implemented in other low-resource settings including BHU's across Pakistan. A large multi-country randomised trial is also underway to more robustly ascertain the effectiveness of the FAST-M bundle to improve maternal sepsis care and outcomes in LMICs [44]

Although the intervention was found to be feasible, we recognise that the intervention period was short. A further assessment period is required to examine how well the FAST-M intervention is sustained and if it becomes normalised as a routine part of clinical care in the longer term. This additional information has been collected via an embedded qualitative study and will be reported separately.

For peer review only

Additionally, the high influx of patients and shortage of health care staff in LUMHS could have led to irregular monitoring which might have resulted in limited screening of the patients. Some of the women with suspected sepsis may have been categorised as maternal infection cases, which might have restricted the ability to demonstrate the differences between intervention and baseline phase.

Some of the referred patients in the baseline phase were observed to be already on fluid and antibiotics before admission to the hospital, which could have limited the ability of a before and after comparison, though it was only 9.4% (13/138) of patients in the baseline phase who were noted to have already received fluids and antibiotics. Additionally, the health care providers might have been more compliant with the FAST-M intervention, and the outcomes of this study therefore better than one would see if such an intervention was rolled out at scale due to the close monitoring of the site during the conduct of the study.

CONCLUSION

Implementation of the FAST-M intervention was considered feasible at the LUMHS hospital. The long-term vision is that the intervention will be trialled in other settings across Pakistan, based on the outcomes of this feasibility study, which will help to reduce the high rate of maternal deaths caused by sepsis in the country.

Acknowledgments: We would like to acknowledge the healthcare providers from the LUMHS hospital Dr Prince Akash, Dr Aqsa, Dr Batool, Dr Sana, Dr Qurat ul ain, Dr. Sahiba, and staff nurse Tomsina who served as FAST-M champions and actively took on their role in implementing the FAST-M bundle intervention. We would also like to acknowledge Ms. Ruqaiya Bano for data collection of this study and Ms Maliha Fazal for editing the manuscript.

Contributors: DL and SI designed the study and its processes. RS, FP, NM and SK provided support in data collection. AR, SI GK, BK, AJ and DM carried out the data analysis and interpretation. SI, GK, AR, and BK took the lead in writing the manuscript. SI takes responsibility for the overall content as the guarantor. AC, DL, CD, JC, LS, RS, RB, SSH SS, FP reviewed the manuscript and provided feedback on the article.

For peer review only

Funding: This research was commissioned by the National Institute for Health Research (NIHR). Award Ref: NIHR300808 Host: University of Liverpool. The views expressed in this paper are those of the authors and not necessarily those of the NIHR or the Department of Health and Social Care. The project was also supported by HRP Alliance for Research Capacity Development in Sexual and Reproductive Health, University of Birmingham, and Aga Khan University Research Funds.

Competing interests: Declared none.

Patient and public involvement: Patients were not involved in planning the design of this study. The FAST-M intervention was implemented on the enrolled patients however, they were not invited to comment on the study design, interpret the results or contribute to writing or editing of this document for accuracy of study findings. Hospital staff participated in the implementation but were not involved in the analysis of findings for accuracy and readability.

Patient consent for publication: Not applicable.

Ethics approval: Ethical approval for this study was obtained from the LUMHS hospital [REC/-886, 4-87], Aga Khan University Ethical Review Committee [2019-2061-7102], and National Bioethics Committee [515/20/].

Provenance and peer review: Not commissioned; externally peer reviewed.

Data availability statement: The datasets were collected and analysed and can be made available from the corresponding author on reasonable request.

ORCID iDs:

For peer review only

SI: 0000-0002-8391-8559

GK: 0000-0003-3224-730X

RS: 0000-0002-8015-5744

AR: 0000-0003-3970-8236

BK: 0000-0002-8141-9203

FP: 0000-0002-7820-6565

SK: 0000-0002-9655-378X

NM: 0000-0002-5174-8671

AJ: 0000-0002-6674-8543

DM:0000-0002-3396-3087

SS: 0000-0003-1301-5239

JC: 0000-0002-0967-7320

CD: 0000-0002-4792-9496

RB: 0000-0002-7377-9524

LS: 0000-0002-7694-3561

SS: 0000-0002-6797-8631

AC: 0000-0002-3261-9807

DL: 0000-0002-7878-232

For peer review only

References:

1. Bonet M, Pileggi V, Rijken MJ, Coomarasamy A, et al. Towards a consensus definition of maternal sepsis: results of a systematic review and expert consultation. *Reprod Health* 2017;14:67.

2. Say L, Chou D, Gemmill A, et al. Global causes of maternal death: a WHO systematic analysis. *Lancet Global Health*. 2014 Jun 1;2(6):e323-33.

3. Kourtis AP, Read JS, Jamieson DJ. Pregnancy and infection. *N Engl J Med*. 2014;370(23):2211–8.

4. Vasudevan R. Urinary tract infection: an overview of the infection and the associated risk factors. *J Microbiol Exp*. 2014;1(2):42–54.

5. Bonet M, Brizuela V, Abalos E, et al. Frequency and management of maternal infection in health facilities in 52 countries (GLOSS): a 1-week inception cohort study. *Lancet Global Health.* 2020 May 1;8(5):e661-71

6. Vos T, Lim SS, Abbafati C, et al. Global burden of 369 diseases and injuries in 204 countries and territories, 1990–2019: a systematic analysis for the Global Burden 2019. *Lancet*. 2020;396(10258):1204–22

7. Rudd KE, Johnson SC, Agesa KM, et al. Global, regional, and national sepsis incidence and mortality, 1990–2017: analysis for the Global Burden of Disease Study. *Lancet.*2020; 395: 200–11.

8. World Health Organization. Trends in maternal mortality: 1990 to 2015: estimates from WHO, UNICEF, UNFPA, World Bank Group and the United Nations Population Division: executive summary. (apps.who.int/iris/handle/10665/193994) Accessed on 1st Sept 2022

9. Hanif M, Khalid S, Rasul A, et al. Maternal mortality in rural areas of Pakistan: challenges and prospects. *Rural Heal*. 2021 Jun 7;27:1040-7.

10. Fikree FF, Ali T, Durocher JM, et al. Health service utilization for perceived postpartum morbidity among poor women living in Karachi. *Social science & medicine*. 2004 Aug 1;59(4):681-94.

11. National Institute of Population Studies (NIPS), ICF International (2013) Pakistan Demographic and Health Survey 2012-13. NIPS and ICF International, Islamabad and Calverton. - References - Scientific Research Publishing (www.scirp.org/(S(351jmbntvnsjt1aadkposzje))/reference/referencespapers.aspx?referenceid=2849234) Accessed on 1st Sept 2022

12. Reinhart K, Daniels R, Kissoon N, et al. Recognizing sepsis as a global health priority – A WHO resolution. *N Engl J Med* 2017;377:414–7.

For peer review only

13. Damiani E, Donati A, Serafini G, et al. Effect of performance improvement programs on compliance with sepsis bundles and mortality: a systematic review and meta-analysis of observational studies. *PloS one*. 2015 May 6;10(5):e0125827.

14. Levy MM, Dellinger RP, Townsend SR, et al. The Surviving Sepsis Campaign: results of an international guideline-based performance improvement program targeting severe sepsis. *Crit Care Med* 2010;38(2):367–74.

15. Evans L, Rhodes A, Alhazzani W, et al. Surviving sepsis campaign: international guidelines for management of sepsis and septic shock 2021. *Int care med*. 2021 Nov;47(11):1181-247.

16. Nutbeam T, Daniels R, Keep J, UK Sepsis Trust. Toolkit: Emergency Department management of Sepsis in adults and young people over 12 years- 2016. The Royal College of Emergency Medicine; National Institute for Health and Care Excellence (NICE); 2016.

17. Abdu M, Wilson A, Mhango C, et al. Resource availability for the management of maternal sepsis in Malawi, other low-income countries, and lower-middle-income countries. *Int J Gynecol Obstet* 2018;140:175–83.

18. Baelani I, Jochberger S, Laimer T, et al. Availability of critical care resources to treat patients with severe sepsis or septic shock in Africa: a self-reported, continent wide survey of anaesthesia providers. *Crit Care* 2011;15:R10.

19. Bataar O, Lundeg G, Tsenddorj G, et al. Nationwide survey on resource availability for implementing current sepsis guidelines in Mongolia. *Bull World Health Organ* 2010;88:839–46.

20. Lissauer D, Cheshire J, Dunlop C, et al. Development of the FAST-M maternal sepsis bundle for use in low-resource settings: a modified Delphi process. *BJOG-INT J OBS*. 2020 Feb;127(3):416-23

21. Cheshire J, Jones L, Munthali L, et al. The FAST-M complex intervention for the detection and management of maternal sepsis in low-resource settings: a multi-site evaluation. *BJOG-INT J OBS*. 2021 Feb 4.

22. Reinhart K, Kissoon N. Sepsis Guidelines for Pakistan. *Anaesth Pain & intensive Care*. 2015 Apr 1;19(2):105-7.

23. Ahmed SI, Sikandar R, Barolia R, et al.Evaluation of the feasibility of the FAST-M maternal sepsis intervention in Pakistan: a protocol. *Pilot and feasibility studies*. 2022 Dec;8(1):1-2.

For peer review only

24. Ahmed SI, Khowaja BMH, Barolia R, et al. Adapting the FAST-M maternal sepsis intervention for implementation in Pakistan: a qualitative exploratory study. *BMJ Open* 2022;12:e059273.

25. Umar A, Ameh CA, Muriithi F, et al. Early warning systems in obstetrics: a systematic literature review. *PLoS One* 2019;14: e0217864.

26. Downey CL, Tahir W, Randell R, et al. Strengths and limitations of early warning scores: A systematic review and narrative synthesis.*I nt J Nurs Stud* 2017;76:106–19.

27. Pakenham-Walsh N, Bukachi F. Information needs of health care workers in developing countries: a literature review with a focus on Africa. *Hum Resour Health* 2009;7:30.

28. Papali A, McCurdy MT, Calvello EJB. A 'three delays' model for severe sepsis in resource-limited countries. *J Crit Care* 2015;30:861.e9– 861.e14.

29. Gatewood MO, Wemple M, Greco S, et al. A quality improvement project to improve early sepsis care in the emergency department. *BMJ Qual Saf* 2015;24:787–95.

30. Hayden GE, Tuuri RE, Scott R, et al. Triage sepsis alert and sepsis protocol lower times to fluids and antibiotics in the ED. *Am J Emerg Med* 2016;34:1–9.

31. Seymour CW, Gesten F, Prescott HC, et al. Time to treatment and mortality during mandated emergency care for sepsis. *N Engl J Med* 2017;376:2235–44.

32. Cheshire J, Dunlop C, et al, Glob. libr. women's med., ISSN: 1756-2228; DOI 10.3843/GLOWM.413113

33. The Uk Sepsis Trust. Clinical Professional Resources. 2018.

34. Suleiman I, Vousden N, Shennan AH. Recognition and Management of Maternal Sepsis in Low and Middle-Income Countries: What do we know and where are the Gaps? Open access. *Glob Womens Heal* 2018;1(1):1–5.

35. Rivers E, Nguyen B, Havstad S, et al. Early Goal-Directed Therapy in the Treatment of Severe Sepsis and Septic Shock.*N Engl J Med* 2001;345(19):1368–77.

36. Mouncey PR, Osborn TM, Power GS, et al. Trial of Early, Goal-Directed Resuscitation for Septic Shock. *N Engl J Med* 2015;372(14):1301–11.

37. RISE Investigators, ANZICS Clinical Trials Group, Peake SL, Delaney A, Bailey M, Bellomo R,e t al. Goal-Directed Resuscitation for Patients with Early Septic Shock. *N Engl J Med* 2014;371(16):1496–506.

For peer review only

38. Angus DC, Yealy DM, Kellum JA. Protocol-based care for early septic shock. *N Engl J Med*. 2014 Jul 1;371(4):386-.

39. Meyhoff TS, Hjortrup PB, Wetterslev J, Sivapalan P, Laake JH, Cronhjort M, Jakob SM, Cecconi M, Nalos M, Ostermann M, Malbrain M. Restriction of intravenous fluid in ICU patients with septic shock. New England Journal of Medicine. 2022 Jun 30;386(26):2459-70.

40. de Oliveira FSV, Freitas FGR, Ferreira EM, *et al.* Positive fluid balance as a prognostic factor for mortality and acute kidney injury in severe sepsis and septic shock. *J Crit Care* 2015;30(1):97–101.

41. Liu VX, Fielding-Singh V, Greene JD, et al. The Timing of Early Antibiotics and Hospital Mortality in Sepsis.*Am J Respir Crit Care Med* 2017;196(7):856–63.

42. Rhodes A, Evans LE, Alhazzani W, et al. Surviving Sepsis Campaign: International Guidelines for Management of Sepsis and Septic Shock: 2016. *Int Care Med* 2017;43(3):304–77.

43. Songer T. Before and after studies in injury research. Aufgerufen am. 2008; 13:2015.

44. Lindiwe. 2020 MLW Annual report is released! [Internet]. Malawi Liverpool Wellcome Trust Clinical Research Program. 2021 [cited 2023Feb2]. Available from: https://www.mlw.mw/2020-mlw-annual-report-is-released/

Figure Legends

Figure 1: Flow of participants through the study

Figure 2: Components of FAST-M Intervention

Figure 3: Completion of FAST-M bundle within 1 hour of recognition of maternal sepsis

For peer review only

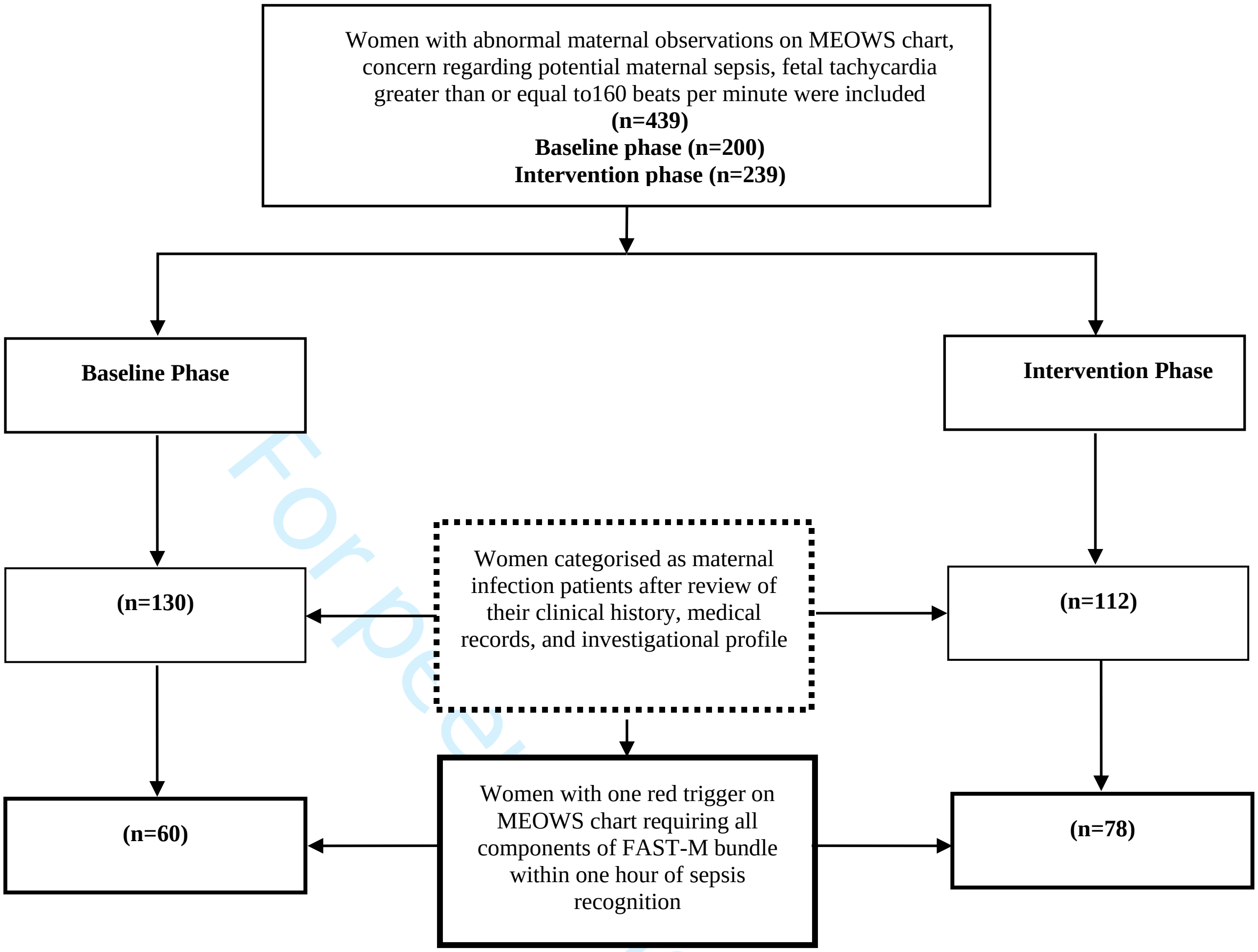

Figure 1: Flow of participants through the study

For peer review only

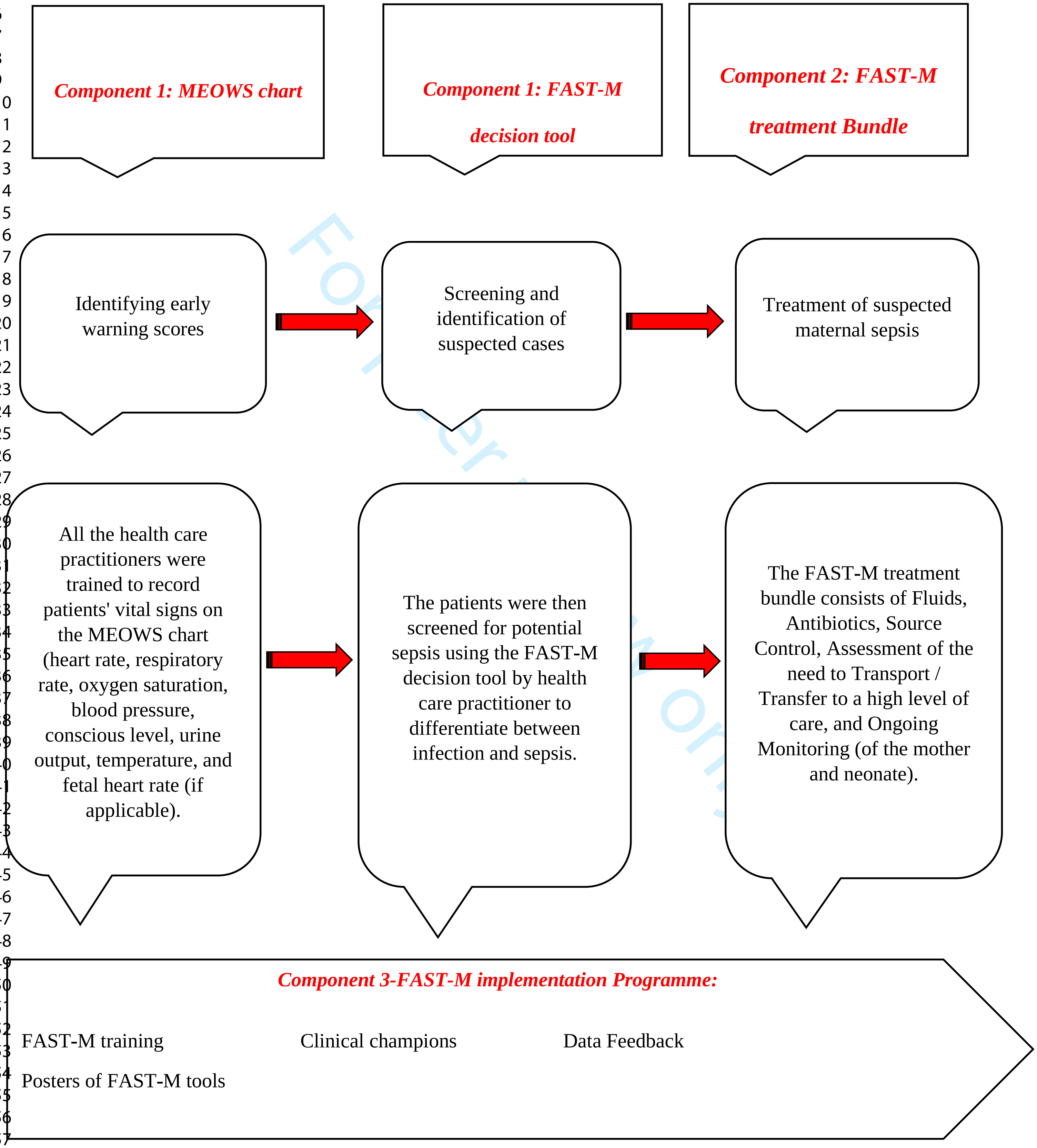

Figure 2: Components of FAST-M Intervention

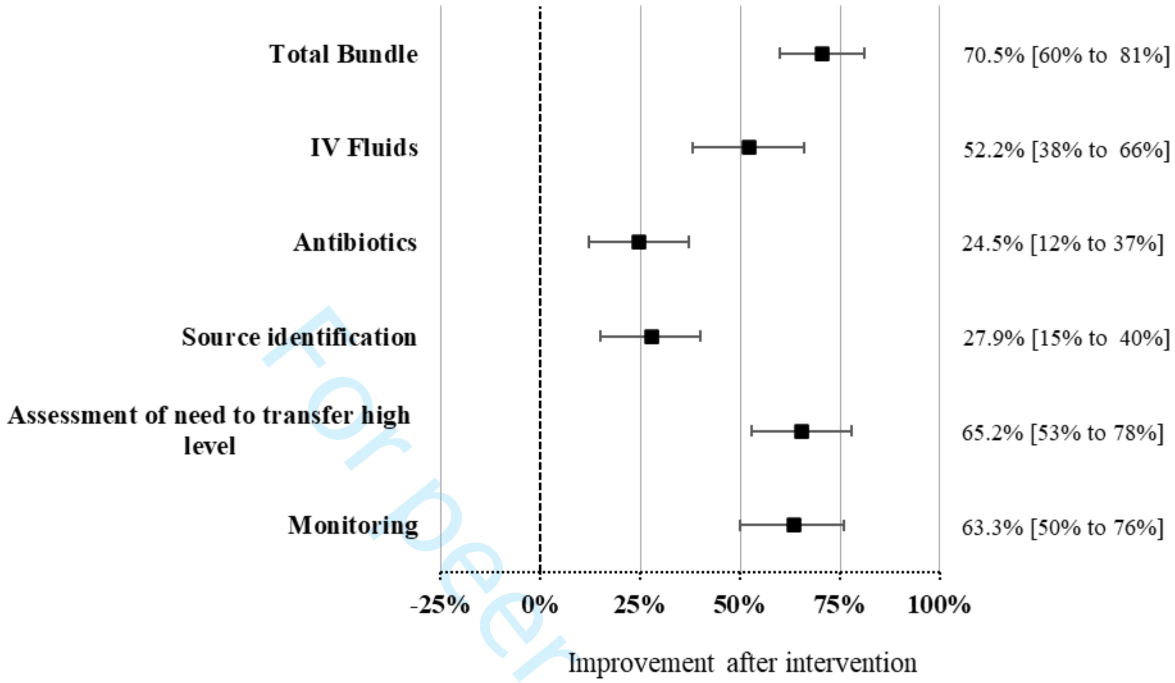

Figure 3: Completion of FAST-M bundle within 1 hour of recognition of maternal sepsis

For peer review only

MODIFIED EARLY OBSTETRIC WARNING CHART (MEOWS CHART)

FAST-M

Patient		Patient ID		DOB/Age	

Contact clinical decision maker if patient triggers **ONE RED** or **TWO YELLOW** flags at any one time.

WRITE **VALUES** IN BOXES PROVIDED

		Date															
		Time															
		Initials															
**Respiratory rate** (breaths per minute)	25 or more	RED															
	21 - 24	YELLOW															
	11 - 20	NORMAL															
	10 or less	RED															
**Oxygen saturations** (%)	95 or more	NORMAL															
	94 or less OR needing oxygen	RED															
**Temperature** (°c)	38 or more	YELLOW															
	36.0 to 37.9	NORMAL															
	35.9 or less	YELLOW															
**Heart rate** (beats per minute)	120 or more	RED															
	100 - 119	YELLOW															
	50 - 99	NORMAL															
	40 - 49	YELLOW															
	39 or less	RED															
**Systolic blood pressure** (mmHg)	160 or more	RED															
	140 -159	YELLOW															
	100 -139	NORMAL															
	90 - 99	YELLOW															
	89 or less	RED															
**Diastolic blood pressure** (mmHg)	110 or more	RED															
	90 -109	YELLOW															
	40 - 89	NORMAL															
	39 or less	RED															
**Urine output** Hours since patient passed urine (tick box)	12 hours or less	NORMAL															
	12 - 18 hours	YELLOW															
	18 hours or more OR less than 0.5 ml/kg/hour	RED															
**Mental State** (tick box)	Alert	NORMAL															
	Not Alert	RED															
**Looks unwell** (tick box)	No	NORMAL															
	Yes	YELLOW															
**TOTAL YELLOW FLAGS**																	
**TOTAL RED FLAGS**																	
ACTION TAKEN (IF REQUIRED) Yes (Y) / No (N)																	

ACT NOW if patient triggers ONE RED or TWO YELLOW flags at any time. Escalate to clinical decision maker and start FAST-M decision tool.

UNITED AGAINST MATERNAL SEPSIS

THINK SEPSIS, ACT FAST-M

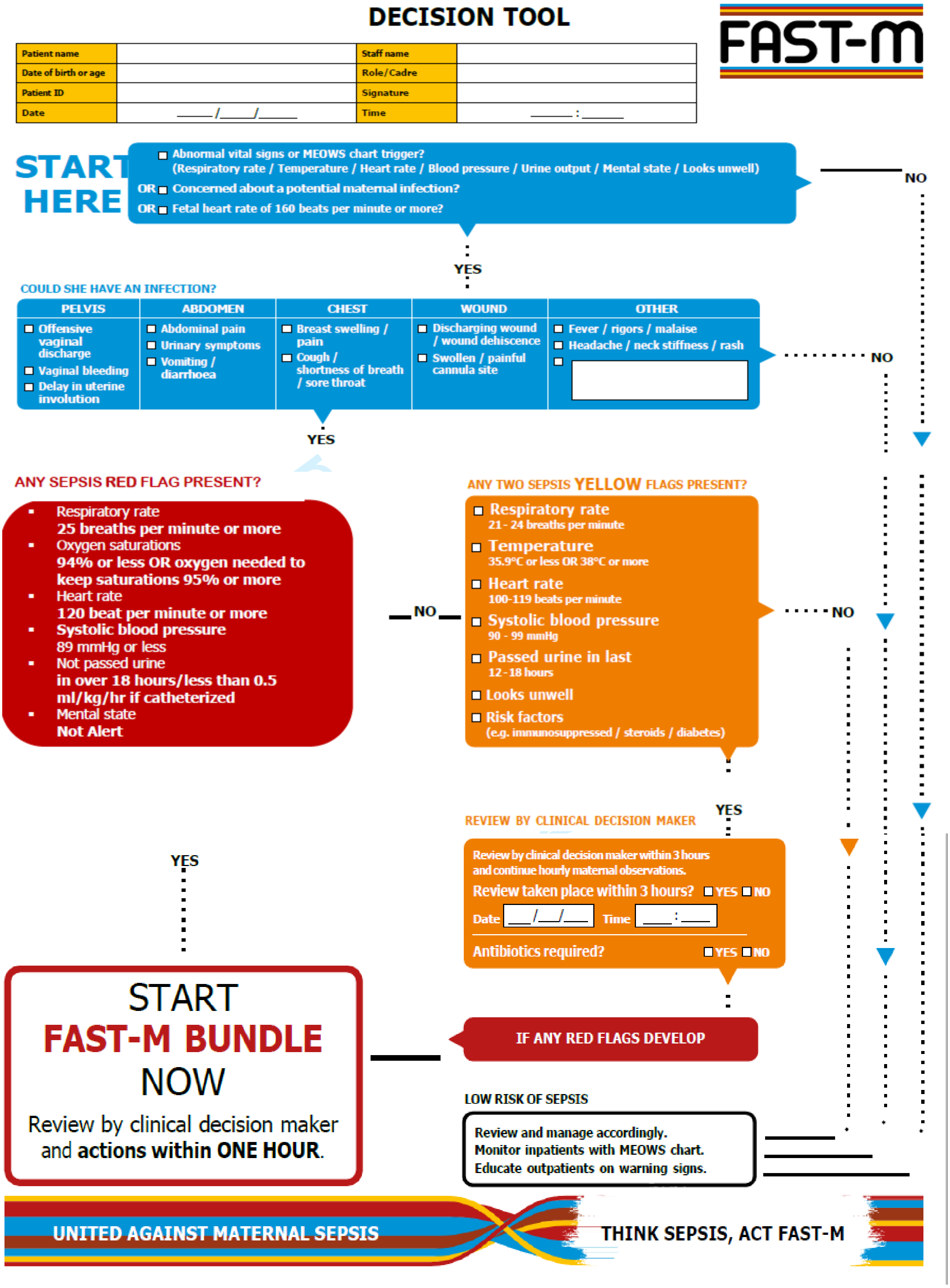
DECISION TOOL
FAST-M
Patient name
Date of birth or age
Patient ID
Date ____/____/____
Staff name
Role/Cadre
Signature
Time ____ : ____
START HERE
Abnormal vital signs or MEOWS chart trigger?
(Respiratory rate / Temperature / Heart rate / Blood pressure / Urine output / Mental state / Looks unwell)
OR Concerned about a potential maternal infection?
OR Fetal heart rate of 160 beats per minute or more?
NO
YES
COULD SHE HAVE AN INFECTION?
PELVIS
Offensive vaginal discharge
Vaginal bleeding
Delay in uterine involution
ABDOMEN
Abdominal pain
Urinary symptoms
Vomiting / diarrhoea
CHEST
Breast swelling / pain
Cough / shortness of breath / sore throat
WOUND
Discharging wound / wound dehiscence
Swollen / painful cannula site
OTHER
Fever / rigors / malaise
Headache / neck stiffness / rash
NO
YES
ANY SEPSIS RED FLAG PRESENT?
Respiratory rate
25 breaths per minute or more
Oxygen saturations
94% or less OR oxygen needed to keep saturations 95% or more
Heart rate
120 beat per minute or more
Systolic blood pressure
89 mmHg or less
Not passed urine
in over 18 hours/less than 0.5 ml/kg/hr if catheterized
Mental state
Not Alert
NO
ANY TWO SEPSIS YELLOW FLAGS PRESENT?
Respiratory rate
21 - 24 breaths per minute
Temperature
35.9°C or less OR 38°C or more
Heart rate
100-119 beats per minute
Systolic blood pressure
90 - 99 mmHg
Passed urine in last
12 - 18 hours
Looks unwell
Risk factors
(e.g. immunosuppressed / steroids / diabetes)
NO
YES
REVIEW BY CLINICAL DECISION MAKER
Review by clinical decision maker within 3 hours and continue hourly maternal observations.
Review taken place within 3 hours? YES NO
Date ___/___/___ Time ___ : ___
Antibiotics required? YES NO
YES
START
FAST-M BUNDLE
NOW
Review by clinical decision maker and actions within ONE HOUR.
IF ANY RED FLAGS DEVELOP
LOW RISK OF SEPSIS
Review and manage accordingly.
Monitor inpatients with MEOWS chart.
Educate outpatients on warning signs.
UNITED AGAINST MATERNAL SEPSIS
THINK SEPSIS, ACT FAST-M

TREATMENT BUNDLE

FAST-M

REMEMBER TO COMPLETE THESE ACTIONS WITHIN ONE HOUR

Patient name			Staff name		
D.O.B or age			Role/Cadre		
Patient ID			Signature		
Date & time of red flag observation	__/__/__ __:__	Date & time bundle started	__/__/__ __:__	Date & time of review by clinical decision maker	__/__/__ __:__

F — FLUIDS (caution in pre-eclampsia, severe anaemia and pulmonary oedema)

Date	___/___/___	Time fluids initiated	___:___	Initials		Give 500 ml crystalloid immediately. Repeat 500 ml boluses to a maximum of 30 ml/kg if hypotension persists.
Details / reason not completed						

A — ANTIBIOTICS

Date	___/___/___	Time started	___:___	Initials		See antibiotic guidelines below
Details / reason not completed						

S — SOURCE – identify and treat the source of infection

Date	___/___/___	Time considered	___:___	Initials		See source identification and treatment boxes below
Details / reason not completed						

T — TRANSPORT (to higher level hospital or location within hospital, if required)

Date & time transport considered	___/___/___ ___:___	Initials		Transport Required ☐ YES ☐ NO
Date & time transport requested	___/___/___ ___:___	Initials		☐ N/A
Date & time patient left facility	___/___/___ ___:___	Initials		
Destination				
Reason for any delay				

M — MONITORING (start MEOWS chart if not already started. Repeat observations every 30 minutes until otherwise decided by clinical decision maker)

Date & time monitoring commenced	___/___/___ ___:___	Details / reason not completed
Maternal / fetal monitoring should include	• Respiratory rate<br>• Oxygen Saturations<br>• Temperature<br>• Heart rate<br>• Blood pressure<br>• Urine output<br>• Mental state<br>• Fetal heart rate	
Neonatal monitoring and review commenced	☐ YES ☐ NO ☐ N/A	

ANTIBIOTIC GUIDELINES

Insert local guidance here

Immediate treatment for Maternal Sepsis:

- Ceftriaxone 2 g IV once daily (if no IV access this can be given as 2 IM injections of 1 g in different sites).
- If possible intra-abdominal source add Metronidazole 500 mg IV three times daily or 400 mg PO three times daily.

If above antibiotic regime is not available then give:

- Tazobactam 4.5 g IV daily two time a day
- Meropenem 1 g IV daily two times a day

IDENTIFY THE SOURCE

Consider

- Clinical history
- Clinical examination
- Blood tests (if available) (FBC, U&Es, LFTs, CRP, clotting)
- Blood cultures
- HIV and Malaria testing
- Urine sample
- Swabs (wound, vagina, throat)
- Sputum sample
- Imaging (abdominal, chest)
- Lumbar puncture
- Other ____________

REMOVE / TREAT THE SOURCE

Consider

- Malaria treatment
- Consider delivery of baby
- Removal of retained products of conception
- Debridement of wound / drainage of collection
- Removal of infected cannula / line
- Hysterectomy
- Targeted antibiotics once source known

UNITED AGAINST MATERNAL SEPSIS

THINK SEPSIS, ACT FAST-M

FORM 1: FACILITY AUDIT

FACILITY ID [ ][ ] – [ ][ ][ ]

Facility ID e.g. DH, KH *Visit ID e.g. 001, 002 etc.*

Today's Date: d d – m m – y y y y Time: h h : m m

Date previous form completed (or if first Visit, date study opened at this site): d d – m m – y y y y

Are you collecting the data during? **Baseline** ☐ **Intervention** ☐

3. How many of the following **MATERNAL OUTCOMES** have you had since the last visit or if first time since last 6 months?

Maternal Outcome	Number
Maternal Sepsis	
Maternal Deaths	
Post-Partum haemorrhage (>1L)	
Ante-Partum haemorrhage (>50ml)	
Severe pre-eclampsia/eclampsia (>160/110 and >2+protein in urine)	
Blood transfusions	
Uterine rupture	
Patient with reduced conscious level	
Injury (e.g. uterine, bladder, bowel)	
Anaphylaxis	
Pulmonary oedema	
Return to theatre	
Admission to ICU/HDU	
Broken down wounds (e.g. c/s, perineal)	

4. How many of the following **NEONATAL OUTCOMES** have you had since the last visit?

Neonatal Outcome	Number
Live Births	
Neonatal deaths before discharge from hospital	
Babies requiring antibiotics	
Sill births	
Admission to nursery care	
Apgar score of less than 7 at 5 mins after birth	

For peer review only - http://bmjopen.bmj.com/site/about/guidelines.xhtml

FORM 1: FACILITY AUDIT

FACILITY ID [ ][ ] – [ ][ ][ ]

Facility ID e.g. DH, KH *Visit ID e.g. 001, 002 etc.*

5. How many of the following **RESOURCES** are available today?

Resource	Amount			
	BAGS			
**Type of IV Fluids:**	0	1-5	6-10	>10
A) 0.9% Saline	☐	☐	☐	☐
B) Ringers Lactate	☐	☐	☐	☐
C) 5% dextrose	☐	☐	☐	☐
D) 50% dextrose	☐	☐	☐	☐

	Type of oral antibiotics			
**Type of oral antibiotics**	0	1-5	6-10	>10
A) Amoxicillin	☐	☐	☐	☐
B) Augmentin	☐	☐	☐	☐
C) Cephalosporin (e.g. Cefalexin)	☐	☐	☐	☐
D) Cefixime	☐	☐	☐	☐
E) Ciprofloxacin	☐	☐	☐	☐
F) Doxycycline	☐	☐	☐	☐
G) Clindamycin	☐	☐	☐	☐
H) Chloramphenical	☐	☐	☐	☐
I) Erythromycin	☐	☐	☐	☐
J) Metronidazole	☐	☐	☐	☐
K) Other (please state):	☐	☐	☐	☐

	VIALS			
**Type of IM / IV antibiotics**	0	1-5	6-10	>10
A) Amoxicillin	☐	☐	☐	☐
B) Ampicillin	☐	☐	☐	☐
C) Benzyl-penicillin	☐	☐	☐	☐
D) Penicillin G	☐	☐	☐	☐
E) Cefazolin	☐	☐	☐	☐
F) Cephalosporin (e.g. ceftriazone etc.)	☐	☐	☐	☐
G) Chloramphenical	☐	☐	☐	☐
H) Ciprofloxacin	☐	☐	☐	☐
I) Clindamycin	☐	☐	☐	☐
J) Erythromycin	☐	☐	☐	☐
K) Gentamycin	☐	☐	☐	☐
L) Metronidazole	☐	☐	☐	☐
M) Vancomycin	☐	☐	☐	☐
N) Other (please state)	☐	☐	☐	☐

	KITS			
**Manual vacuum aspiration (MVA) kits ready for use:**	0	1-5	6-10	>10
	☐	☐	☐	☐

* Good availability defined as a supply that is unlikely to run out before the next anticipated delivery

For peer review only

FORM 1: FACILITY AUDIT

FACILITY ID ☐☐ – ☐☐☐

Facility ID e.g. DH, KH *Visit ID e.g. 001, 002 etc.*

**Resource**	**Availability**		
	**Good ***	**Limited**	**None**
Equipment for IV line (cannula, dressings etc)	☐	☐	☐
Malaria tests	☐	☐	☐
Syphilis test	☐	☐	☐
HIV test	☐	☐	☐
Functioning theatre and staff able to remove source of infection	☐	☐	☐
Working thermometers	☐	☐	☐
Working BP machines	☐	☐	☐
Working O2 saturation machines(Pulse Oximeters)	☐	☐	☐
Fetoscopes / Pinnards/ fetal stethoscopes	☐	☐	☐
Clocks/watches	☐	☐	☐
Spare batteries	☐	☐	☐
Wound swabs	☐	☐	☐
Corona virus swabs	☐	☐	☐
Urine dips	☐	☐	☐
Sputum cultures	☐	☐	☐
Blood cultures	☐	☐	☐
Pregnancy tests	☐	☐	☐
Haematology blood tests	☐	☐	☐
Biochemistry blood tests	☐	☐	☐
Corona virus tests	☐	☐	☐
Blood taking equipment	☐	☐	☐
Functioning radiology department	☐	☐	☐
Functioning ultrasound machine and person to scan	☐	☐	☐
Functioning theatre and staff able to remove source of infection	☐	☐	☐
Weighing scales	☐	☐	☐
Urinary catheters	☐	☐	☐
Gloves	☐	☐	☐
Aprons	☐	☐	☐

For peer review only

6. How many of the following **STAFF** is available TODAY?

**Resource**	**Number**
Doctors	
Medical Officers	
Medical Students	
Registered Nurse	
Nursing students	
Hospital and Ward Attendants	

FORM 1: FACILITY AUDIT

FACILITY ID [ ][ ] – [ ][ ][ ]

Facility ID e.g. DH, KH *Visit ID e.g. 001, 002 etc.*

7. Are the following **FACILITIES** in working order TODAY?

Resource	Yes	No
Does the ward have electricity?		
Does the ward have running water?		
Does the ward have soap?		
Does the ward have alcohol gel?		
Are the autoclaves working?		
Are the washing facilities for staff available?		
Are the washing facilities for patients available?		
Are flushable toilet facilities for staff available?		
Are flushable toilet facilities for patients available?		

* Good availability defined as a supply that is unlikely to run out before the next anticipated delivery

Completed by: ____________________

Role: ____________________

Signature: ____________________

Date: *DD* / *MMM* / *YYYY*

You must have signed the Site Signature & Delegation Log

For peer review only

FORM 2: PATIENT CARE AUDIT FORM

Patient ID Number: ☐☐-☐☐☐

Facility ID e.g. A01, A02 etc *Unique ID e.g. 001, 002 etc*

Baseline ☐ **or** Intervention ☐

Date of audit: d d - m m - y y y y Time of audit: h h : m m

1. Clinical status at admission:

Antenatal ☐ Miscarriage ☐

Post-natal ☐ Termination of pregnancy ☐

2. Which of the following observations were recorded when the patient was first assessed?

	Yes	No	Not recorded
**Respiratory rate**			
**Saturations**			
**Temperature**			
**Heart rate**			
**Systolic BP**			

	Yes	No	Not recorded
**Diastolic BP**			
**Urine output**			
**Mental State**			
**Fetal heart rate**			

3. Were the observations recorded on a chart?

Yes ☐ No ☐ N/A ☐

→ If intervention phase, were they recorded on a MEOWS chart?
Yes ☐ No ☐

Page 1 of 2

For peer review only

FORM 2: PATIENT CARE AUDIT FORM **PATIENT ID NUMBER:** ☐☐☐ – ☐☐☐☐

4. Has the patient been admitted for the last 24 hours

Yes ☐ No ☐

If yes, go to question 5, if No got to Question 6

5. During the last 24 hours, please state the number of times each observation has been taken and charted:

	Number of times
Respiratory rate	☐
Oxygen saturation	☐
Temperature	☐
Heart rate	☐
Systolic BP	☐

	Number of times
Diastolic BP	
Urine output	
Mental state	
Fetal heart rate	

REVIEW EVERY PATIENT'S NOTES WITH AN ABNORMAL OBSERVATION AND CONSIDER COMPLETING FORM 3 IF THERE IS CONCERN ABOUT MATERNAL INFECTION

Completed by: ______________________

Role: ______________________

Signature: ______________________ Date: DD / MMM / YYYY

You must have signed the Site Signature & Delegation Log

For peer review only

FORM 3: INDIVIDUAL PATIENT FORM

Screen the notes for every patient that had a suspected infection.
Only complete this form for patients that fulfil the criteria in q1-4 below.

Also complete this form for any patient you observe during the patient care audit (Form2) that fulfils the criteria below.

CONSIDER QUESTIONS 1-4 TO DETERMINE IF THIS FORM NEEDS TO BE COMPLETED

1. Was there a concern about a potential maternal infection?

   Yes ☐ No ☐ → **DO NOT COMPLETE FORM**

   Date and time suspected: d d - m m - y y y y h h : m m

2. Have observations taken?

   Yes ☐ No ☐ → **COMPLETE FORM**

3. Was there a fetal tachycardia (heart rate) greater than or equal to 160 bpm?

   Yes ☐ No ☐ Not Recorded ☐

4. Did the patient have abnormal vital signs or MEOWS chart trigger - 1 or more red flags or 2 or more yellow flags?

   Yes ☐ No ☐

IF NO TO BOTH QUESTIONS, DO NOT COMPLETE FORM

For peer review only

FORM 3: INDIVIDUAL PATIENT FORM

Patient ID number: ☐☐☐☐ ☐☐☐☐

Date of assessment medical record: d D m m y y y y

Time of assessment medical record: ☐☐ ☐☐

5. **Study phase:**

Baseline ☐ Intervention ☐

6. At the time of assessment for maternal infection by the health care staff, were they an inpatient or outpatient?

Inpatient ☐ Outpatient ☐

→ Date of admission: d d - m m - y y y y

→ Whilst an inpatient, was a chart used for the patient? FAST-M MEOWS chart: ☐ Other chart ☐

If no, why not? ____________

7. Were the patient referred from health facility

Yes ☐ No ☐

8. What treatment(s) did the patient receive before being referred? (Tick all the boxes that have been collected)

Fluids ☐ Antibiotics ☐ Source control ☐ Monitoring ☐ Not recorded ☐

9. What was the patient's pregnancy status when infection was first suspected/diagnosed?

<12 weeks ☐ 28+ weeks ☐ Miscarriage/ Abortion within the last 6 weeks ☐

12-28 ☐ Post-natal (up to 6 weeks) ☐

10. What type of infection was suspected? (Tick one or more options)

Offensive vaginal discharge ☐ Breast swelling/pain ☐

Vaginal bleeding ☐ Cough/shortness of breath/sore throat ☐

Delay in uterine involution ☐ Discharging wound/wound dehiscence ☐

Abnormal pain ☐ Swollen /painful cannula site ☐

Urinary symptoms ☐ Fever /rigors/malaise ☐

Vomiting/diarrhoea ☐ Headache/Neck stiffness/rash ☐

Others, Please state ____________

For peer review only

FORM 3: INDIVIDUAL PATIENT FORM **PATIENT ID NUMBER** ☐☐☐-☐☐☐

11. Did the patient have any of the following observations at time infection was first suspected/diagnosed (see q1)?

	Yellow trigger		Red trigger		No abnormal Observation	Not recorded
Respiratory rate	21-24		25 or more	10 or less	11 - 20	
	☐		☐	☐	☐	☐
Oxygen Saturations			94 or less Or needing oxygen		95 or more	
			☐		☐	☐
Temperature	38 or more	35.9 or less			36.0 to 37.9	
	☐	☐			☐	☐
Heart rate	100-119	40-49	120 or more	39 or less	50 – 99	
	☐	☐	☐	☐	☐	☐
Systolic Blood Pressure	140-159	90-99	160 or more	89 or less	100 -139	
	☐	☐	☐	☐	☐	☐
Diastolic Blood Pressure	90-109		110 or more	39 or less	40 – 89	
	☐		☐	☐	☐	☐
Not passed urine	In last 12-18 hours		In over 18 hours or less than 0.5ml/kg/hr.		12 hours or less	
	☐		☐		☐	☐
Appearance	Looks unwell				Look well	
	☐				☐	☐
Mental state			Not alert		Alert	
			☐		☐	☐

Date of observation [d][d] - [m][m] - [y][y][y][y]

☐ or tick if not recorded

Time of observation [h][h] [m][m]

☐ or tick if not recorded

For peer review only - http://bmjopen.bmj.com/site/about/guidelines.xhtml

FORM 3: INDIVIDUAL PATIENT FORM **PATIENT ID NUMBER** ☐☐☐-☐☐☐

12. At the time the decision tool was initiated (if intervention phase) or if not used that the infection was most serious there were:

Yellow flags ___ (enter number)

Red flags ___ (enter number)

If 2 or more yellow flags, or 1 or more red flags was the FAST-M bundle initiated (if intervention phase)?

Yes ☐ No ☐

13. If one or more red flags present? Was the FAST-M bundle initiated (within 1 hour) (if intervention phase)?

Yes ☐ No ☐
Not recorded ☐ Go to question 12

14. If 2 or more yellow flags were present, did a review take place within 3 hours?

Yes ☐ No ☐ Not recorded ☐

15. Was the patient an inpatient for 24 hours or more prior to infection being suspected/ diagnosed?

Yes ☐ No ☐

16. If yes, During the last 24 hours or more, please state the number of times each observation has been recorded prior to infection being suspected / diagnosed:

	Number of times
Respiratory rate	
Temperature	
Heart rate	
Systolic BP	

	Number of times
Diastolic BP	
Urine output	
Mental state	
Fetal heart rate	

For peer review only

FORM 3: INDIVIDUAL PATIENT FORM **PATIENT ID NUMBER** ☐☐☐-☐☐☐

17. At the first time that a yellow or red trigger occurred, was action taken within 3 hours (if two or more yellow flags) or within 1 hour (if red)?

Yes ☐ No ☐ Not Recorded ☐

18. What action was taken (answer yes or no for each option)?

	Yes	No
Medical Officer informed	☐	☐
Nurse in charge informed	☐	☐
Resident Doctor informed	☐	☐
Treatment started	☐	☐
Consultant Doctor Informed	☐	☐
Nurse	☐	☐
Decision tool started, if intervention phase?	☐	☐

Others (please state) ________________

19. Who took action (answer yes or no for each option)?

	Yes	No
	☐	☐
Medical Officer	☐	☐
Resident Doctor	☐	☐
Consultant Doctor	☐	☐
Medical assistant	☐	☐

	Yes	No
Nurse	☐	☐
Nurse In-charge	☐	☐

20. If the patient was in an outpatient setting was the patient subsequently admitted?

Yes ☐ No ☐ N/A ☐

21. Was the patient commenced on an observation chart?

Yes ☐ No ☐

For peer review only

FORM 3: INDIVIDUAL PATIENT FORM **PATIENT ID NUMBER** ☐☐☐-☐☐☐

22. In the 24 hours or more after suspecting maternal infection, did the patient have their observations repeated WHILST they were awaiting the clinical review?

Yes ☐ No ☐ No, because review happened at the same time ☐

Not Done ☐

	½ Hourly	Hourly	Less Frequent than hourly
Respiratory rate			
Temperature			
Heart rate			
Systolic BP			

	½ Hourly	Hourly	Less Frequent than hourly
Diastolic BP			
Urine output			
Mental state			
Fetal heart rate			

23. Did the patient receive a clinical review within 1 or 3 hours' review of recognition of maternal infection/sepsis?

Yes ☐ No ☐

Date and time of review d d - m m - y y y y h h : m m

For peer review only

FORM 3: INDIVIDUAL PATIENT FORM **PATIENT ID NUMBER** ☐☐☐-☐☐☐

a. Did the patient receive a clinical review within 3 hours? Yes ☐ No ☐
b. Did the patient receive a clinical review within 1 hour? Yes ☐ No ☐

24. Following the clinical review was the specified frequency of observations given?
Yes ☐ No ☐

25. Did the patient undergo observations at this specified frequency?
Yes ☐ No ☐

26. Was the patient treated for maternal infection/sepsis?
Yes ☐ No ☐

27. If intervention phase was the FAST-M decision tool was used for the patient?
Yes ☐ No ☐

MANAGEMENT

28. Were IV fluids given?

Yes ☐ No ☐ Why not? ______________________________

Date of started: d d - m m - y y y y   Time of started h h m m

☐ or tick if not recorded   ☐ or tick if not recorded

29. Were antibiotics administered within one hour?

Yes ☐ No ☐ Why not? ______________________________

Date of administered: d d - m m - y y y y   Time of administered: h h m m

☐ or tick if not recorded   ☐ or tick if not recorded

For peer review only

FORM 3: INDIVIDUAL PATIENT FORM

PATIENT ID NUMBER ☐☐☐ - ☐☐☐

SOURCE CONTROL/ IDENTIFICATION

30. Was an attempt made to identify the source of infection?

Yes ☐ No ☐ Why not? ______________________________

Date: d d - m m - y y y y Time: h h m m

☐ or tick if not recorded ☐ or tick if not recorded

What attempts were made to identify the source?

	Yes	No		Yes	No		Yes	No
Clinical history	☐	☐	HIV test	☐	☐	Imaging (abdominal, chest)	☐	☐
Clinical examination	☐	☐	Malaria test	☐	☐	Lumbar puncture	☐	☐
Blood test (if available)	☐	☐	Urine sample	☐	☐	Sputum sample	☐	☐
Blood cultures	☐	☐	Swabs (wound, vagina, throat)	☐	☐	Others (please state) ________________		

31. Was the source identified?

Yes ☐ No ☐

Date: d d - m m - y y y y Time: h h m m

☐ or tick if not recorded ☐ or tick if not recorded

For peer review only

FORM 3: INDIVIDUAL PATIENT FORM **PATIENT ID NUMBER** ☐☐☐-☐☐☐

What was the source of infection?

	Yes	No		Yes	No		Yes	No
Breast	☐	☐	Malaria	☐	☐	Peritonitis	☐	☐
Chorioamnionitis	☐	☐	Meningitis/ cerebral infection	☐	☐	Respiratory infection	☐	☐
Endometritis	☐	☐	Other wound/ skin infection	☐	☐	Urinary Tract Infection	☐	☐
Infected Cannula/ line	☐	☐	Other (please state)________________________					

32. Was the source treated?

Yes ☐ No ☐ Why not? ________________________________

Date: d d - m m - y y y y Time: h h m m

☐ or tick if not recorded ☐ or tick if not recorded

How was it treated?

	Yes	No		Yes	No		Yes	No
Delivery of the baby	☐	☐	Removal of infected cannula/line	☐	☐	Incision and drainage	☐	☐
Removal of retained products of conception	☐	☐	Targeted antibiotics	☐	☐	Percutaneous drainage	☐	☐
Hysterectomy	☐	☐	Malaria treatment	☐	☐	Debridement of wound	☐	☐
			Antivirals	☐	☐	Laparotomy and washout	☐	☐
						Other (Please state) ________________		

For peer review only

FORM 3: INDIVIDUAL PATIENT FORM

PATIENT ID NUMBER ☐☐☐-☐☐☐

TRANSPORT

33. Was there a need to transport the patient to a higher level of care considered?

Yes ☐ No ☐ Why not? ____________________ Not recorded ☐

Date: d d - m m - y y y y Time: h h m m

☐ or tick if not recorded ☐ or tick if not recorded

34. Was the patient transferred?

Yes ☐ No ☐ Why not? ____________________ Not recorded ☐

Date requested: d d - m m - y y y y Time requested: h h m m

☐ or tick if not recorded ☐ or tick if not recorded

Date patient left the facility: d d - m m - y y y y Time: h h m m

☐ or tick if not recorded ☐ or tick if not recorded

35. Where was the patient transferred to? ____________________________________

36. Was a transfer letter sent with the patient?

Yes ☐ No ☐ Why not? ____________________

For peer review only

FORM 3: INDIVIDUAL PATIENT FORM **PATIENT ID NUMBER** ☐☐☐-☐☐☐

MONITORING

37. Did the patient have ongoing monitoring commenced after the recognition of maternal suspected/sepsis?

Yes ☐ No ☐ → N/A (24hrs hadn't elapsed) ☐

	1/2 hourly or Less	Hourly or Less	Daily or More	Less than Daily	not done
Respiratory rate	☐	☐	☐	☐	☐
Temperature	☐	☐	☐	☐	☐
Heart rate	☐	☐	☐	☐	☐
Systolic BP	☐	☐	☐	☐	☐

	½ hourly or Less	Hourly or Less	Daily or More	Less than Daily	not done
Diastolic BP	☐	☐	☐	☐	☐
Urine output	☐	☐	☐	☐	☐
Neuro Response	☐	☐	☐	☐	☐
Fetal heartrate	☐	☐	☐	☐	☐

For peer review only

FORM 3: INDIVIDUAL PATIENT FORM **PATIENT ID NUMBER** ☐☐☐-☐☐☐

PATIENT OUTCOMES

38. Did the patient have any of the following complications recorded during their treatment?

	Yes	No
Pulmonary oedema	☐	☐
Blood transfusion	☐	☐
Return to theatre	☐	☐
Allergic reaction	☐	☐

	Yes	No
Change in mental state	☐	☐
Reduced urine output (no output for >18 hours or <0.5ml/kg/hr)	☐	☐
Other	☐	☐
Please state	☐	☐

39. Was the patient admitted to high dependency/ intensive care during their treatment?

Yes ☐ No ☐

No. of days: ☐ Days

40. Did the patient have any of the following near miss events recorded during their stay?

Cardiovascular dysfunction		
**Event**	**Yes**	**No**
Shock	☐	☐
Cardiac arrest	☐	☐
Severe hypo perfusion (lactate >5mmol/L)	☐	☐
Severe acidosis (pH<7.1)	☐	☐
Use of continuous vasoactive drugs	☐	☐
Cardio-pulmonary resuscitation	☐	☐
**Respiratory dysfunction**		
Acute cyanosis	☐	☐
Gasping	☐	☐
Severe tachypnea (respiratory rate >40)	☐	☐
Severe bradypnea (respiratory rate <6)	☐	☐
Severe hypoxaemia (O2 sats <90% for >60 mins or PAO2/FiO2 <200)	☐	☐
Intubation and ventilation not related to anaesthesia	☐	☐

For peer review only

FORM 3: INDIVIDUAL PATIENT FORM **PATIENT ID NUMBER** ☐☐☐-☐☐☐

PATIENT OUTCOMES

**Renal dysfunction**		
Oliguria non-responsive to fluids or diuretics	☐	☐
Creatinine >300 µmol/ml or >3.5 mg/dL	☐	☐
Dialysis for acute renal failure	☐	☐
**Coagulation dysfunction**		
Failure to form clots	☐	☐
Severe acute thrombocytopenia (50,000 platelets/ml)	☐	☐
Massive transfusion of blood or red cells (≥5 units)	☐	☐
**Hepatic dysfunction**		
Jaundice in the presence of pre-eclampsia	☐	☐
Severe acute hyperbilirubinemia (bilirubin>100 µmol/L or >6.0 mg/dL)	☐	☐
**Neurological dysfunction**		
Prolonged unconsciousness or coma (lasting >12 hours)	☐	☐
Stroke	☐	☐
Uncontrollable fit / status epilepticus	☐	☐
Global paralysis	☐	☐
**Uterine dysfunction**		
Hysterectomy due to uterine infection or haemorrhage	☐	☐

For peer review only

FORM 3: INDIVIDUAL PATIENT FORM **PATIENT ID NUMBER** ☐☐☐ - ☐☐☐

PATIENT OUTCOMES

41. Date of final discharge from hospital/health centre: d d - m m - y y y y

42. Total number of days spent in hospital: ☐ days

43. What was the outcome of the patient at discharge?

Alive and well, no follow-up required ☐

Alive and well, follow-up arranged ☐

Death ☐

Date of death: d d - m m - y y y y

Time of Death: h h : m m

Cause of death: ______________________

PREGNANCY OUTCOMES (if event occurred during same admission)

Tick if not applicable ☐

44. What was the pregnancy outcome?

Miscarriage ☐

Ectopic pregnancy ☐

Induced abortion ☐

Date occurred or diagnosed: d d - m m - y y y y

Live birth ☐

Still birth (> 28 weeks) ☐

Pregnancy terminated before admission ☐

For peer review only

FORM 3: INDIVIDUAL PATIENT FORM **PATIENT ID NUMBER** ☐☐☐ - ☐☐☐

45. Was monitoring of the new-born commenced?

Yes ☐ No ☐ N/A ☐

Date: d d - m m - y y y y Time: h h m m

☐ or tick if not recorded ☐ or tick if not recorded

46. Was fetal monitoring done if the patient was antenatal?

Yes ☐ No ☐ N/A ☐

Date: d d - m m - y y y y Time: h h m m

☐ or tick if not recorded ☐ or tick if not recorded

For peer review only

FORM 3: INDIVIDUAL PATIENT FORM

PATIENT ID NUMBER ☐☐☐-☐☐☐

NEONATAL OUTCOMES AT BIRTH

Tick if not applicable ☐

47. Gestation: ☐ Months OR ☐ weeks ☐ days

48. Weight: ☐ g Not recorded ☐

49. Did the baby have any of the following complications?

	Yes	No	Not recorded
Resuscitation	☐	☐	☐
Agpars below 7 at 5 mins after birth	☐	☐	☐
Admitted to neonatal unit/special care	☐	☐	☐
Other (Please state)	☐	☐	____________

50. Was the baby thought to have sepsis?

Yes ☐ No ☐

Did the baby receive antibiotic prophylaxis anyway?

Yes ☐ No ☐

Were they treated for sepsis?

Yes ☐ No ☐ Not recorded

51. What was the outcome of the baby at discharge?

Alive and well, no follow-up required ☐

Alive and well, follow-up required ☐

Death ☐

Date of Death d d - m m - y y y y

Time of Death: h h m m

Cause of Death ____________

For peer review only

FORM 3: INDIVIDUAL PATIENT FORM **PATIENT ID NUMBER** ☐☐☐-☐☐☐

Completed by: ______________________________

Role: ______________________________

Signature: ______________________________ Date: DD / MM / YYYY

You must have signed the Site Signature & Delegation Log

For peer review only

Supplemental File 5

APPENDIX FIGURE 14 – Participant Study ID Number: ___/_____– PATIENT CONSENT FORM (ENGLISH) for patients agreeing to access of their notes

Title of study: Extension of the FAST- M maternal sepsis bundle in Pakistan, a feasibility study

What is the purpose of the overall study?

We are developing a way of improving the care of women with pregnancy or birth related infections around the world.

We are introducing three things: 1. a chart to help the nurses record your vital signs and detect infection before it becomes worse 2. A tool to help the medical staff treat infection 3. A training day to teach the medical staff about pregnancy or birth related infections

We hope that this will make caring for patients with pregnancy or birth related infections easier.

Why have I been invited to participate?

We are asking all patients pregnant or recently pregnant patients who are receiving treatment in hospital or who have an infection to participate. Being invited to take part does not necessarily mean you have an infection.

What will I have to do if I take part?

Nothing. If you agree to take part, you will receive the normal care. All we ask is that our research team from Aga Khan University Hospital can look at your medical notes to see what happened to you whilst you were in hospital. The information from your medical notes will help us decide if our study is working.

Do I have to take part?

No you do not have to take part it is your decision. If you don't want to take part that is fine, it will not affect how you are cared for when you are in hospital.

Will my taking part in the study be kept confidential?

Yes. All the information from your notes will be stored securely on a password protected computer system, which will be backed-up in a separate location to keep this information safe.

What are the possible benefits of taking part?

By giving us permission to look at your notes we hope that we will be able to find out if the study is working. If the study works, then this will hopefully help to improve the care of mothers with infections.

What will happen if I don't want to carry on with the study?

You may withdraw from the study without giving a reason for up to one month after. If you wish to withdraw we will delete all the information we had collected about you.

What if there is a problem?

For peer review only

If you have a concern about any aspect of this study, you can speak to the researchers, who will do their best to answer your questions. Their contact details are on the next page.

Who is organizing the research?

This study is being carried out by the Aga Khan University Hospital(AKUH), Pakistan in affiliation with University of Birmingham, UK and University of Liverpool, UK.

AKUH team will be facilitating the day to day running of the study, including looking at your medical notes and collecting the information.

Who has reviewed the study?

This study has been reviewed by the National Bioethics Committee Pakistan and AKUH Research Ethics Committee.

Contact details:

Lead Facilitator: Dr Sheikh Irfan Ahmed, Senior Instructor, AKUH National stadium road, Karachi

Email: sheikh.irfan@aku.edu   Telephone number: +92-021-34864650

Registered study ID number:

PLEASE INITIAL THE BOXES IF YOU AGREE WITH EACH SECTION:

1. I understand that my participation is voluntary and that I am free to withdraw up to one month after my participation without giving any reason. ☐
2. I agree for my notes to be accessed by the AKUH research team. I understand that all data collected about me will be anonymised and will not contain any identifiable information. ☐
3. I understand that the researchers might publish the results of this study. I give permission for anonymized information from my notes to be used for this purpose. ☐
4. I know how to contact the research team if I need to. ☐
5. I am happy for information about me might also be retained to include it anonymously in future studies
6. I agree to participate in this study. ☐

SIGNATURES:

____________________________   ____________   ________________________ Patient name and surname

Date   Signature / Thumbprint

For peer review only

___________________________ ___________ _______________________ Name and Surname of person

Date Signature taking consent

Participant Study ID Number: _____/_____

For peer review only

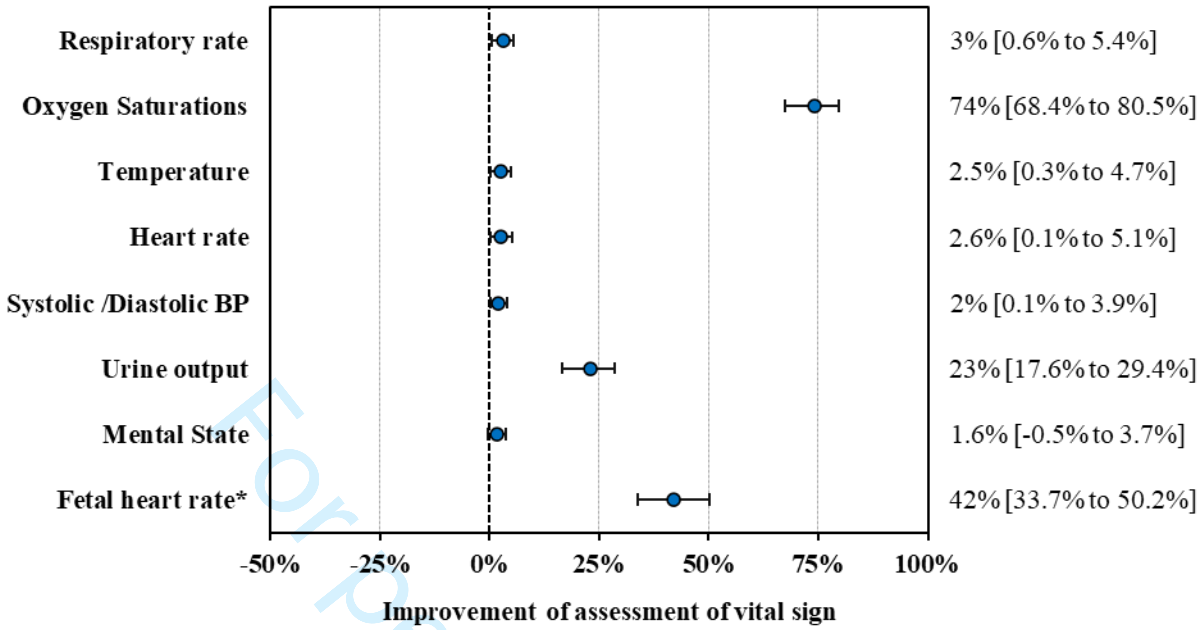

Supplement Figure 6: Patient's assessment of vital signs on admission (n=439)

For peer review only

Supplemental File 7

Table: Characteristics of suspected maternal sepsis patients (n=138)

Variables	Baseline n=60	Intervention n=78
**Gestational at presentation**		
<12 weeks	2(3.3%)	0(0%)
12-28 weeks	2(3.3%)	1(1.3%)
>28 weeks	31(51.7%)	38(48.7%)
Post-natal (up to 6 weeks)	25(41.7%)	39(50%)
**Abnormal Trigger**		
Respiratory Rate (>25 or <10 per minute)	41(68.3%)	41(52.6%)
Temp (>38 or <35.9 °C)	25(41.7%)	35(44.9%)
Heart Rate (>120 or <39 beats per minute)	32(53.3%)	38(48.7%)
Systolic Blood Pressure (>160 or <89 mmhg)	43(71.7%)	43(55.1%)
Diastolic Blood Pressure (>110 or <39 mmhg)	35(58.3%)	38(48.7%)
Urine Output (In over 18 hours or less than 0.5ml/kg/hr)	4(6.7%)	4(5.1%)
Mental State (Not alert)	9(15%)	28(35.9%)
Appearance (Looks unwell)	7(11.7%)	49(62.8%)
**Source of maternal infection/sepsis***		
Breast	1(1.7%)	1(1.3%)
Chorioamnionitis	13(21.7%)	7(9%)
Endometritis	4(6.7%)	2(2.6%)
Infected Cannula/ line	1(1.7%)	0(0%)
Malaria	0(0%)	5(6.4%)
Meningitis/ cerebral infection	1(1.7%)	0(0%)
Other wound/ skin infection	3(5%)	5(6.4%)
Peritonitis	2(3.3%)	7(9%)
Respiratory infection	29(48.3%)	14(17.9%)
Urinary Tract Infection	9(15%)	6(7.7%)
Others	13(21.7%)	31(39.7%)
Unknown	7(11.7%)	14(17.9%)

*Thirty patients had multiple infection

For peer review only

Supplemental file 8

Table: Laboratory investigations of suspected maternal sepsis patients

Laboratory Investigations	Baseline phase (n=60)	Intervention phase (n=78)
Blood test (if available)	55 (92%)	78 (100%)
Blood cultures	27 (45%)	67 (86%)
HIV test	52 (87%)	72 (92%)
Malaria test	1 (2%)	21 (27%)
Urine sample	10 (17%)	63 (81%)
Swabs (wound, vagina, throat)	9 (15%)	2 (3%)
Imaging (abdominal, chest)	8 (13%)	38 (49%)
Lumbar puncture	0 (0%)	2 (3%)
Sputum sample	2 (3%)	2 (3%)

For peer review only

Supplemental file 9

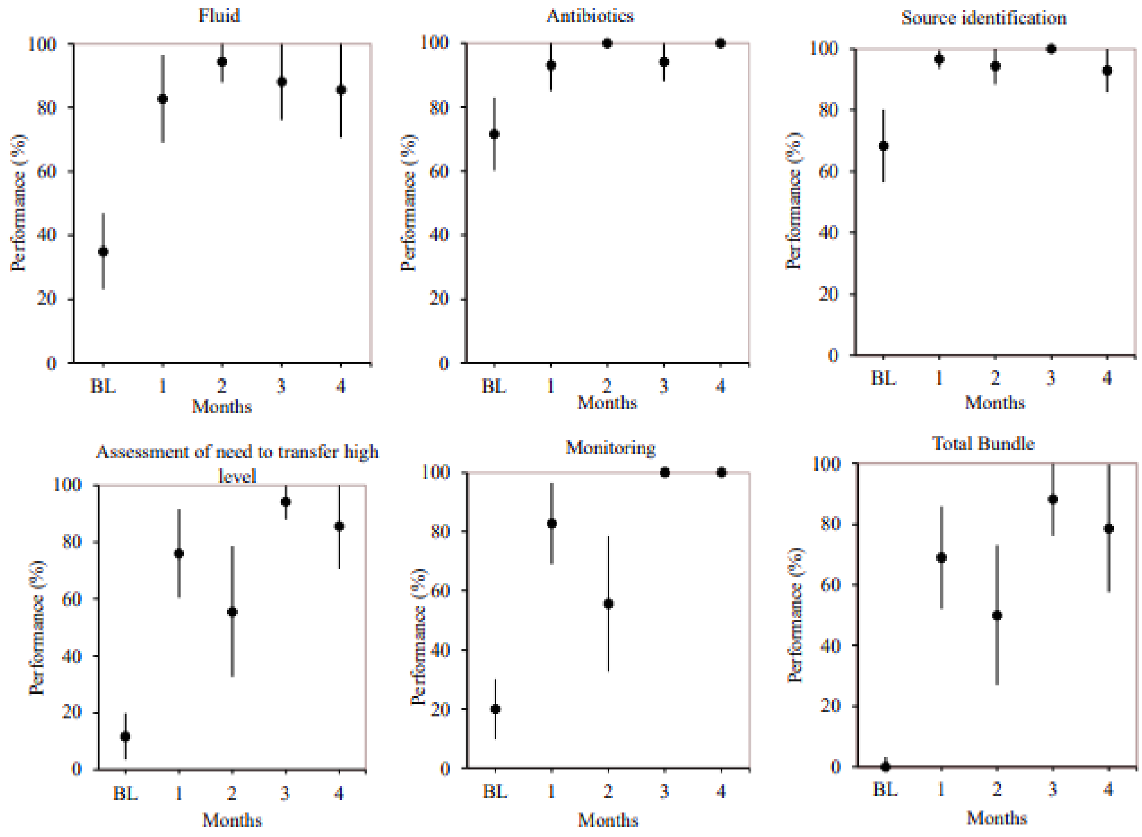

For peer review only

Supplemental file 10

Table: Assessment of Maternal statistics (from facility audit CRF1)

Total Admissions (N=24,000)	January to June 2021 (n=11205)	July to December 2021 (n=12795)	P-value
Maternal Sepsis	210 (1.8%)	160 (1.2%)	.0005
Maternal Mortality	159 (1.4%)	120 (0.9%)	.001
Post-Partum Hemorrhage	110 (0.9%)	175 (1.36%)	.006
Antepartum Hemorrhage	194 (1.73%)	110 (0.85%)	.0005
Severe Pre-Eclampsia	133 (1.18%)	88 (0.68%)	.0005
Uterine Rupture	36 (0.32%)	28 (0.21%)	.125

eview only

STROBE Statement—checklist of items that should be included in reports of observational studies

	Item No	Recommendation	Page No
**Title and abstract**	1	(*a*) Indicate the study's design with a commonly used term in the title or the abstract	1,3
		(*b*) Provide in the abstract an informative and balanced summary of what was done and what was found	3
**Introduction**			
Background/rationale	2	Explain the scientific background and rationale for the investigation being reported	5-6
Objectives	3	State specific objectives, including any prespecified hypotheses	6
**Methods**			
Study design	4	Present key elements of study design early in the paper	7
Setting	5	Describe the setting, locations, and relevant dates, including periods of recruitment, exposure, follow-up, and data collection	7-12
Participants	6	(*a*) *Cohort study*—Give the eligibility criteria, and the sources and methods of selection of participants. Describe methods of follow-up<br>*Case-control study*—Give the eligibility criteria, and the sources and methods of case ascertainment and control selection. Give the rationale for the choice of cases and controls<br>*Cross-sectional study*—Give the eligibility criteria, and the sources and methods of selection of participants	7-8
		(*b*) *Cohort study*—For matched studies, give matching criteria and number of exposed and unexposed<br>*Case-control study*—For matched studies, give matching criteria and the number of controls per case	
Variables	7	Clearly define all outcomes, exposures, predictors, potential confounders, and effect modifiers. Give diagnostic criteria, if applicable	11
Data sources/ measurement	8*	For each variable of interest, give sources of data and details of methods of assessment (measurement). Describe comparability of assessment methods if there is more than one group	12
Bias	9	Describe any efforts to address potential sources of bias	11-12
Study size	10	Explain how the study size was arrived at	12-13
Quantitative variables	11	Explain how quantitative variables were handled in the analyses. If applicable, describe which groupings were chosen and why	12
Statistical methods	12	(*a*) Describe all statistical methods, including those used to control for confounding	12
		(*b*) Describe any methods used to examine subgroups and interactions	
		(*c*) Explain how missing data were addressed	
		(*d*) *Cohort study*—If applicable, explain how loss to follow-up was addressed<br>*Case-control study*—If applicable, explain how matching of cases and controls was addressed<br>*Cross-sectional study*—If applicable, describe analytical methods taking account of sampling strategy	

For peer review only

(*e*) Describe any sensitivity analyses

Continued on next page

For peer review only

**Results**			
Participants	13*	(a) Report numbers of individuals at each stage of study—eg numbers potentially eligible, examined for eligibility, confirmed eligible, included in the study, completing follow-up, and analysed	8,12-13
		(b) Give reasons for non-participation at each stage	12
		(c) Consider use of a flow diagram	8
Descriptive data	14*	(a) Give characteristics of study participants (eg demographic, clinical, social) and information on exposures and potential confounders	13
		(b) Indicate number of participants with missing data for each variable of interest	12-13
		(c) *Cohort study*—Summarise follow-up time (eg, average and total amount)	NA
Outcome data	15*	*Cohort study*—Report numbers of outcome events or summary measures over time	*NA*
		Case-control study—Report numbers in each exposure category, or summary measures of exposure	*NA*
		Cross-sectional study—Report numbers of outcome events or summary measures	12-15
Main results	16	(*a*) Give unadjusted estimates and, if applicable, confounder-adjusted estimates and their precision (eg, 95% confidence interval). Make clear which confounders were adjusted for and why they were included	NA
		(*b*) Report category boundaries when continuous variables were categorized	NA
		(*c*) If relevant, consider translating estimates of relative risk into absolute risk for a meaningful time period	NA
Other analyses	17	Report other analyses done—eg analyses of subgroups and interactions, and sensitivity analyses	12-15
**Discussion**			
Key results	18	Summarise key results with reference to study objectives	15
Limitations	19	Discuss limitations of the study, taking into account sources of potential bias or imprecision. Discuss both direction and magnitude of any potential bias	19-20
Interpretation	20	Give a cautious overall interpretation of results considering objectives, limitations, multiplicity of analyses, results from similar studies, and other relevant evidence	15-20
Generalisability	21	Discuss the generalisability (external validity) of the study results	20
**Other information**			
Funding	22	Give the source of funding and the role of the funders for the present study and, if applicable, for the original study on which the present article is based	21

*Give information separately for cases and controls in case-control studies and, if applicable, for exposed and unexposed groups in cohort and cross-sectional studies.

Note: An Explanation and Elaboration article discusses each checklist item and gives methodological background and published examples of transparent reporting. The STROBE checklist is best used in conjunction with this article (freely available on the Web sites of PLoS Medicine at http://www.plosmedicine.org/, Annals of Internal Medicine at http://www.annals.org/, and Epidemiology at http://www.epidem.com/). Information on the STROBE Initiative is available at www.strobe-statement.org.

For peer review only
